# Mechanistic Insights of BHT-Mg-Catalyzed Ethylene Phosphate’s Coordination Ring-Opening Polymerization: DFT Modeling and Experimental Data

**DOI:** 10.3390/polym10101105

**Published:** 2018-10-06

**Authors:** Ilya Nifant’ev, Andrey Shlyakhtin, Maxim Kosarev, Stanislav Karchevsky, Pavel Ivchenko

**Affiliations:** 1 M.V.Lomonosov Moscow State University, Chemistry Department, 1-3 Leninskie Gory, Moscow 119991, Russia; shlyahtinav@mail.ru (A.S.); komrad.kosarev.maksim@gmail.com (M.K.); phpasha1@yandex.ru (P.I.); 2 A.V. Topchiev Institute of Petrochemical Synthesis RAS, 29 Leninsky Pr., Moscow 119991, Russia; 3 Joint-stock company “Institute of petroleum refining and petrochemistry”, 12 Iniciativnaya Str., Ufa, Republic of Bashkortostan 450065, Russia; st_karchevsky@mail.ru

**Keywords:** ring-opening polymerization, ethylene phosphate, lactide, DFT, magnesium phenoxide

## Abstract

Poly(ethylene phosphates) are promising polymers for use in biomedical applications. Catalytic ring-opening polymerization (ROP) of cyclic ethylene phosphate monomers (CEPMs) is the most effective approach for obtaining these polymers. The mechanism of coordination ROP of CEPMs remains unclear. We report, for the first time, the results of DFT modeling of CEPM ROP. In these calculations by Gaussian-09 program package with the B3PW91/DGTZVP basis set, we explored methyl ethylene phosphate (MeOEP) ROP catalyzed by dimeric and monomeric catalytic species derived from heteroleptic complex [(BHT)Mg(μ-OBn)(THF)]_2_ (**Mg1**, BHT = 2,6-di-*tert*-butyl-4-methylphenolate). Analysis of the reaction profiles for the binuclear and mononuclear reaction mechanisms allowed us to conclude that the ROP of MeOEP is preferentially catalyzed by mononuclear Mg complexes. This estimation was confirmed by comparative polymerization experiments using MeOEP and traditional monomers ε-caprolactone (εCL), racemic lactide (*rac*-LA), and *l*-lactide (*l*-LA) initiated by **Mg1**. ROP of MeOEP proceeds at an extremely high rate due to the substantially lower activation barrier calculated for mononuclear mechanism in comparison with that of cyclic esters that polymerize without the dissociation of BHT-Mg binuclear species. We also demonstrated the use of MeOEP as a "monomerization" agent in the synthesis of MeOEP-lactide block copolymers. Comparison of the multiple acceleration of *l*-LA ROP after MeOEP prepolymerization and formation of atactic PLA blocks in *rac*-LA polymerization with the heterotactic PLA formation during **Mg1**-catalyzed homopolymerization also confirmed the mononuclear nature of the polyphosphate-containing catalytic particles.

## 1. Introduction

**Scheme 1:**
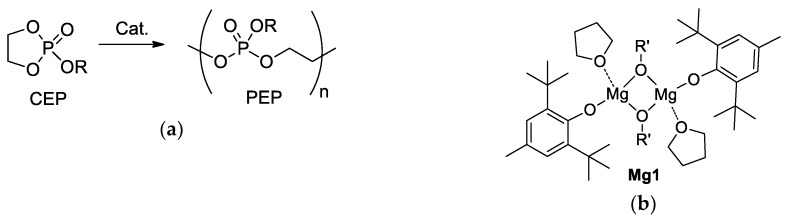
(**a**) Ring-opening polymerization (ROP) of ethylene phosphates; (**b**) Efficient BHT-Mg catalysts.

Poly(ethylene phosphates), or PEPs are a prospective group of biocompatible polymers with controlled hydrophilicity and biodegradability. The structural similarity of PEPs and nucleic and teichoic acids favors the use of these polymers in future biomedical materials [1,2,3,4,5,6,7,8,9]. Ring-opening polymerization (ROP) of cyclic ethylene phosphate monomers (CEPMs) is an efficient method for the preparation of PEPs (Scheme 1a) [10,11,12,13,14,15]. Recently, we demonstrated the high efficiency of BHT-Mg (BHT = 2,6-di-*tert*-butyl-4-methylphenolate) alkoxy complexes (**Mg1**, Scheme 1b) in the ROP of ethylene phosphates with the formation of linear and branched polymers [16,17,18]. Complexes with the formula **Mg1** (R′ = Et, Bn) are effective in the ROP of traditional monomers, which are cyclic esters [19]. In our recent paper [20], we reported the results of an experimental and theoretical study of ε-caprolactone (εCL) and lactide (LA) polymerization, that included density functional theory (DFT) modeling of the reaction profiles. These results allowed us to postulate the preference for the binuclear coordination mechanism for εCL and LA polymerization, to explain the stereocontrol in *rac*-LA ROP with the formation of heterotactic PLA, and enabled us to understand the significance of carbonyl chelation in the ROP for lactide.

On the basis of the extremely high efficiency of **Mg1** in ethylene phosphate polymerization [16], we proposed that, for CEPMs, the reaction mechanism may differ qualitatively from the εCL and LA ROP mechanisms. To the best of our knowledge, ROP of cyclic phosphates has never been studied with DFT. In this paper, we present the results of DFT modeling of the ROP of methyl ethylene phosphate (MeOEP, R = Me in Scheme 1a) catalyzed by **Mg1**. To confirm the results of the calculations, we performed a series of polymerization experiments to compare the reaction rates and free activation energies for MeOEP and traditional substrates, namely, cyclic esters εCL, *rac*-LA, and *l*-LA.

## 2. Materials and Methods

The initial Cartesian coordinates of the stationary points were generated by the PRIRODA software (version 4.0, Moscow, Russia) [21] using the 3ζ basis. The final calculations (structure optimization and determination of the thermodynamic parameters) were carried out using the Gaussian 09 program [22] for the gas phase at 298.15 K. Transition state configurations were found by energy scanning with sequential changing of key geometric parameters with a step of 0.01 Å, Berny optimization, and were confirmed by IRC. The B3PW91 hybrid exchange-correlation functional [23,24] and the DGTZVP basis set [25,26] were used in the optimizations. These parameters were used in our earlier DFT modeling of εCL and LA ROP [20], and their applicability for cyclic phosphates was confirmed by comparing the MeOEP geometries calculated using different functional/basis pairs (see the Supporting Information, Figure S1 and Tables S1, S2) to the X-ray data [27]. The advantages of the B3PW91 functional, as opposed to the traditional B3LYP, have been examined elsewhere [28,29]; Sumerlin et al. pointed that the conventional B3LYP functional generates adequate geometries but performs poorly in energy calculations [30]. The B3PW91 functional was previously used to successfully model ROP [20,31,32,33,34,35]. The ease and high efficiency of the DGTZVP basis set for molecules not containing transition metals have also been demonstrated in several publications [36,37,38].

All of the synthetic and polymerization experiments were performed under purified argon atmosphere. CH_2_Cl_2_ was washed with aqueous Na_2_CO_3_, stirred with CaCl_2_ powder, refluxed over CaH_2_ for 8 h and distilled. *l*-lactide and *rac*-lactide (Sigma-Aldrich/Merck, Darmstadt, Germany) were purified by recrystallization and subsequent sublimation. ε-Caprolactone was distilled prior to use under argon over CaH_2_. Methyl ethylene phosphate (MeOEP) [16] and [(BHT)Mg(μ-OBn)(THF)]_2_ (**Mg1**) [20] were synthesized according to the literature procedures.

CDCl_3_ (Cambridge Isotope Laboratories, Inc., D 99.8%) was distilled over P_2_O_5_ and stored over 4 Å molecular sieves. The ^1^H and ^31^P NMR spectra were recorded on a Bruker AVANCE 400 spectrometer (400 MHz) (Bruker Corporation, Billerica, MA, USA) at 20 °C. The chemical shifts are reported in ppm relative to the solvent residual peak (7.26 ppm).

Polymerization was carried out at 20 °С in a 1 M monomer solutions. A preheated glass ampoule was equipped with a magnetic stir bar and a septum, and then filled with dry argon. Cyclic substrate (10 mmol) was placed into the ampule. Then, CH_2_Cl_2_ was added to achieve the required concentration. Next, 0.2 ml of a 0.25 М (50 μmol) solution of **Mg1** catalyst (100:1 monomer/Mg ratio) in THF or CH_2_Cl_2_ was injected into the stirred monomer solution at the given temperature. After a certain time period, a 5-fold excess of acetic acid was injected into the ampoule to neutralize the catalyst and stop the process. The monomer conversion was determined using ^1^H NMR spectroscopy by integration of the monomer and polymer resonance signals:

for PCL, for C*H*_2_OC=O, δ = 4.2 ppm (monomer) and 4.0 ppm (polymer);

for PLA, for C*H*(CH_3_)OC=O, δ = 5.0 ppm (monomer) and 5.1–5.2 ppm (polymer);

for poly(MeOEP), for C*H*_2_O, δ = 4.4 ppm (monomer) and 4.2 ppm (polymer).

End-group analysis of ^1^H NMR spectra of polymers was used for the determination of *M*_n_^NMR^ (see the Supporting Information, Figures S2−S5).

Size exclusion chromatography (SEC) was performed on an Agilent PL-GPC 220 chromatograph equipped with a PLgel column (Agilent Technologies, Santa Clara, CA, United States), using THF (εCL, LA polymers) or DMF (MeOEP polymers) as the eluents (1 mL/min). The measurements were recorded with universal calibration according to polystyrene standards (εCL, LA polymers) or poly(ethylene glycol) standards (MeOEP polymers) at 40 °C. The obtained molecular weights were corrected by the factors of 0.56 (PCL) and 0.58 (PLA) [39].

## 3. Results

### 3.1. DFT Modeling of the Formation of the Catalytic Species

**Scheme 2:**
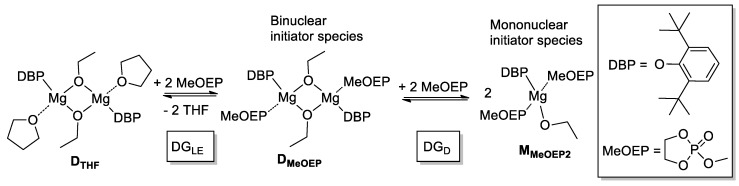
Ligand exchange and dissociation for **Mg1** complexes with the formation of dimeric and monomeric initiator species.

In our recent paper, [16] we established the living character of the polymerization of ethylene phosphates catalyzed by BHT-Mg initiators. This allowed us to propose a coordination mechanism for the **Mg1**-catalyzed ROP of MeOEP. To significantly reduce the calculation time, 2,6-di-*tert*-butylphenoxy (DBP) magnesium complexes with ethoxy groups were applied as initiator models. It was demonstrated in [20] that dimeric complexes **D_THF_** that are isostructural to **Mg1** (Scheme 2) can be affected by the donor molecules of MeOEP with the formation of dimeric catalytic species **D_MeOEP_** by the formula [(DBP)Mg(μ-OEt)(MeOEP)]_2_. Additional coordination of MeOEP leads to dissociation, obtaining monomeric complexes **M_MeOEP2_** with the formula (DBP)Mg(OEt)(MeOEP)_2_ (Scheme 2). The free energy of the dissociation is positive, and its value should be considered when comparing the energy profiles for the dinuclear and mononuclear ROP reaction pathways [20].

The relative free energy of ligand exchange (Δ*G*_LE_) was calculated as the difference in the free energies of **D_MeOEP_** and **D_THF_** with due consideration of the free energies of MeOEP and THF using the formula Δ*G*_LE_ = *G*${}_{298}^{o}$(**D_MeOEP_**) − *G*${}_{298}^{o}$(**D_THF_**) − 2*G*${}_{298}^{o}$(MeOEP) + 2*G*${}_{298}^{o}$(THF). We found that for MeOEP Δ*G*_LE_ = −2.5 kcal/mol. The relative free energy of dissociation was calculated according to the formula Δ*G*_D_ = *G*${}_{298}^{o}$(**M_MeOEP2_**) − ½*G*${}_{298}^{o}$(**D_MeOEP_**) − *G*${}_{298}^{o}$(MeOEP). We found that the formation of a monomeric complex is energetically unfavorable, Δ*G*_D_ = 10.1 kcal/mol. However, this value is not sufficient to unambiguously determine the preferred reaction mechanism. To establish the most likely reaction pathway of MeOEP polymerization, we performed detailed calculations for the initiation and propagation stages of the binuclear and mononuclear mechanisms.

### 3.2. DFT Modeling of the Mononuclear ROP Mechanism

#### 3.2.1. Initiation Stage

**Scheme 3:**
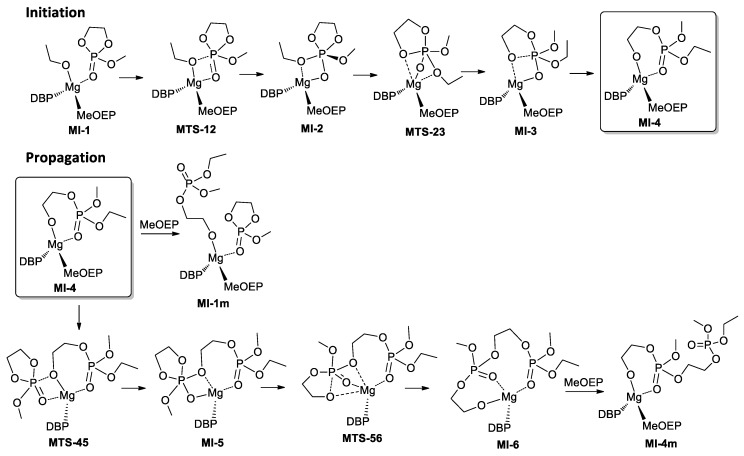
Key intermediates and transition states of MeOEP ROP catalyzed by mononuclear DBP-Mg complexes.

In DFT modeling of the initiation stage of the mononuclear mechanism (Scheme 3, top), tetrahedral complex (DBP)Mg(OEt)(MeOEP)_2_ was used as a starting stationary point **MI-1**. In terms of the coordination-insertion ROP mechanism, the reaction includes three stages: nucleophilic attack by alkoxide to the phosphorus atom, coordination of endocyclic oxygen to the Mg atom, and ring-opening.

**Figure 1:**
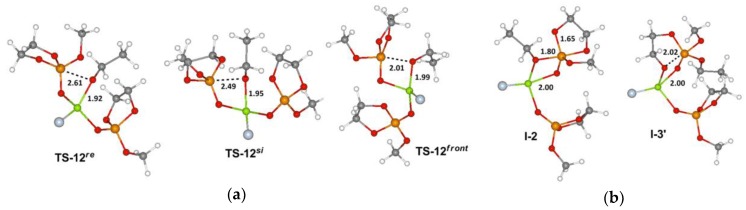
(**a**) Geometries of apical (*re*- and *si*-), and frontal transition states **MTS-12**; (**b**) Geometries of intermediates **MI-2** and **MI-3** (DBP fragments are omitted for clarity, DBP oxygen atoms are marked in blue).

It was previously shown that apical nucleophilic attack from the back side of one of the P–O ring bonds is preferable for cyclic ethylene phosphates [40]. We calculated the free energies for different geometries of the insertion transition states **MTS-12**. Calculations were performed for the complex **MI-1** with the (*R*)-configuration of the Mg atoms. Free energy Δ*G***_MI-1_** was minimal for the *re*-attack of the MeOEP molecule by an ethoxy group (6.6 kcal/mol), whereas the Δ*G*_M**I-1**_ for *si*-attack was 7.7 kcal/mol. Δ*G***_MI-1_** of the frontal transition state **MTS-12*^front^*** was 10 kcal/mol higher than the energies of apical transition states. We found that Δ*G***_MI-1_** of **MTS-12** is correlated with the EtO^…^P contact values (Figure 1), which are 2.61, 2.49, and 2.01 Å for **MTS-12*^re^***, **MTS-12*^si^***, and **MTS-12*^front^***, respectively.

The product of nucleophilic addition of the EtO group **MI-2** is a spirobicyclic orthophosphate complex with EtO^…^Mg coordination. Dissociation of this bond with the coordination between the Mg atom and endocyclic oxygen atom passes through the rotational transition state **MTS-23** (Δ*G*_MI-1_ = 8.5 kcal/mol) and results in complex **MI-3**. This intermediate has a strained condensed bicyclic structure, and endocyclic P–O bond breaks easily with the formation of the more stable complex **MI-4**. **MI-4** has minimal energy among the stationary points of the initiation stage (Δ*G*_MI-1_ = −7.0 kcal/mol).

#### 3.2.2. Propagation Stage

Two alternative pathways are possible for the further transformations of **MI-4**. The first is the coordination of the second MeOEP molecule with the opening of the seven-membered metallacycle leading to **MI-1m**, which is isostructural to **MI-1** (Scheme 3, middle). The formation of **MI-1m** via **MTS-41m** (Δ*G*_MI-1_ = 6.2 kcal/mol) finalizes the initiation stage. The difference in free energies between **MI-1m** and **MI-1** (Δ*G*_MI-1_ = −2.3 kcal/mol) is correlated with the experimental estimates for the energies of the MeOEP ring-opening (Δ*G* = −2.6 kcal/mol at 25 °С) [41]. At first glance, the structural similarity of **MI-1** and **MI-1m** allows the consideration of the sequence of the initiation stage **MI-1** → **MTS-12** → **MI-2** → **MTS-23** → **MI-3** → **MI-4** → **MI-1m** (Scheme 3, top) as a model for the chain growth stage and ROP as a whole. We used a similar model to create the reaction profile of the **Mg1**-catalyzed ROP of ε-CL [20]. However, in [20], considering the results of the DFT simulation of *rac*-lactide ROP, we found that a stable chelate complex that is similar to **MI-4** was formed during the first ring-opening. This complex had a significantly lower free energy compared to the alkoxy-initiator of the **MI-1** type, demonstrating the preference of the mononuclear reaction mechanism of the *rac*-lactide ROP involving chelate complexes.

We proposed the analogy between lactide and phosphate polymerization mechanisms, and performed DFT simulations of the chain propagation with chelated particles starting from **MI-4** (Scheme 3, bottom). In the first stage, an intramolecular nucleophilic attack of the alkoxy group on a phosphorus atom with the formation of a spirobicyclic intermediate **MI-5** goes through a low-energy transition state **MTS-45** (Δ*G*_M**I-4**_ = 0.2 kcal/mol). Complex **MI-5** then transforms into a ring-opening product **MI-6** through a rotational transition state **MTS-56**. The energy of this transition state (9.7 kcal/mol relative to **MI-4**) determines the activation barrier of the propagation sequence that models the ROP process. The coordination of another MeOEP molecule is accompanied by the loss of the coordination of one of the phosphate groups and leads to chelate **MI-4m**, that is isostructural with **MI-4**. Thus, the sequence **MI-4** **→** **MI-5** **→** **MI-6** **→** **MI-4m** simulates the propagation stage of the mononuclear ROP. This reaction profile resembles the profile of lactide ROP for mononuclear BHT-Mg complexes [20,42]. The relative free energies and enthalpies of the stationary points and transition states for the mononuclear mechanism of MeOEP polymerization are presented in Table 1, and molecular structures, energy parameters, and Cartesian coordinates are provided in the Supporting Information. The most stable stationary point for the mononuclear mechanism is **MI-6**, and the free energy of activation for MeOEP ROP can be estimated as 14 kcal/mol.

### 3.3. DFT Modeling of the Binuclear ROP Mechanism

To the best of our knowledge, only two publications have focused on DFT modeling of the binuclear coordination-insertion ROP mechanism [20,43]. In our recent work, [20] we demonstrated that polymerization of cyclic esters may be catalyzed by both "opened" and chelated binuclear BHT-Mg complexes. To estimate the formation pathways and relative stabilities of chelate binuclear complexes in ROP of cyclic phosphates, we performed DFT modeling of the initiation and propagation stages for the **Mg1**-catalyzed polymerization of MeOEP.

#### 3.3.1. Initiation Stage

**Scheme 4:**
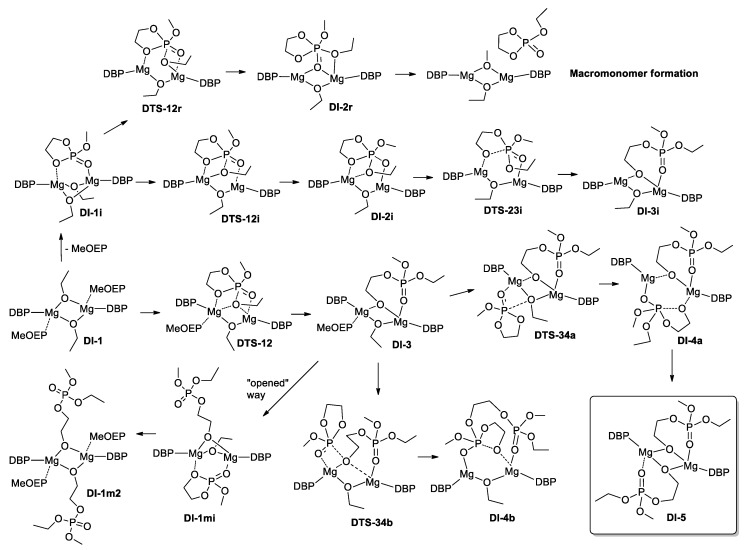
Key intermediates and transition states of the initiation stage of MeOEP ROP catalyzed by the binuclear DBP-Mg complexes.

**Figure 2:**
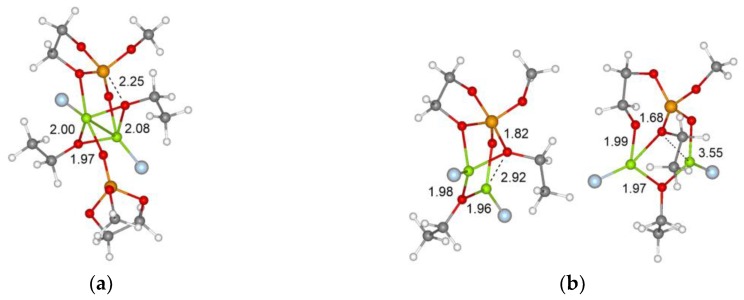
(**a**) Cooperative effect of di-Mg core in transition state **DTS-12**; (**b**) Geometries of the highly distorted transition state **DTS-12i** and intermediate **DI-2i** with a highly weakened exocyclic P–O bond (DBP fragments are omitted for clarity, DBP oxygen atoms are marked in blue).

We used the model binuclear complex [(DBP)Mg(μ-OEt)(MeOEP)]_2_ as a starting stationary point **DI-1** of the initiation stage. There are two reaction pathways at the beginning of the initiation process. Nucleophilic attack of ethoxy group at the phosphorus atom in MeOEP requires significant weakening of at least one Mg–OEt bond. Directly in the complex **DI-1**, apical nucleophilic addition of the OEt group to the phosphorus atom of MeOEP proceeds via transition state **DTS-12** (Δ*G***_DI-I_** = 13.5 kcal/mol), and this process is facilitated by the cooperative effect of the di-Mg core in the form of coordination of both phosphate and endocyclic oxygen atoms at the two magnesium atoms (Figure 2a). The hypothetic product **DI-2** is unstable and immediately transforms by ring-opening to low-energy complex **DI-3** (Δ*G*_DI-I_ = −14.7 kcal/mol). The alternative reaction pathway to **DI-3** proceeds via the loss of one of the MeOEP molecules with the formation of **DI-1i** (Scheme 4). This process is thermodynamically allowed (Δ*G*_DI-I_ = −1.3 kсal/mol). In **DI-1i**, the single MeOEP molecule is coordinated at both Mg atoms, facilitating the attack of the OEt group on the phosphorus atom. The free energy of the corresponding transition state **DTS-12i** (Δ*G*_DI-I_ = 12.5 kcal/mol) is lower than the Δ*G*_DI-I_ of **DTS-12**. The Mg_2_O_2_ core in **DTS-12i** is highly distorted: the maximum distance between one of the Mg atoms and the O atom of the ethoxy group d(Mg–O) is 2.91 Å (Figure 2b). In contrast to **DI-2**, the pentacoordinated intermediate **DI-2i** is a high-energy stationary point (Δ*G*_DI-I_ = 10.7 kcal/mol) with a weakened exocyclic P–O bond, *d*(P–O = 1.88 Å. **DI-2i** easily transforms into **DI-3i** (Δ*G*_DI-I_ = −6.1 kcal/mol), and the coordination of the MeOEP molecule results in **DI-3**.

When the bond between the ethoxy group and the magnesium atom coordinated to the phosphate oxygen atom is broken, the intermediate **DI-2r** is formed through the transition state **TS-12r**, in which the bridge position between the Mg atoms is occupied by the phosphate oxygen atom. The free energy of **DTS-12r** is 5 kcal/mol higher than that of **DTS-12i**, but **DI-2r** is more stable than **DI-2i**. Dissociation of **DI-2r** leads to ethyl-substituted cyclic phosphate that mimic the polymeryl-substituted cyclic ethylene phosphates (Scheme 4).

There are two possible pathways for the nucleophilic attack of the MeOEP phosphorus atom by the Mg-coordinated alkoxy groups in **DI-3**. The first is ethoxy group attack, and the second is an attack by the oxygen atom of the chelating OCH_2_CH_2_OP(O)OMe fragment. **DI-4a**, that is formed through the first pathway via the transition state **DTS-34a**, is unstable, and easily transforms into the extremely stable **DI-5**. The second pathway is characterized by the higher activation barrier and leads to **DI-4b**; for this molecule, steric factors block the ring-opening. The free energy of **DI-5** relative to **DI-1** is estimated as −21.1 kcal/mol. The formation of this highly stable doubly chelated intermediate concludes the initiation stage of MeOEP polymerization.

#### 3.3.2. Propagation Stage

**Scheme 5:**
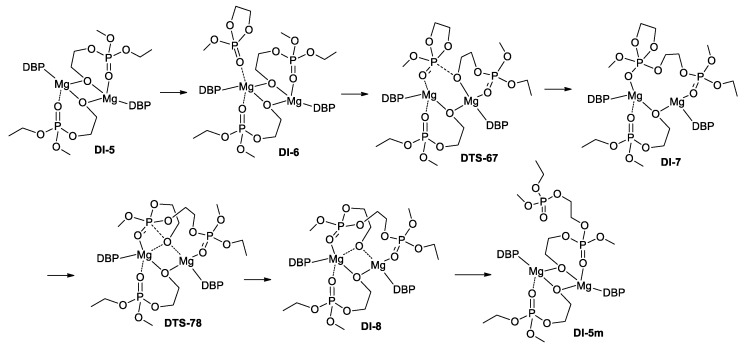
Key intermediates and transition states of the propagation stage of MeOEP ROP catalyzed by binuclear DBP-Mg complexes.

At the first step of the chain propagation stage, **DI-5** molecule coordinates one MeOEP molecule with the formation of **DI-6**. Then, via the insertion transition state **DTS-67**, the orthophosphate intermediate **DTS-7** forms. This intermediate transforms into the strained macrocyclic complex **DI-8** (activation barrier **DTS-78** is highest for the reaction profile of binuclear mechanism) and, finally, into the product **DI-5m** (Scheme 5) that is isostructural to **DI-5** and concludes the propagation stage. The relative free energies and enthalpies of the stationary points and transition states for the binuclear mechanism of MeOEP polymerization are presented in Table 2; the corresponding molecular structures, energy parameters, and Cartesian coordinates are provided in the Supporting Information.

The most stable stationary point for the binuclear mechanism is **DI-5**; thus, the free energy of activation for MeOEP ROP by the binuclear mechanism can be estimated as 25 kcal/mol.

### 3.4. Polymerization Experiments

**Figure 3:**
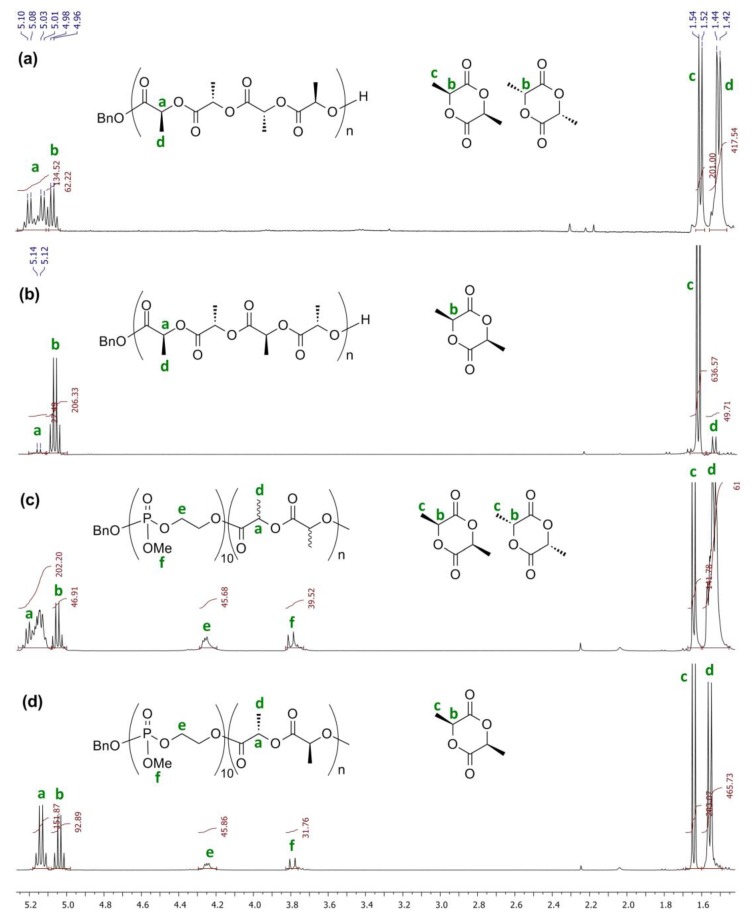
Fragments of ^1^H NMR spectra of PLAs obtained from *rac*-LA and *l*-LA in polymerization experiments (5 °C, 10 min, 1 M monomer solutions, LA/Mg ratio 100:1): (**a**) Homopolymerization of *rac*-LA; (**b**) Homopolymerization of *l*-LA; (**c**) Polymerization of *rac*-LA initiated by BnO(MeOEP)_10_-Mg-BHT; (**d**) Polymerization of *l*-LA initiated by BnO(MeOEP)_10_-Mg-BHT. Integrals refer to signals of the –C_6_H_5_ (5H) group of the benzyl initiator.

For experimental verification of the results of DFT modeling, we performed polymerization experiments using εCL, *rac*-LA, *l*-LA, and MeOEP as monomers, and **Mg1** complex (R′ = Bn) as a catalyst (Table 3) in a low-polarity solvent (CH_2_Cl_2_). We found that, at room temperature, εCL and *rac*-LA polymerize with comparable rates, and the ROP of *rac*-LA was slightly faster (Table 3, runs 1 and 2). In both cases, polymers with sharp MWD were formed, and the *M_n_* values determined by the analysis of polymer end-groups are in good agreement with the theoretical values. Thus, we confirmed our previous conclusion [20] concerning the living character of ε-caprolactone and *rac*-lactide ROP, and uniformity of the catalytic particles in these processes. At 5 °C, polymerization of *rac*-LA resulted in heterotactic PLA (Figure 3a).

*l*-LA in 1 M concentration at 5 °C demonstrated only moderate activity with the formation of isotactic (Figure 3b) PLA with narrow MWD (Table 3, run 3). Full monomer conversion was reached after 4 h. In contrast to cyclic esters, MeOEP demonstrated high activity (Table 3, run 4). At 5 °C, full conversion of monomer was reached after 1 min. In addition, near quantitative conversions after 10 min were registered in low-temperature MeOEP polymerization experiments (Table 3, runs 5 and 6).

The extremely high activity of MeOEP allowed us to propose the qualitative difference in the reaction mechanisms for cyclic esters and cyclic phosphates when BHT-Mg single-component dimeric catalyst **Mg1** were used. To confirm or disprove this assumption, we performed additional experiments. At the first stage of these experiments, we performed prepolymerization of MeOEP at 5 °C (monomer/Mg ratio was 10:1, full conversion of MeOEP was observed) with the subsequent introduction of *rac*-LA or *l*-LA solutions into the reaction mixtures containing active polyphosphate-Mg catalytic complex (Table 3, runs 7 and 8, respectively). In contrast to experiments 2 and 3, we found that the reactivities of *rac*-LA and *l*-LA were similar to each other with the formation of poly(MeOEP)-*b*-PLA copolymers (Figure 3c,d). It is noteworthy that the *l*-LA polymerization catalyzed by the MeOEP-derived BHT-Mg macroinitiator was several times faster than the ROP of *l*-LA catalyzed by **Mg1**. In addition, the polymers obtained from *rac*-LA in runs 2 and 8 had different stereoregularity: the product of the *rac*-LA polymerization initiated by polyphosphate-Mg-BHT (Figure 3c) had much lower degree of heterotacticity compared to the stereoregular PLA formed in **Mg1**-catalyzed homopolymerization (Figure 3a).

High dispersity of poly(MeOEP)-*b*-PLA copolymers may be explained by the simultaneous occurrence of LA ROP and transesterification in polyphosphate blocks. The similar reactions were detected by us earlier in MeOEP homopolymerization initiated by organocatalysts [16]. The presence of additional signals in ^31^P NMR spectra of poly(MeOEP)-*b*-PLA copolymers, in contrast with MeOEP homopolymers (see the Supporting Information, Figures S6, S7, and S3–S5, respectively), confirms this assumption. 

## 4. Discussion

**Figure 4:**
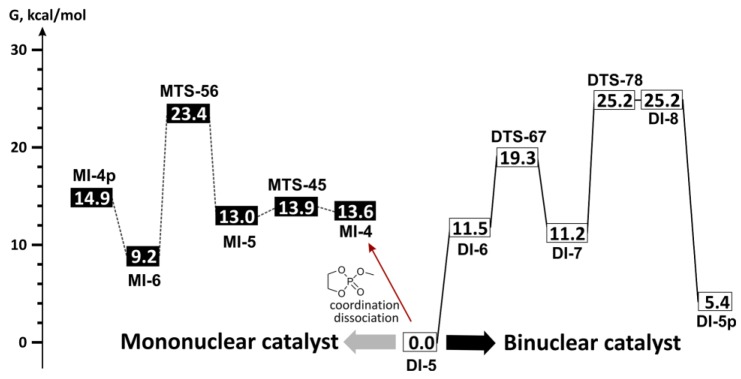
Energy profiles of BHT-Mg-catalyzed ROP of MeOEP for the mononuclear (---- dark gray, left) and binuclear (**—** black, right) reaction mechanisms.

The free energy of activation of the ROP of MeOEP catalyzed by mononuclear magnesium complexes is approximately 14 kcal/mol, and the activation barrier for the binuclear mechanism is 25.2 kcal/mol. To establish the preferred ROP mechanism for cyclic phosphates, we calculated the free energy change in the formation of **MI-4** by additional coordination of MeOEP and dissociation of the binuclear complex **DI-5**. Taking into account this difference (13.6 kcal/mol_Mg_), we compared the reaction profiles of the propagation stages for the mononuclear and binuclear mechanisms (Figure 4).

Figure 4 demonstrates that the mononuclear reaction pathway has lower activation energy. The dissociation of the binuclear complex at the initial stage of ROP is facilitated by a high concentration of MeOEP. The concentration of MeOEP decreases during the polymerization, but recombination of the mononuclear catalytic species to form structures such as **DI-5** is statistically improbable, and polymerization proceeds along the mononuclear path until the full conversion of the monomer. The results of MeOEP polymerization experiments confirm this mechanistic assumption, and the extremely high rate of MeOEP polymerization validates the mononuclear reaction mechanism with a low activation barrier of ca. 14 kcal/mol. An essential argument in favor of the assumption that MeOEP switches the ROP mechanism from binuclear to mononuclear pathway is the result for the lactide polymerization initiated by poly(MeOEP)-Mg(BHT). After prepolymerization of MeOEP in the presence of **Mg1**, *l*-lactide polymerization is almost an order of magnitude faster than the homopolymerization of *l*-LA. The acceleration of this reaction is in good agreement with the values of the activation barriers of the binuclear and mononuclear ROP mechanisms for *l*-LA (31.2 and 22.7 kcal/mol, respectively). Polymerization of racemic lactide, initiated by **Mg1**, leads to heterotactic PLA; however, after MeOEP prepolymerization, the reaction results in the formation of the copolymer containing the atactic PLA blocks.

Thus, the **Mg1**-catalyzed ROP of MeOEP essentially differs from the polymerization of cyclic esters, according to previously reported data [20], and the binuclear mechanism is preferred for the ROP of εCL and LA. The reactivity of the substrates is in the order of MeOEP >> *rac*-LA > εCL >> *l*-LA. This sequence is in good agreement with the free energies of activation (Table 3) that were found previously for cyclic esters [20] and that were calculated in this work for MeOEP. 

In our recent work [20], we demonstrated that the activity of dimeric catalyst **Mg1** in εCL and *rac*-LA polymerizations is noticeably lower than that of monomeric catalysts generated in situ by the activation of monomeric BHT-Mg alkyls with alcohols [19,20]. This activation can be performed in the presence of cyclic substrates to avoid the dimerization of labile mononuclear BHT-Mg-OR complexes. Under these conditions, the reaction of Mg alkyls with cyclic esters with the formation of different alkoxy initiators may complicate ROP. Prepolymerization of cyclic phosphate is an alternative method for the formation of highly active mononuclear catalytic species that may be applied in controlled synthesis of polyphosphate-containing block copolymers.

## 5. Conclusions

Here, we reported for the first time the results of DFT modeling of the ROP of cyclic phosphates. Binuclear heteroleptic aryloxy-alkoxy magnesium complexes were considered to be catalysts. We found that the mononuclear reaction mechanism with an extremely low activation barrier is preferable for the polymerization of methyl ethylene phosphate. Thus, ethylene phosphates may be used as “monomerization” agents for dimeric BHT-Mg single-component catalysts to form highly active species in the synthesis of ethylene phosphate copolymers that are prospective materials for biomedical applications.

## Figures and Tables

**Table 1:** Calculated Δ*G***_MI-1_** and Δ*H***_MI-1_** values for the key intermediates and transition states for the mononuclear mechanism of MeOEP polymerization.

**Molecule**	**MI-1**	**MTS-12**	**MI-2**	**MTS-23**	**MI-3**	**MI-4**	**MI-1m**
Δ*G***_MI-1_**, kcal/mol	0.0	6.6	−2.8	8.5	−3.7	−7.0	−2.3
Δ*H***_MI-1_**, kcal/mol	0.0	3.5	−6.0	3.6	−7.1	−8.9	−12.4
**Molecule**	**MTS-45**	**MI-5**	**MI-5m**	**MTS-56**	**MI-6**	**MI-6m**	**MI-4m**
Δ*G***_MI-1_**, kcal/mol	−6.8	−7.6	−0.2	2.7	−11.4	−1.7	−5.7
Δ*H***_MI-1_**, kcal/mol	−10.2	−9.9	−17.7	−2.9	−13.8	−17.6	−16.8

**Table 2:** Calculated ΔG**_DI-1_** and ΔH**_DI-1_** values for the key intermediates and transition states for the binuclear mechanism of MeOEP polymerization.

**Molecule**	**DI-1**	**DI-1i**	**DTS-12**	**DTS-12i**	**DTS-12r**	**DI-2i**	**DI-2r**
Δ*G***_MI-1_**, kcal/mol	0.0	−3.2	13.1	12.5	17.4	10.7	9.5
Δ*H***_MI-1_**, kcal/mol	0.0	13.4	9.4	23.8	28.6	21.5	22.8
**Molecule**	**DTS-23i**	**DI-3i**	**DI-3**	**DTS-34a**	**DTS-34b**	**DI-4a**	**DI-4b**
Δ*G***_MI-1_**, kcal/mol	10.7	−6.1	−14.7	3.8	8.4	2.7	6.5
Δ*H***_MI-1_**, kcal/mol	21.5	6.8	−13.1	0.9	5.1	−0.8	5.3
**Molecule**	**DI-5**	**DI-6**	**DTS-67**	**DI-7**	**DTS-78**	**DI-8**	**DI-5m**
Δ*G***_MI-1_**, kcal/mol	−21.1	−9.6	−1.8	−9.9	4.1	4.1	−16.6
Δ*H***_MI-1_**, kcal/mol	−19.2	−21.6	−15.5	−25.1	−13.4	−10.0	−24.1

**Table 3:** Polymerization experimental data and calculated Δ*G*^≠^ values for **Mg1**-catalyzed ROP of cyclic esters and MeOEP. Reaction conditions: 1 M solution of monomer in CH_2_Cl_2_, 1 mol % of **Mg1** catalyst.

Run	Monomer	Reac. time, ^(a)^ min	Reac. T, °C	Conv., % ^(b)^	*M_n_*^theo^ × 10^3 (c)^	*M_n_*^NMR^ × 10^3 (d)^	*M_n_*^SEC^ × 10^3^	*Ð_M_*	Δ*G*^≠^ kcal/mol
1	εCL	10	5	54	6.3	6.8	6.44 ^(e)^	1.18	21.7 ^(f)^
2	*rac*-LA	10	5	67	9.8	10.1	9.65 ^(e)^	1.24	21.6 ^(f)^
3a	*L*-LA	10	5	9	1.5	n.d. ^(g)^	n.d.	n.d.	31.2 ^(f)^
3b	*L*-LA	45	5	37	5.4	5.7	5.55 ^(e)^	1.21	29.4 ^(f)^
3c	*L*-LA	240	5	98	14.5	15.3	15.28 ^(e)^	1.20	29.4 ^(f)^
4	MeOEP	10	5	>99	13.9	13.7	9.8 ^(h)^	1.35	~14
5	MeOEP	10	−20	96	13.9	13.2	10.0 ^(h)^	1.32	~14
6	MeOEP	10	−50	81	11.2	11.4	8.8 ^(h)^	1.26	~14
7	MeOEP/*rac*-LA	10	5	>99/82	13.2	15.9	19.5 ^(h)^	1.62	~14/18.2 ^(i)^
8	MeOEP/*L*-LA	10	5	>99/62	10.3	12.5	15.7 ^(h)^	1.71	~14/22.7 ^(i)^

^(a)^ Reaction time. ^(b)^ Monomer conversion. ^(c)^ *M_n_*^theo^ = *MW*_M_ × [M]_0_/[I]_0_ × Conversion + *MW*_I_, *MW*_M_—molecular weights of monomers, *MW*_I_—molecular weight of BnOH, [M]_0_/[I]_0_—monomer to initiator initial ratio. ^(d)^ Determined by the analysis of the NMR spectra of the reaction mixtures, see the SI for details. ^(e)^ Determined by SEC in THF vs polystyrene standards and corrected by the factors of 0.56 (εCL) and 0.58 (*rac*-LA). ^(f)^ Previously reported data for the binuclear mechanism [20]. ^(g)^ Not determined. ^(h)^ Determined by SEC in DMF vs PEG standards. ^(i)^ Previously reported data for the mononuclear mechanism [20].

## Supplementary Materials

The following are available online at http://www.mdpi.com/2073-4360/10/10/1105/s1, Figure S1: Molecular structure of MeOEP and geometry parameters analyzed in DFT calculations, Table S1: Structural parameters of the MeOEP molecule calculated by DFT using different functional/basis sets, and deviations ΔX from the X-ray data, Table S2: Efficiency rating for the functional/basis sets used for the MeOEP molecule calculations, part S1.2: a list of molecular structure plots, energy values, and Cartesian coordinates for the calculated stationary points and transition states, part S2: polymerization method parameters, Figures S2–S7: NMR spectra of the polymers.
