# Supplementary material for: Mechanistic Insights of BHT-Mg-Catalyzed Ethylene Phosphate’s Coordination Ring-Opening Polymerization: DFT Modeling and Experimental Data
Source: Polymers (Basel). 2018 Oct 6;10(10):1105. doi: 10.3390/polym10101105 (PMC6403938; doi:10.3390/polym10101105)
Supplement: Supplementary file 1 [file polymers-10-01105-s001.pdf]

Mechanistic insights of BHT-Mg-catalyzed ethylene phosphate's coordination ring-opening polymerization: DFT modeling and experimental data

Ilya Nifant'ev,[1,2]* Andrey Shlyakhtin,[1] Maxin Kosarev,[1] Stanislav Karchevsky[3] and Pavel Ivchenko[1]

[1] M.V. Lomonosov Moscow State University, Chemistry Department, 1-3 Leninskie Gory, Moscow, Russia 119991; inif@org.chem.msu.ru (I.N.), phpasha1@yandex.ru (P.I.), shlyahtinav@mail.ru (A.S.) komrad.kosarev.maksim@gmail.com (M.K.)

[2] A.V. Topchiev Institute of Petrochemical Synthesis RAS, 29 Leninsky Pr., Moscow, Russia 119991; ilnif@yahoo.com

[3] Joint-stock company "Institute of petroleum refining and petrochemistry", 12 Iniciativnaya Str., Ufa, Republic of Bashkortostan, Russia 450065; st_karchevsky@mail.ru

* Correspondence: ilnif@yahoo.com; Tel.: +7-495-939-4098

SUPPORTING INFORMATION

S1. DFT calculations 2

S1.1. Rationale for the functional and basis set used for DFT calculations 2

S1.2. Molecular structures, energies and cartesian coordinates, mononuclear mechanism 5

S1.3. Molecular structures, energies and cartesian coordinates, binuclear mechanism 38

S2. Polymerization and polymer spectra 108

S2.1. Polymerization experiments 108

S2.2. NMR spectra of polymers 109

References 114

S1. DFT calculations

S1.1. Rationale for the functional and basis set used for DFT calculations

To confirm the applicability of the B3PW91/DGTZVP functional/basis combination for the modeling of reactions with cyclic ethylene phosphate, we calculated the optimized molecular structure of MeOEP using a number of functional/basis pairs implemented in the Gaussian 09 program package [1]. The calculated values of the bond lengths and endocyclic angle O–P–O for MeOEP and relative deviations of these parameters from the X-ray data (Figure S1) [2] ΔX (%) = $|X_{X\text{-}Ray} - X_{DFT}| \cdot 100$ are provided in Table S1. As a criterion for the rating, we used the results of the multiplication of the total relative deviations $\Sigma\Delta$ and the computation time (Table S2). We found that B3PW91/DGTZVP set that was used in the earlier DFT modeling of ε-caprolactone and rac-lactide ROP [3] is suitable for the modeling of cyclic phosphate polymerization.

Clearly, our calculations performed for the gas phase are rough approximations. However, the complexity of molecular models makes it impossible to perform calculations using standard algorithms that take into account the influence of the solvent (IEFPCM, etc.) but dramatically increase the calculation time.

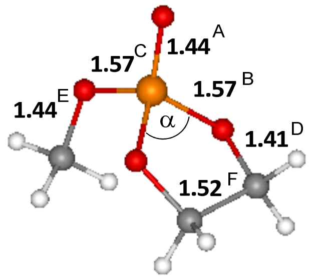

Figure S1. Molecular structure of MeOEP and geometry parameters analyzed in DFT calculations

Table S1 Structural parameters of the MeOEP molecule calculated by DFT using different functional/basis sets, and deviations Δ_X from the X-Ray data [2].

Functional	Basis	A	Δ_A, %	B	Δ_B, %	C	Δ_C, %	D	Δ_D, %	E	Δ_E, %	F	Δ_F, %	α	Δ_α, %
X-Ray data		1.440	Δ_A, %	1.570	0	1.570	0	1.410	0	1.440	0	1.520	0	99.10	0
B3LYP	6-31g	1.574	9.31	1.759	12.04	1.706	8.66	1.480	4.96	1.474	2.36	1.536	1.05	92.99	6.17
B3LYP	6-31g(d)	1.469	2.01	1.638	4.33	1.600	1.91	1.439	2.06	1.436	0.28	1.537	1.12	96.00	3.13
B3LYP	6-31g(d,p)	1.469	2.01	1.637	4.27	1.600	1.91	1.439	2.06	1.436	0.28	1.537	1.12	96.00	3.13
B3LYP	6-311g	1.554	7.92	1.746	11.21	1.681	7.07	1.477	4.75	1.468	1.94	1.535	0.99	92.66	6.50
B3LYP	6-311g(d)	1.463	1.60	1.634	4.08	1.593	1.46	1.440	2.13	1.435	0.35	1.536	1.05	95.68	3.45
B3LYP	6-311g(d,p)	1.463	1.60	1.634	4.08	1.593	1.46	1.440	2.13	1.437	0.21	1.537	1.12	95.69	3.44
B3LYP	DGTZVP	1.469	2.01	1.636	4.20	1.597	1.72	1.444	2.41	1.443	0.21	1.539	1.25	96.04	3.09
B3LYP	LanL2DZ	1.568	8.89	1.747	11.27	1.689	7.58	1.479	4.89	1.472	2.22	1.553	2.17	92.99	6.17
B3PW91	6-31g	1.571	9.10	1.755	11.78	1.701	8.34	1.471	4.33	1.466	1.81	1.531	0.72	92.98	6.18
B3PW91	6-31g(d)	1.467	1.88	1.633	4.01	1.596	1.66	1.432	1.56	1.429	0.76	1.531	0.72	96.05	3.08
B3PW91	6-31g(d,p)	1.467	1.88	1.634	4.08	1.596	1.66	1.432	1.56	1.429	0.76	1.531	0.72	96.05	3.08
B3PW91	6-311g	1.549	7.57	1.742	10.96	1.676	6.75	1.468	4.11	1.459	1.32	1.529	0.59	92.73	6.43
B3PW91	6-311g(d)	1.460	1.39	1.630	3.82	1.589	1.21	1.432	1.56	1.428	0.83	1.530	0.66	95.79	3.34
B3PW91	6-311g(d,p)	1.460	1.39	1.629	3.76	1.589	1.21	1.433	1.63	1.430	0.69	1.530	0.66	95.80	3.33
B3PW91	DGTZVP	1.466	1.81	1.630	3.82	1.593	1.46	1.436	1.84	1.435	0.35	1.532	0.79	96.09	3.04
B3PW91	LanL2DZ	1.567	8.82	1.745	11.15	1.688	7.52	1.472	4.40	1.465	-1.74	1.547	1.78	92.96	6.20
BPW86	6-31g(d)	1.483	2.99	1.657	5.54	1.618	3.06	1.448	2.70	1.445	0.35	1.539	1.25	96.35	2.77
BPW86	6-31g(d,p)	1.483	2.99	1.657	5.54	1.618	3.06	1.448	2.70	1.446	0.42	1.538	1.18	96.35	2.77
BPW86	6-311g(d)	1.476	2.50	1.654	5.35	1.610	2.55	1.449	2.77	1.445	0.35	1.537	1.12	96.01	3.12
BPW86	DGTZVP	1.482	2.92	1.622	3.31	1.614	2.80	1.453	3.05	1.452	0.83	1.540	1.32	96.32	2.81
CAM-B3LYP	6-31g(d)	1.462	1.53	1.625	3.50	1.589	1.21	1.431	1.49	1.427	0.90	1.531	0.72	95.85	3.28
CAM-B3LYP	6-31g(d,p)	1.462	1.53	1.624	3.44	1.589	1.21	1.431	1.49	1.428	0.83	1.530	0.66	95.85	3.28
CAM-B3LYP	6-311g(d)	1.455	1.04	1.623	3.38	1.582	0.76	1.431	1.49	1.426	0.97	1.530	0.66	95.50	3.63
CAM-B3LYP	DGTZVP	1.461	1.46	1.624	3.44	1.586	1.02	1.435	1.77	1.433	0.49	1.533	0.86	95.89	3.24
LSDA	6-31g(d,p)	1.469	2.01	1.631	3.89	1.594	1.53	1.422	0.85	1.417	1.60	1.516	0.26	96.01	3.12
MPW1PW91	6-31g(d)	1.464	1.67	1.628	3.69	1.592	1.40	1.429	1.35	1.425	1.04	1.529	0.59	96.03	3.10
MPW1PW91	6-31g(d,p)	1.464	1.67	1.629	3.76	1.592	1.40	1.429	1.35	1.426	0.97	1.529	0.59	96.03	3.10
MPW1PW91	6-311g(d)	1.457	1.18	1.625	3.50	1.584	0.89	1.428	1.28	1.424	1.11	1.527	0.46	95.79	3.34
MPW1PW91	DGTZVP	1.462	1.53	1.625	3.50	1.588	1.15	1.432	1.56	1.431	0.62	1.530	0.66	96.08	3.05
PBEPBE	6-31g(d)	1.483	2.99	1.656	5.48	1.618	3.06	1.445	2.48	1.442	0.14	1.537	1.12	96.41	2.71
PBEPBE	6-31g(d,p)	1.483	2.99	1.657	5.54	1.618	3.06	1.445	2.48	1.443	0.21	1.536	1.05	96.40	2.72
PBEPBE	6-311g(d)	1.477	2.57	1.653	5.29	1.610	2.55	1.446	2.55	1.441	0.07	1.534	0.92	96.09	3.04
PBEPBE	DGTZVP	1.482	2.92	1.655	5.41	1.615	2.87	1.450	2.84	1.448	0.56	1.538	1.18	96.40	2.72
WB97XD	6-31g(d,p)	1.462	1.53	1.626	3.57	1.592	1.40	1.428	1.28	1.426	0.97	1.532	0.79	96.09	3.04

Table S2 Efficiency rating for the functional/basis sets used for the MeOEP molecule calculations

Functional	Basis	Time	Deviation $\Sigma\Delta$ [a)]	T·ΣΔ	Range
B3LYP	6-31g	238	44.55	10603	12
B3LYP	6-31g(d)	656	14.84	9733	7
B3LYP	6-31g(d,p)	851	14.77	12572	21
B3LYP	6-311g	477	40.38	19261	34
B3LYP	6-311g(d)	1042	14.12	14710	27
B3LYP	6-311g(d,p)	1336	14.03	18749	33
B3LYP	DGTZVP	727	14.89	10829	14
B3LYP	LanL2DZ	236	43.19	10194	10
B3PW91	6-31g	234	42.26	9888	9
B3PW91	6-31g(d)	641	13.67	8762	3
B3PW91	6-31g(d,p)	826	13.73	11343	16
B3PW91	6-311g	471	37.73	17771	32
B3PW91	6-311g(d)	1054	12.81	13504	23
B3PW91	6-311g(d,p)	1354	12.67	17156	30
B3PW91	DGTZVP	730	13.11	9570	6
B3PW91	LanL2DZ	236	38.11	8995	5
BPW86	6-31g(d)	592	18.65	11042	15
BPW86	6-31g(d,p)	745	18.66	13899	24
BPW86	6-311g(d)	915	17.75	16239	29
BPW86	DGTZVP	669	17.04	11397	17
CAM-B3LYP	6-31g(d)	856	12.64	10817	13
CAM-B3LYP	6-31g(d,p)	1122	12.44	13955	25
CAM-B3LYP	6-311g(d)	1470	11.93	17543	31
CAM-B3LYP	DGTZVP	1007	12.27	12356	19
LSDA	6-31g(d,p)	370	13.26	4905	1
MPW1PW91	6-31g(d)	646	12.84	8296	2
MPW1PW91	6-31g(d,p)	819	12.84	10512	11
MPW1PW91	6-311g(d)	1051	11.76	12364	20
MPW1PW91	DGTZVP	734	12.07	8858	4
PBEPBE	6-31g(d)	549	17.98	9868	8
PBEPBE	6-31g(d,p)	745	18.05	13449	20
PBEPBE	6-311g(d)	916	16.98	15558	28
PBEPBE	DGTZVP	664	18.50	12283	18
WB97XD	6-31g(d,p)	1144	12.57	14382	26

S1.2. Molecular structures, energies and cartesian coordinates for mononuclear mechanism

MI-1_endo

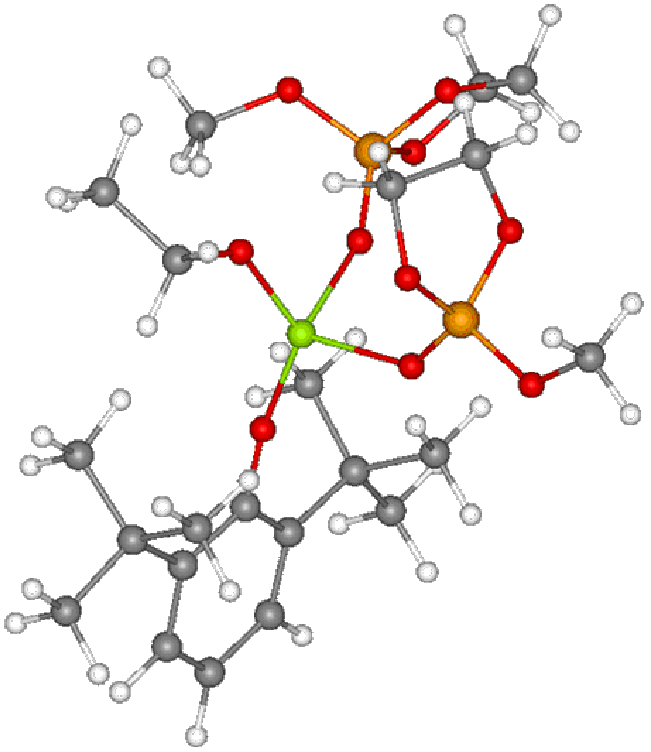

Zero-point vibrational energy	1639202.4 (Joules/Mol)
	391.77877 (Kcal/Mol)
Zero-point correction=	0.624339 (Hartree/Particle)
Thermal correction to Energy=	0.666862
Thermal correction to Enthalpy=	0.667806
Thermal correction to Gibbs Free Energy=	0.544211
Sum of electronic and zero-point Energies=	-2496.477872
Sum of electronic and thermal Energies=	-2496.435350
Sum of electronic and thermal Enthalpies=	-2496.434406
Sum of electronic and thermal Free Energies=	-2496.558000

cartesian

12	0.31897891	-0.36698934	-0.23176536	1	-0.15942112	2.78511071	2.59413481
8	1.21027887	-1.28258932	-1.64466524	1	-0.44542113	1.16211069	1.94253469
8	-1.45792115	0.16491066	0.00173464	1	-0.10942110	-2.77738929	-2.23686528
6	0.93297887	-2.44438934	-2.37146521	8	1.06997883	-1.24288940	1.47293472
1	1.55657887	-3.28908944	-2.01646519	15	2.33447886	-1.95838928	1.77233458
6	1.18927884	-2.26758933	-3.86756516	8	3.66187906	-1.04538941	1.68703461
6	-2.71292090	0.36491066	0.38123465	8	2.80927896	-3.11948943	0.77323467

6	-3.73152089	-0.58588934	0.04803464	6	4.56017876	-1.53788936	0.66223466
6	-5.04042149	-0.34608933	0.47643465	6	3.74227905	-2.52698946	-0.17356536
1	-5.82442141	-1.05678940	0.23703465	1	5.40737867	-2.01428938	1.16423464
6	-5.38372135	0.78601068	1.20743465	1	4.89957857	-0.68208933	0.07833464
6	-4.39532137	1.71571064	1.51173472	1	3.15317893	-2.02558947	-0.95106536
1	-4.68042135	2.59901071	2.07393456	1	4.34537888	-3.33718944	-0.58296537
6	-3.06582093	1.54401064	1.11593461	8	2.23447895	-2.59668946	3.21213460
6	-3.41502094	-1.84928942	-0.77276534	6	3.32567906	-3.34958935	3.77583480
6	-2.01992106	2.61901069	1.46373463	1	3.54777908	-4.21918917	3.15373468
6	-2.43972111	-2.76088929	-0.00076535	1	4.20857906	-2.71438932	3.88233471
1	-1.52432120	-2.23948932	0.28313464	1	2.98517895	-3.67318940	4.75803471
1	-2.16582108	-3.63648939	-0.60256535	8	1.54927886	1.28391063	-0.24156535
1	-2.90582108	-3.11848927	0.92413461	15	2.60097909	2.16661072	-0.79446536
6	-2.83732104	-1.45928931	-2.14856529	8	4.11397886	1.91961062	-0.29406536
1	-1.92552125	-0.86828929	-2.05336523	8	2.45587897	3.68991065	-0.29756537
1	-3.56662107	-0.86168933	-2.70726538	6	4.54307890	3.00601053	0.55483460
1	-2.61362100	-2.35578942	-2.74036527	6	3.73827887	4.21851063	0.08633465
6	-4.66812134	-2.69808936	-1.05396533	1	5.61737871	3.13151073	0.41713464
1	-5.42012119	-2.14688945	-1.62876534	1	4.32517862	2.74491072	1.59393454
1	-5.13762140	-3.06498933	-0.13496536	1	3.57387900	4.94951057	0.87823462
1	-4.38282108	-3.57548928	-1.64586544	1	4.19667864	4.70381069	-0.78036535
6	-2.62882113	3.81311059	2.21933460	8	2.73627901	2.15111065	-2.36316538
1	-3.06262112	3.51921058	3.18133473	6	1.69967890	1.58291066	-3.22036529
1	-3.40132093	4.32461071	1.63493466	1	1.46397889	0.56691068	-2.88976526
1	-1.83992112	4.54521084	2.42833471	1	0.81927890	2.22791052	-3.20066524
6	-1.40152109	3.18801069	0.17043464	1	2.13577890	1.57621062	-4.21806526
1	-0.96112108	2.39701056	-0.43646538	1	1.01327884	-3.20258927	-4.41386509
1	-0.62262106	3.92401052	0.40623465	1	0.52627885	-1.50218940	-4.28616524
1	-2.17252111	3.68771052	-0.42716539	1	2.22527885	-1.95618939	-4.04696512
6	-0.93022108	2.03241062	2.38343477	1	-6.41002131	0.94501066	1.53013468
1	-1.36792111	1.71971059	3.33803463				

MI-1_exo

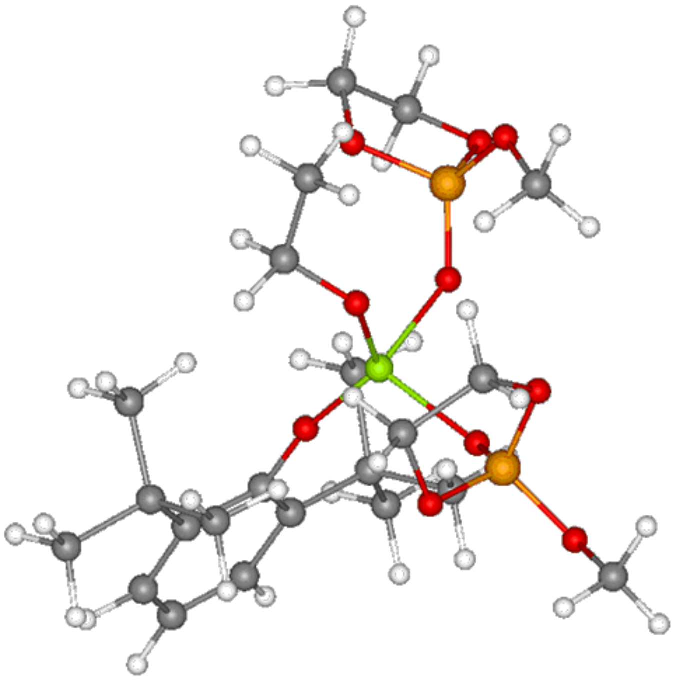

Zero-point vibrational energy	1638160.6 (Joules/Mol)
	391.52977 (Kcal/Mol)
Zero-point correction=	0.623942 (Hartree/Particle)
Thermal correction to Energy=	0.666703
Thermal correction to Enthalpy=	0.667647
Thermal correction to Gibbs Free Energy=	0.543208
Sum of electronic and zero-point Energies=	-2496.480479
Sum of electronic and thermal Energies=	-2496.437719
Sum of electronic and thermal Enthalpies=	-2496.436775
Sum of electronic and thermal Free Energies=	-2496.561213

cartesian

12	0.61740255	0.17141742	0.16157193	1	-1.30479741	1.32141745	3.73557210
8	1.97870255	0.01081741	-1.17392814	1	-0.86489737	0.18811741	2.44897199
8	-1.24289739	-0.05588260	0.07257193	6	3.28400254	0.73591739	-3.09402800
6	1.97560263	0.14851740	-2.56452799	8	1.49490261	-0.99968261	1.59577191
1	1.14980257	0.79141742	-2.91522789	15	2.53400254	-2.05778265	1.54237187
1	1.81180263	-0.82768261	-3.06222796	8	4.00910282	-1.57008255	1.12457192
6	-2.49749756	-0.47388262	0.17457193	8	2.36990237	-3.22068262	0.45137194
6	-3.02579761	-1.42018259	-0.76382810	6	4.43480253	-2.19108272	-0.11222808
6	-4.34529734	-1.85518253	-0.60912806	6	3.20340252	-2.90098262	-0.69282806
1	-4.75969744	-2.57748270	-1.30472803	1	5.24590254	-2.88268256	0.13177192
6	-5.16269732	-1.39028251	0.41517192	1	4.79240227	-1.40458250	-0.77622807
6	-4.65569735	-0.44968259	1.30527186	1	2.64110255	-2.23288274	-1.34672809

1	-5.30979776	-0.08498260	2.09047198	1	3.44850254	-3.84148264	-1.18622804
6	-3.34659743	0.03191741	1.21347189	8	2.61410236	-2.75338268	2.95797205
6	-2.18149757	-1.95198262	-1.93662798	6	3.54550242	-3.82268262	3.21027207
6	-2.85389757	1.09531748	2.21207190	1	3.34680247	-4.66408253	2.54297209
6	-0.96769738	-2.74448276	-1.41382813	1	4.57180262	-3.46718264	3.08917212
1	-0.33879742	-2.13978267	-0.76012808	1	3.37930250	-4.12248278	4.24377203
1	-0.35119745	-3.10078263	-2.24932790	8	1.04790258	2.03171730	0.89767194
1	-1.29819739	-3.61648273	-0.83902812	15	1.60740256	3.27651739	0.30687195
6	-1.72569740	-0.78298259	-2.83272791	8	1.17740262	4.65801716	1.00037193
1	-1.16219735	-0.04118259	-2.26632786	8	1.06320262	3.59051728	-1.17422807
1	-2.59599757	-0.28248259	-3.27202797	6	0.23190257	5.35401726	0.15877193
1	-1.09559739	-1.14838254	-3.65332794	6	0.60440254	4.95481730	-1.26962805
6	-2.96929741	-2.91578269	-2.84172797	1	0.34820256	6.42151737	0.34637195
1	-3.84809732	-2.43918276	-3.28882790	1	-0.77799737	5.03231716	0.42517191
1	-3.29869747	-3.81328273	-2.30702806	1	-0.24739745	4.97201729	-1.94962800
1	-2.32319760	-3.24528265	-3.66382790	1	1.41660261	5.56841755	-1.66952813
6	-3.94919729	1.52651751	3.20407200	8	3.18310237	3.33451724	0.31357193
1	-4.29589748	0.69521743	3.82727194	6	3.97180247	2.13351727	0.55597192
1	-4.81779766	1.96471751	2.70027208	1	3.93100238	1.88961744	1.61837196
1	-3.54259753	2.28911734	3.87847209	1	3.59490252	1.30451751	-0.04792808
6	-2.42649746	2.36861730	1.45257187	1	4.98750257	2.40101743	0.26737192
1	-1.65039742	2.14671731	0.71997190	1	3.27290249	0.80491740	-4.18892813
1	-2.04479742	3.12081742	2.15557194	1	3.44890237	1.74171746	-2.69252801
1	-3.28709745	2.79721737	0.92557192	1	4.13880253	0.11251741	-2.80412793
6	-1.69089746	0.54651743	3.06167197	1	-6.18459749	-1.74908257	0.51297194
1	-2.03389764	-0.29438260	3.67447209				

MI-1_front

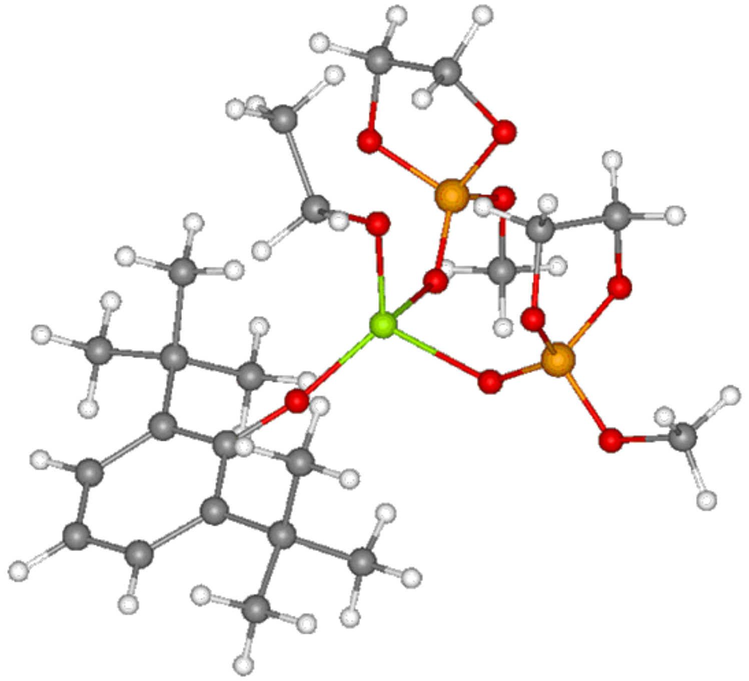

Zero-point vibrational energy	1638508.7 (Joules/Mol)
	391.61299 (Kcal/Mol)
Zero-point correction=	0.624075 (Hartree/Particle)
Thermal correction to Energy=	0.666802
Thermal correction to Enthalpy=	0.667746
Thermal correction to Gibbs Free Energy=	0.543363
Sum of electronic and zero-point Energies=	-2496.482337
Sum of electronic and thermal Energies=	-2496.439610
Sum of electronic and thermal Enthalpies=	-2496.438666
Sum of electronic and thermal Free Energies=	-2496.563049

cartesian

12	-0.52917743	0.00792127	0.16738404	1	1.68022275	-3.40807867	1.84568405
8	-1.43587732	-0.24947873	-1.51161599	1	1.31212258	-1.66827869	1.84758401
8	1.31352258	0.19312127	0.42098403	6	-1.15727735	-1.01647878	-3.81221604
6	-0.84607744	0.03982127	-2.75321603	8	-1.58137751	1.51832128	1.07628405
1	0.25112259	0.12962127	-2.67591596	15	-2.80577731	2.28942132	0.75298405
1	-1.19337738	1.01952136	-3.13511610	8	-4.18007755	1.45572126	0.86318403
6	2.62882257	0.31722128	0.31058404	8	-2.99417734	2.81842136	-0.74931592
6	3.22912264	1.61592126	0.22348404	6	-4.85557747	1.40142131	-0.41561592
6	4.61802244	1.70382130	0.09138405	6	-3.82557726	1.85512125	-1.45501590
1	5.09242249	2.67682123	0.01758404	1	-5.72247744	2.06642127	-0.36381596
6	5.43122244	0.57612127	0.05378404	1	-5.18217754	0.37382126	-0.57601595

6	4.84572268	-0.68057877	0.16128404	1	-3.17967725	1.02852130	-1.77421594
1	5.49522257	-1.54957867	0.14188404	1	-4.27437735	2.37472129	-2.30151606
6	3.46412277	-0.84707874	0.29348403	8	-2.88707733	3.54532123	1.70528400
6	2.38442278	2.90202141	0.28738403	6	-3.99377728	4.46532106	1.63928401
6	2.87322259	-2.26267862	0.42378405	1	-4.04727745	4.92462111	0.64978403
6	1.62362266	2.97142124	1.62698400	1	-4.92777729	3.95122123	1.87888408
1	0.98022264	2.10472131	1.77468407	1	-3.79027724	5.22712135	2.39018393
1	1.00012255	3.87362123	1.66828406	8	-1.37697744	-1.48107874	1.29268408
1	2.33322263	3.01402140	2.46118402	15	-2.24707747	-2.64217877	0.95518404
6	1.40642262	2.96712136	-0.90241599	8	-1.56587744	-3.68957877	-0.05011596
1	0.74412256	2.10162139	-0.93481588	8	-3.60367727	-2.38427877	0.14868404
1	1.95782256	2.99492121	-1.84881604	6	-2.45377731	-3.99117875	-1.14921594
1	0.78622264	3.87052131	-0.84481597	6	-3.41227722	-2.80387878	-1.23121595
6	3.24492264	4.17582130	0.20738405	1	-2.96717739	-4.93137884	-0.92791593
1	3.79742289	4.24922132	-0.73571593	1	-1.84497738	-4.09547853	-2.04691601
1	3.96132278	4.24282122	1.03308403	1	-2.96707726	-1.96287870	-1.77031589
1	2.59202266	5.05412149	0.27138406	1	-4.39197731	-3.07617879	-1.62511599
6	3.95622253	-3.35647869	0.42508405	8	-2.74217725	-3.39807868	2.25328398
1	4.65722275	-3.24587870	1.25948405	6	-2.00097752	-3.32107878	3.49508405
1	4.53122282	-3.37537861	-0.50691593	1	-1.17677736	-4.03607893	3.47078395
1	3.47662258	-4.33687878	0.52958405	1	-1.62157750	-2.31087875	3.65028405
6	1.94812274	-2.57297873	-0.77001595	1	-2.70867729	-3.58957863	4.27788401
1	1.14782262	-1.83897865	-0.87331593	1	-0.69327736	-0.76147878	-4.77271605
1	1.48992252	-3.56327868	-0.65651596	1	-0.77367741	-1.99517870	-3.50261617
1	2.52112269	-2.56827879	-1.70401597	1	-2.23897743	-1.10307872	-3.97521615
6	2.11282277	-2.40247869	1.75848401	1	6.50922251	0.67612123	-0.04971595
1	2.79912257	-2.25717878	2.60038400				

MTS-12_endo

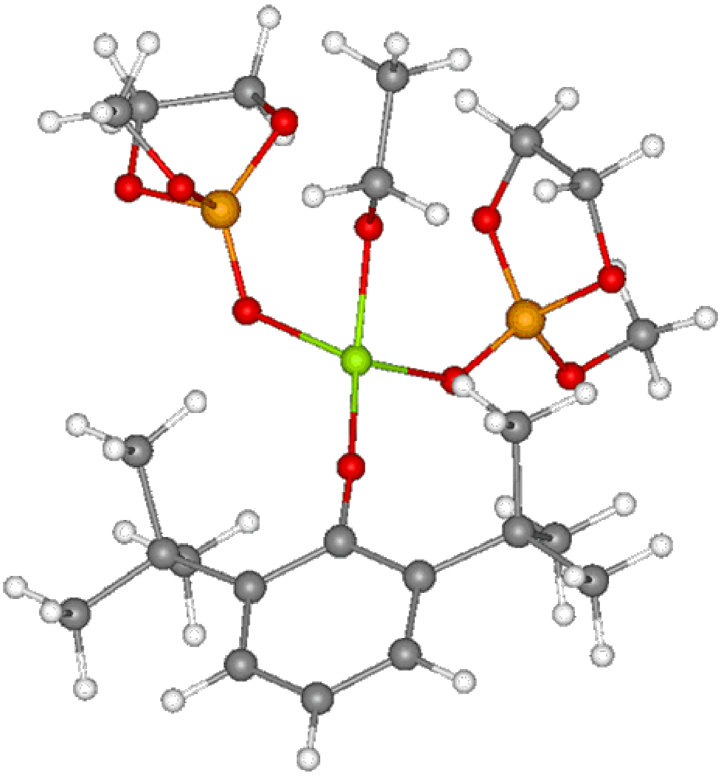

Zero-point vibrational energy	1638572.4 (Joules/Mol)
	391.62820 (Kcal/Mol)
Zero-point correction=	0.624099 (Hartree/Particle)
Thermal correction to Energy=	0.665768
Thermal correction to Enthalpy=	0.666712
Thermal correction to Gibbs Free Energy=	0.546409
Sum of electronic and zero-point Energies=	-2496.471257
Sum of electronic and thermal Energies=	-2496.429588
Sum of electronic and thermal Enthalpies=	-2496.428643
Sum of electronic and thermal Free Energies=	-2496.548947

cartesian

12	-0.25081635	0.02683736	0.32960403	1	1.35618365	-2.64716268	3.08470392
8	-1.52621639	-0.16206264	-1.12849593	1	1.32848358	-1.01256263	2.40320396
8	1.60698366	0.03993736	0.30200404	1	-0.76461637	-1.26256263	-2.71009612
6	-1.30801642	-0.32546264	-2.50649595	8	-0.93981636	1.63263738	1.38370407
1	-0.66601634	0.48403734	-2.89309597	15	-2.08391643	2.56693721	1.25010407
6	-2.60671616	-0.33836266	-3.31109595	8	-3.52091622	1.88633740	1.02140403
6	2.92938375	-0.05596264	0.27830404	8	-2.09711623	3.53363729	-0.03069597
6	3.71418357	1.00333738	-0.28129596	6	-4.01401615	2.13943720	-0.31709599

6	5.10478354	0.86433738	-0.30759597	6	-2.90841627	2.91223717	-1.05859590
1	5.71788359	1.65333736	-0.73089600	1	-4.93581629	2.71783733	-0.21539597
6	5.74398327	-0.26256266	0.19870403	1	-4.20431662	1.17563736	-0.78809601
6	4.97708368	-1.28026259	0.75610399	1	-2.27751637	2.23343730	-1.63349593
1	5.49148369	-2.14846277	1.15510404	1	-3.29831624	3.71193719	-1.68799591
6	3.58198380	-1.21186256	0.81560403	8	-2.15451622	3.48263717	2.53120399
6	3.05798364	2.28263736	-0.83249599	6	-3.16901636	4.49723721	2.67000389
6	2.78248382	-2.36256266	1.45330405	1	-3.06481624	5.24543715	1.88170409
6	2.28848362	3.00673723	0.29120404	1	-4.16291618	4.04433727	2.64380383
1	1.54288363	2.36073732	0.75470400	1	-2.99721622	4.95523739	3.64260387
1	1.78288352	3.89843726	-0.10229598	8	-1.39131641	-1.43046260	1.15760410
1	2.98198366	3.33193731	1.07470405	15	-2.39631629	-2.05166268	0.23770402
6	2.11768365	1.94313741	-2.00629616	8	-3.77421618	-1.29346263	-0.17349596
1	1.35918367	1.21573734	-1.71469593	8	-3.13701630	-3.23096275	1.11110401
1	2.68598366	1.51283741	-2.83849597	6	-4.83851671	-1.66246259	0.72650403
1	1.61558366	2.84823728	-2.37229609	6	-4.55781651	-3.11196280	1.11210406
6	4.08898354	3.28703737	-1.37719595	1	-5.77991629	-1.53666258	0.18980403
1	4.66908360	2.87603736	-2.21059608	1	-4.81161642	-0.99506259	1.59270406
1	4.78738356	3.62413716	-0.60399598	1	-4.92751646	-3.35836267	2.10970402
1	3.56368375	4.17363739	-1.75119591	1	-4.98431635	-3.81336284	0.38550401
6	3.68998384	-3.48646283	1.98460412	8	-1.77681637	-2.85396266	-0.98499602
1	4.37608337	-3.13356280	2.76200390	6	-2.47001624	-3.95116282	-1.59889591
1	4.28108358	-3.95426273	1.18980408	1	-2.62281632	-4.75726271	-0.87779599
1	3.06668377	-4.27006292	2.43070388	1	-3.42651629	-3.62086272	-2.01459599
6	1.85568368	-3.01546264	0.40800405	1	-1.82461643	-4.29146290	-2.40779614
1	1.18638361	-2.29286265	-0.05919598	1	-2.40281630	-0.49466267	-4.37719584
1	1.24358356	-3.79876280	0.87140399	1	-3.27191615	-1.13626266	-2.96619606
1	2.45108366	-3.47446275	-0.38939601	1	-3.14931631	0.60893738	-3.21059608
6	1.97858369	-1.84756267	2.66520405	1	6.82798338	-0.34396264	0.16480403
1	2.66058373	-1.49966264	3.44910407				

MTS-12_exo

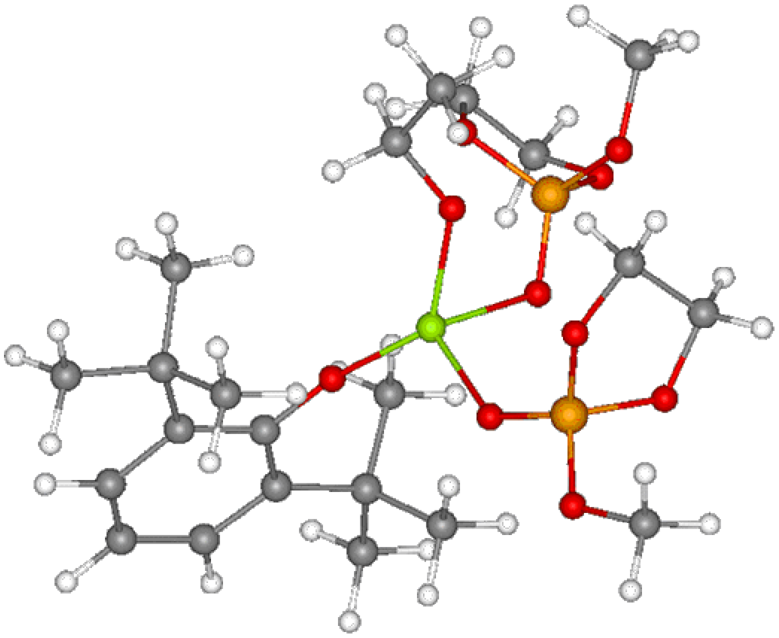

Zero-point vibrational energy	1639406.3 (Joules/Mol)
	391.82751 (Kcal/Mol)
Zero-point correction=	0.624417 (Hartree/Particle)
Thermal correction to Energy=	0.665903
Thermal correction to Enthalpy=	0.666847
Thermal correction to Gibbs Free Energy=	0.547305
Sum of electronic and zero-point Energies=	-2496.473564
Sum of electronic and thermal Energies=	-2496.432077
Sum of electronic and thermal Enthalpies=	-2496.431133
Sum of electronic and thermal Free Energies=	-2496.550676

cartesian

12	0.41053650	-0.26081076	0.00927469	1	-0.99606347	0.42068923	3.92087483
8	1.70083654	0.20168923	-1.33782530	1	-0.92176342	-0.48121077	2.39757466
8	-1.44706345	-0.08801077	0.07387469	1	1.42073655	1.32478929	-3.04362535
6	1.65293658	0.29098922	-2.73432517	8	1.01333654	-2.01641083	0.84527469
1	0.84523654	-0.33471078	-3.14752531	15	2.32193661	-2.67321086	1.09177470
6	2.96323657	-0.13601077	-3.39512539	8	3.31803656	-1.86601079	2.06067467
6	-2.75746346	-0.23241076	0.21807469	8	3.31543660	-2.83241081	-0.15822530
6	-3.53666353	-0.83991075	-0.81902528	6	4.49573660	-1.42741072	1.34067476
6	-4.91456366	-0.97861075	-0.62862527	6	4.20833635	-1.68381071	-0.14432532
1	-5.52156353	-1.43981075	-1.40082526	1	5.34263659	-2.01081085	1.71227467
6	-5.54876328	-0.53931075	0.52877468	1	4.64263630	-0.36821079	1.55047476
6	-4.78966331	0.06338924	1.52637470	1	3.69033647	-0.84441078	-0.61892533

1	-5.30016327	0.40738922	2.42017484
6	-3.40726352	0.23388924	1.40677476
6	-2.88956356	-1.32431078	-2.12922525
6	-2.61666346	0.91208923	2.54017472
6	-1.86916351	-2.44581079	-1.84572530
1	-1.11956346	-2.14651084	-1.11232531
1	-1.35426342	-2.74311066	-2.76802516
1	-2.37706351	-3.32891083	-1.44272530
6	-2.22156358	-0.13981077	-2.85652518
1	-1.47936344	0.35518926	-2.22922540
1	-2.97606349	0.60538924	-3.13312531
1	-1.73136353	-0.47991079	-3.77752519
6	-3.91676331	-1.91561079	-3.11122537
1	-4.67496347	-1.18321073	-3.40802526
1	-4.42716360	-2.79301071	-2.69992518
1	-3.39906335	-2.23751068	-4.02232552
6	-3.52086353	1.37238920	3.69767475
1	-4.03886366	0.53598922	4.17897463
1	-4.27116346	2.10148931	3.37317467
1	-2.90396357	1.85578930	4.46417427
6	-1.91006351	2.17748928	2.01087475
1	-1.26306343	1.94288921	1.16517472
1	-1.30366349	2.63438916	2.80407476
1	-2.65376353	2.91258931	1.68147469
6	-1.59826350	-0.07291076	3.14817476
1	-2.12096357	-0.91671073	3.61177468
1	5.09973669	-1.96281075	-0.70622528
8	2.07273650	-4.11221075	1.68447471
6	3.17173648	-4.96501112	2.06267476
1	3.79483652	-5.18691111	1.19377470
1	3.76103663	-4.49251080	2.85187483
1	2.71853662	-5.88081121	2.43847466
8	1.47173655	1.11868930	1.12057471
15	2.18853641	2.15588927	0.32037470
8	2.39523649	3.51798916	1.19717467
8	1.42913651	2.86898923	-0.91042531
6	1.36103654	4.45328903	0.85837471
6	1.14133656	4.25988913	-0.64302528
1	1.71523654	5.45428896	1.10787475
1	0.46003652	4.22528887	1.43537474
1	0.11253650	4.44938898	-0.95112526
1	1.82723653	4.86548901	-1.24212527
8	3.69383645	1.76108921	-0.00962530
6	4.52263641	2.66868925	-0.75712526
1	4.10643625	2.82318926	-1.75512528
1	4.62253666	3.61878920	-0.22612530
1	5.49823666	2.19028926	-0.83912528
1	2.90783644	-0.02351077	-4.48502541
1	3.80183649	0.47188926	-3.03512526
1	3.18493652	-1.18611073	-3.17512536
1	-6.62266350	-0.66091073	0.64997470

MTS-12_front

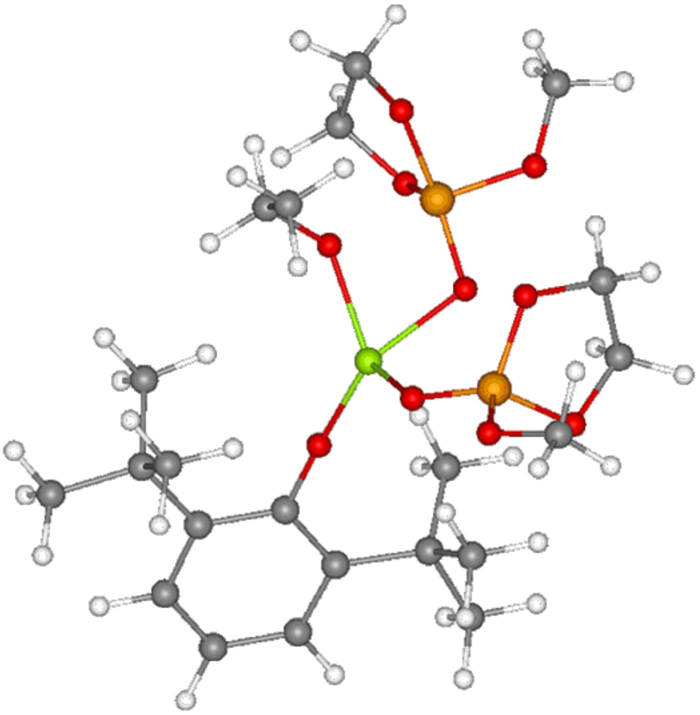

Zero-point vibrational energy	1641309.6 (Joules/Mol)
	392.28241 (Kcal/Mol)
Zero-point correction=	0.625142 (Hartree/Particle)
Thermal correction to Energy=	0.665915
Thermal correction to Enthalpy=	0.666859
Thermal correction to Gibbs Free Energy=	0.548622
Sum of electronic and zero-point Energies=	-2496.456592
Sum of electronic and thermal Energies=	-2496.415818
Sum of electronic and thermal Enthalpies=	-2496.414874
Sum of electronic and thermal Free Energies=	-2496.533112

cartesian

12	0.28057578	-0.00476795	0.28229997	1	-0.82382417	3.17853189	-2.21530008
8	1.32387578	-1.65776789	0.63599998	1	-0.99442422	2.25653195	-0.70680004
8	-1.53742421	0.03223205	-0.04360004	1	2.01597571	-3.32716799	1.65969992
6	1.51877582	-2.37086797	1.85219991	8	0.92287576	1.57533205	1.38509989
1	0.52227581	-2.61106801	2.24339986	15	2.02647567	2.55643201	1.19919991
6	2.31157565	-1.58666790	2.88800001	8	2.02627587	3.42923188	-0.14310004
6	-2.84522414	0.16013205	-0.22680004	8	3.49847579	1.93723202	1.07019997
6	-3.76002407	-0.49466795	0.65739995	6	3.00727582	2.89513183	-1.07230008
6	-5.13142395	-0.33806795	0.43629998	6	4.10847569	2.26533198	-0.20520005
1	-5.84352398	-0.82436794	1.09479988	1	3.36867571	3.73193192	-1.67010009

6	-5.62612438	0.42813206	-0.61370003	1	2.52267575	2.14543200	-1.69770014
6	-4.73052406	1.05693197	-1.47250009	1	4.47497559	1.34013212	-0.64910001
1	-5.13332415	1.64823210	-2.28830004	1	4.93157578	2.95523190	-0.00110004
6	-3.34622407	0.94663209	-1.31220007	8	1.98877585	3.56083202	2.41709995
6	-3.26192427	-1.36726797	1.82439995	6	2.94407582	4.63403177	2.52600002
6	-2.39762425	1.66363204	-2.29070020	1	3.95787573	4.23273182	2.59759998
6	-2.43482423	-0.52486795	2.81699991	1	2.85387588	5.30913210	1.67209995
1	-1.60982418	-0.00416796	2.32830000	1	2.69197583	5.16263199	3.44379997
1	-2.02632427	-1.15816796	3.61500001	8	1.85487580	-0.00056796	-0.94450003
1	-3.06512427	0.23913205	3.28499985	15	2.59367585	-1.34866798	-0.89140004
6	-2.43442416	-2.55146813	1.28379989	8	1.91077578	-2.45726800	-1.86000013
1	-1.57872415	-2.21636796	0.69589996	8	3.57617593	-2.41376805	-0.04440004
1	-3.05462408	-3.18216801	0.63720000	6	2.02197576	-3.76036811	-1.27710009
1	-2.07202411	-3.17536807	2.11159992	6	3.33627582	-3.75696802	-0.48280004
6	-4.41762400	-1.97896802	2.63629985	8	3.86817575	-0.74226797	-1.77150011
1	-5.04702425	-2.63816810	2.02909994	6	4.91687584	-1.56606793	-2.26570010
1	-5.05652428	-1.21396792	3.08999991	1	5.52437592	-1.97726786	-1.45410013
1	-4.00402403	-2.58356810	3.45179987	1	4.52297592	-2.37936807	-2.88610005
6	-3.15592408	2.43633199	-3.38450003	1	5.54037571	-0.92736793	-2.89569998
1	-3.79852438	3.22073197	-2.97040009	1	2.40847588	-2.17096806	3.81040001
1	-3.77172422	1.77693200	-4.00540018	1	3.31487584	-1.36006796	2.51749992
1	-2.43172407	2.92443204	-4.04680014	1	1.81107581	-0.64586794	3.13769984
6	-1.50192416	0.64293206	-3.02220011	1	-6.69832420	0.53223205	-0.76280004
1	-0.91962421	0.03363204	-2.33080006	1	3.29207587	-4.41516829	0.38959998
1	-0.80622423	1.15713203	-3.69740009	1	4.17817593	-4.06546783	-1.11310005
1	-2.11532426	-0.03586796	-3.62490010	1	2.03567576	-4.48976803	-2.08899999
6	-1.54332423	2.70293188	-1.53700006	1	1.15307581	-3.93376827	-0.63750005
1	-2.18202424	3.48863196	-1.11820006				

MI-2

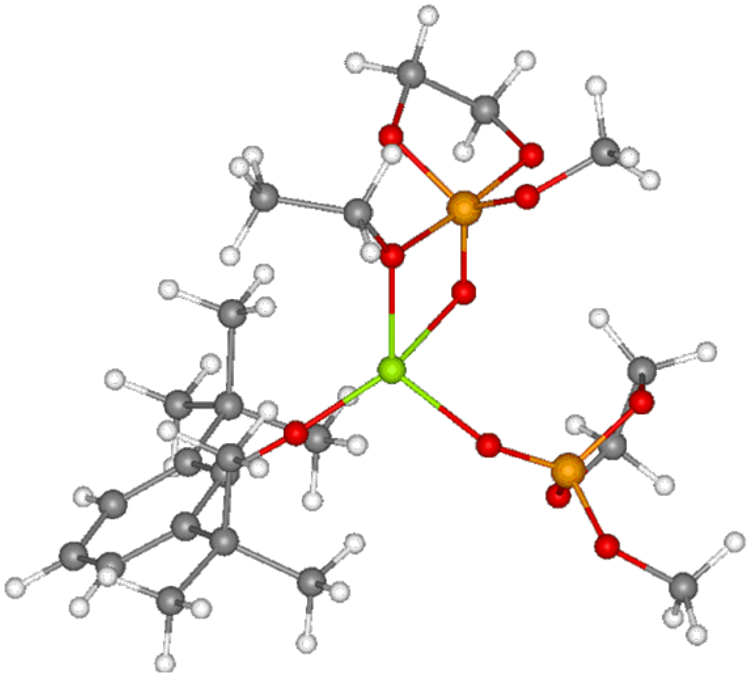

Zero-point vibrational energy	1643868.5 (Joules/Mol)
	392.89400 (Kcal/Mol)
Zero-point correction=	0.626116 (Hartree/Particle)
Thermal correction to Energy=	0.667507
Thermal correction to Enthalpy=	0.668451
Thermal correction to Gibbs Free Energy=	0.549132
Sum of electronic and zero-point Energies=	-2496.488615
Sum of electronic and thermal Energies=	-2496.447225
Sum of electronic and thermal Enthalpies=	-2496.446280
Sum of electronic and thermal Free Energies=	-2496.565600

cartesian

12	0.40965077	0.16324402	0.40965867	1	-0.26054922	-0.64395601	-3.51344132
8	1.38165081	-1.14505601	1.59255862	1	-0.62824929	0.37664402	-2.10824132
8	-1.41184926	0.10424402	0.11315867	1	2.22695065	-2.21815610	3.13935876
6	1.26205075	-1.77715588	2.87625861	8	1.00705075	2.09214401	0.63155866
6	0.16595076	-2.82685614	2.87855864	15	1.86575079	2.98484397	-0.18914133
1	1.05445075	-0.98825592	3.60785866	8	1.57025075	2.92744398	-1.76704133
6	-2.68734932	-0.06195597	-0.21684134	8	3.44295073	2.70274401	-0.24764132
6	-3.71524906	0.47494403	0.62185866	6	2.75785089	2.58334398	-2.51904130
6	-5.04834938	0.27384403	0.25275868	6	3.77115083	2.04324412	-1.50104129
1	-5.84524918	0.66924405	0.87385869	1	3.10675073	3.48874402	-3.02304125
6	-5.39964914	-0.42285597	-0.89884132	1	2.47655082	1.83074403	-3.25544143

6	-4.39484930	-0.93205601	-1.71394134	1	3.66755080	0.96604401	-1.35974133
1	-4.68664932	-1.46955609	-2.61034131	1	4.79785061	2.31524396	-1.74664128
6	-3.03974915	-0.77125597	-1.40884137	8	1.68635082	4.46114397	0.34075868
6	-3.38214922	1.26914406	1.89865863	6	2.35285068	5.56894398	-0.29484132
6	-1.96684933	-1.35285592	-2.34784126	1	3.43525076	5.42224407	-0.27984133
6	-2.57604933	2.53564405	1.54535866	1	1.99105084	5.68804407	-1.31894135
1	-1.64724922	2.29684401	1.02725863	1	2.09055066	6.44804430	0.29125869
1	-2.32734919	3.09714389	2.45485878	8	2.08045077	-0.42135596	-0.50814134
1	-3.16654921	3.19164395	0.89585871	15	2.79535079	-1.35045600	0.50555867
6	-2.59784913	0.39234403	2.89525867	8	4.18625069	-1.65595603	-0.44914132
1	-1.67254925	0.01654403	2.45655870	8	2.45885086	-2.96635604	0.55045867
1	-3.19764924	-0.47205597	3.20095873	6	4.00825071	-2.83215594	-1.20814133
1	-2.34644914	0.96554404	3.79645872	6	3.31445074	-3.78915596	-0.24654132
6	-4.64474916	1.74384403	2.64025879	1	4.98425102	-3.20155597	-1.53484130
1	-5.27504921	0.90924400	2.96625876	1	3.38335085	-2.63475609	-2.09034133
1	-5.25504923	2.41934395	2.03115869	1	2.69275069	-4.53425598	-0.74814135
1	-4.34694910	2.29644394	3.53865862	1	4.03095055	-4.29235601	0.41205868
6	-2.57644916	-2.05665612	-3.57384133	8	3.68005085	-0.71055597	1.71355867
1	-3.17654920	-1.37605596	-4.18724155	6	5.10645056	-0.60865599	1.74125862
1	-3.20284915	-2.90985608	-3.29314137	1	5.57895088	-1.59195590	1.69365871
1	-1.76834917	-2.43995595	-4.20754147	1	5.48005056	0.00764403	0.92145866
6	-1.12894917	-2.41915607	-1.61344135	1	5.34075069	-0.13295597	2.69595861
1	-0.66624916	-2.03695607	-0.70294130	1	0.06895077	-3.25945592	3.88075876
1	-0.33344921	-2.80135608	-2.26514125	1	-0.79834926	-2.39145613	2.60295868
1	-1.76394916	-3.26345611	-1.32354128	1	0.39985076	-3.62695599	2.17275858
6	-1.07044923	-0.22625598	-2.90234137	1	-6.44584942	-0.56425595	-1.15974128
1	-1.65874910	0.44984403	-3.53254128				

MTS-23

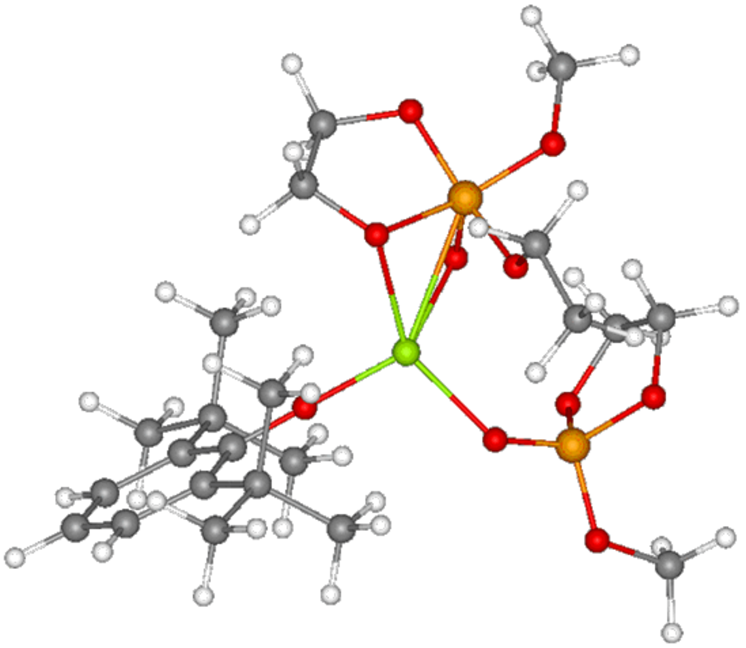

Zero-point vibrational energy	1643386.5 (Joules/Mol)
	392.77879 (Kcal/Mol)
Zero-point correction=	0.625933 (Hartree/Particle)
Thermal correction to Energy=	0.666641
Thermal correction to Enthalpy=	0.667585
Thermal correction to Gibbs Free Energy=	0.550969
Sum of electronic and zero-point Energies=	-2496.472669
Sum of electronic and thermal Energies=	-2496.431961
Sum of electronic and thermal Enthalpies=	-2496.431016
Sum of electronic and thermal Free Energies=	-2496.547633

cartesian

12	-0.46078813	-0.07986808	-0.08209602	1	0.91301191	1.99273193	3.36870384
8	-2.48428822	-1.05306804	-1.10749602	1	0.91201186	1.78903198	1.60400391
8	1.38161194	0.08873192	0.01360398	6	-2.78728819	-0.75856811	-3.46929598
6	-2.75938821	-1.75566804	-2.32859612	8	-1.19088817	1.73093200	-0.60479605
1	-3.72698808	-2.26016808	-2.22699594	15	-2.36728811	2.54853177	-0.20409602
1	-1.98288810	-2.51246810	-2.49289608	8	-2.74498820	2.51143193	1.34900391
6	2.69871187	0.22153193	0.09280398	8	-3.79758811	2.13223195	-0.79209602
6	3.50461197	0.11553192	-1.08599603	6	-3.96658802	1.76873195	1.58040392
6	4.89331198	0.21093193	-0.95479608	6	-4.52028799	1.35403192	0.19620398
1	5.52511168	0.12433192	-1.83249605	1	-4.64158821	2.43453193	2.12210393
6	5.50731182	0.41823190	0.27570397	1	-3.71168804	0.89563191	2.18000388
6	4.71571207	0.55553192	1.41080391	1	-4.35138798	0.29703194	-0.01009602

1	5.21011209	0.73443192	2.35990405	1	-5.57568789	1.60313201	0.07920398
6	3.32121181	0.47253191	1.35770392	8	-2.10258818	4.04253197	-0.63319600
6	2.87721181	-0.08456808	-2.47829604	6	-3.06578803	5.07783222	-0.35169601
6	2.49121189	0.66803193	2.64060402	1	-4.01688814	4.85243225	-0.83899605
6	1.93631196	1.09133196	-2.81299615	1	-3.19948816	5.18393230	0.72700393
1	1.16201186	1.23183191	-2.05769610	1	-2.64418817	5.99283218	-0.76419604
1	1.45181191	0.93043190	-3.78469610	8	-1.93638813	-0.66336811	1.22650397
1	2.50411177	2.02623200	-2.87339616	15	-2.33268809	-1.88506806	0.37540397
6	2.11921191	-1.42506802	-2.54819608	8	-2.15548801	-3.28226805	1.26180398
1	1.32821190	-1.49046803	-1.80049610	8	-0.69828808	-2.23676825	-0.30899602
1	2.80731177	-2.26076818	-2.37699604	6	-0.80588812	-3.57026815	1.60860395
1	1.66931200	-1.55966806	-3.54029608	6	0.00811189	-3.27786803	0.34980398
6	3.93321180	-0.12516807	-3.59779596	1	-0.75458813	-4.61816788	1.90980399
1	4.62861204	-0.96406811	-3.48509598	1	-0.50138813	-2.93066812	2.44340396
1	4.51591206	0.80043191	-3.65199614	1	1.02341187	-2.94686818	0.57900393
1	3.42891192	-0.24916808	-4.56309605	1	0.04901189	-4.14746809	-0.31559601
6	3.36911178	0.95323193	3.87230396	8	-3.96598816	-2.11186814	0.33580399
1	3.96691179	1.86293197	3.75290394	6	-4.69828796	-2.84896827	1.31940401
1	4.04631186	0.12363192	4.10240412	1	-4.53238821	-3.92236805	1.20970392
1	2.72491193	1.09993196	4.74690390	1	-4.42638826	-2.54566813	2.33400393
6	1.68521190	-0.60426807	2.96880388	1	-5.75148821	-2.62006807	1.13760400
1	0.99731183	-0.86216807	2.16320395	1	-3.01908803	-1.27436805	-4.40659618
1	1.09901190	-0.46506810	3.88550401	1	-3.54798818	0.00793192	-3.29829597
1	2.36111188	-1.45306802	3.12310386	1	-1.81888807	-0.26376808	-3.58309603
6	1.55111194	1.88073194	2.48340392	1	6.59051180	0.48293191	0.34750399
1	2.13561177	2.80023193	2.36880398				

MI-3

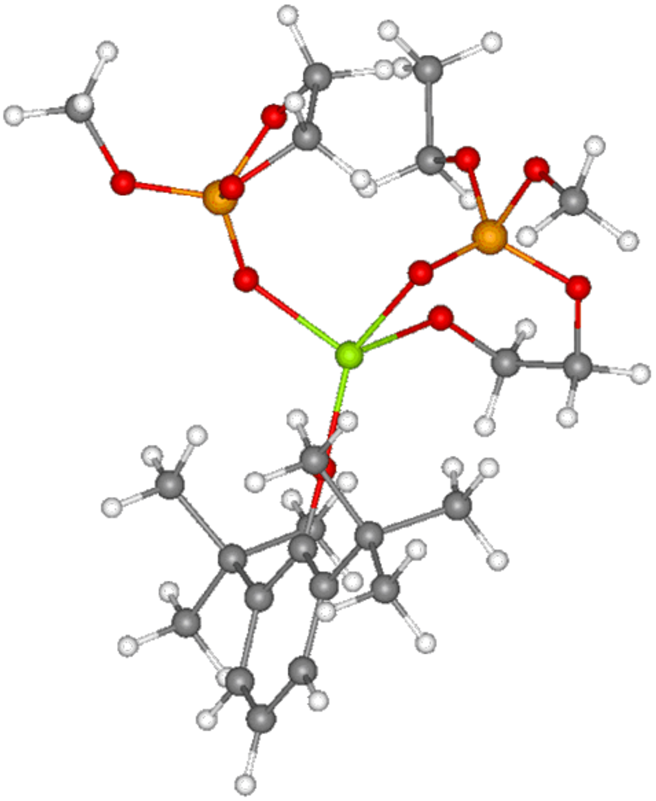

Zero-point vibrational energy	1643197.0 (Joules/Mol)
	392.73350 (Kcal/Mol)
Zero-point correction=	0.625861 (Hartree/Particle)
Thermal correction to Energy=	0.667501
Thermal correction to Enthalpy=	0.668446
Thermal correction to Gibbs Free Energy=	0.549508
Sum of electronic and zero-point Energies=	-2496.490683
Sum of electronic and thermal Energies=	-2496.449042
Sum of electronic and thermal Enthalpies=	-2496.448098
Sum of electronic and thermal Free Energies=	-2496.567035

cartesian

12	0.26832652	0.02891735	0.22843331	1	-0.89907342	-0.87418264	-3.62356663
8	3.69682670	-1.03028262	1.22113335	1	-0.97147352	0.19851735	-2.21186662
8	-1.57337356	0.05761734	0.09073333	1	2.99442649	0.64781737	2.22873354
6	3.61282659	-0.23198265	2.41413355	8	1.13502645	1.83421731	-0.07396667
6	5.02822638	0.15801734	2.79023337	15	2.30052662	2.34731722	-0.84406668
1	3.14902663	-0.81368262	3.21433353	8	2.44092655	1.90691733	-2.37596655
6	-2.88367343	0.09791735	-0.12576666	8	3.73302650	1.85031736	-0.32856667
6	-3.73677349	0.80691737	0.77853334	6	3.44752669	0.86461735	-2.49686646

6	-5.11367321	0.80421734	0.53603333
1	-5.78077364	1.33021736	1.21073329
6	-5.67097330	0.14531735	-0.55456668
6	-4.83347321	-0.52508265	-1.43906665
1	-5.28387356	-1.02558267	-2.28966665
6	-3.44737339	-0.56758261	-1.26056671
6	-3.17327332	1.57501733	1.98953331
6	-2.56567335	-1.31468260	-2.27936649
6	-2.22547340	2.69541717	1.51593328
1	-1.40347338	2.31101727	0.91163331
1	-1.80107331	3.22901726	2.37593341
1	-2.77217340	3.42291737	0.90583330
6	-2.43817329	0.61571735	2.94693351
1	-1.62617350	0.09311734	2.44013333
1	-3.12947345	-0.13818264	3.34023333
1	-2.01907349	1.16701734	3.79813337
6	-4.27647352	2.25521731	2.81883335
1	-4.98867321	1.53441739	3.23453355
1	-4.83557320	2.99581718	2.23723340
1	-3.81747341	2.78351736	3.66223335
6	-3.38597345	-1.94148266	-3.42106652
1	-3.93707323	-1.19048262	-3.99656653
1	-4.09937334	-2.68878269	-3.05796647
1	-2.70677352	-2.45028281	-4.11506701
6	-1.81787348	-2.47738266	-1.59666669
1	-1.20657349	-2.13058281	-0.76266670
1	-1.16347337	-2.99008274	-2.31206656
1	-2.53227329	-3.20958281	-1.20436668
6	-1.57477355	-0.33848265	-2.94556665
1	-2.11707330	0.41211733	-3.53066659

6	4.44272661	1.09821737	-1.34706664
1	3.90732670	0.97901738	-3.47866654
1	2.94822669	-0.09948265	-2.40276647
1	4.76852655	0.15991735	-0.89776665
1	5.30332661	1.69971740	-1.65056670
8	2.24772644	3.92411733	-0.80686671
6	3.25982666	4.72041702	-1.45456672
1	4.23022652	4.55481720	-0.98056668
1	3.30352664	4.48571730	-2.52036667
1	2.95402670	5.75601721	-1.31646669
8	1.72392654	-1.00528264	-0.66266668
15	2.52572656	-1.78088260	0.40713331
8	2.02012658	-3.21408272	0.99613333
8	1.16712654	-1.01538265	1.69013333
6	0.81632650	-3.30688286	1.76293337
6	0.69402653	-2.01478267	2.55683351
1	0.90812653	-4.19448280	2.39253354
1	-0.02687347	-3.43258286	1.07593334
1	-0.34417349	-1.82828259	2.84913349
1	1.30932653	-2.05748272	3.46803355
8	3.68002653	-2.48408270	-0.53786665
6	3.25802660	-3.35738277	-1.58016670
1	2.80382657	-4.26558256	-1.17096663
1	2.54432654	-2.86478281	-2.25016665
1	4.15442657	-3.62948275	-2.14206648
1	5.01112652	0.75081736	3.71033335
1	5.64592648	-0.72808266	2.95933342
1	5.49062681	0.76041734	2.00393343
1	-6.74617338	0.15901735	-0.71646667

MI-4

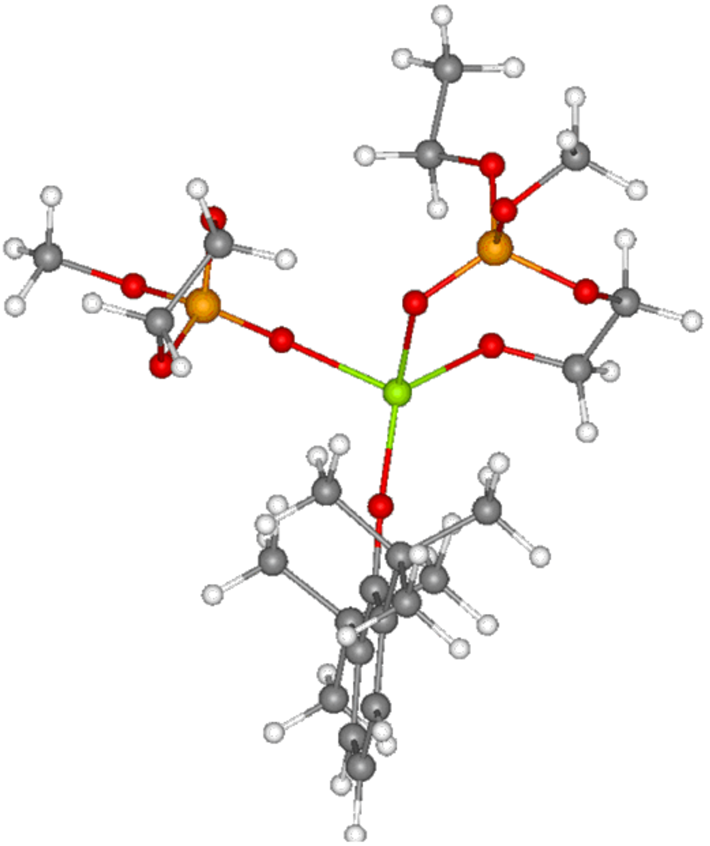

Zero-point vibrational energy	1640580.5 (Joules/Mol)
	392.10815 (Kcal/Mol)
Zero-point correction=	0.624864 (Hartree/Particle)
Thermal correction to Energy=	0.667099
Thermal correction to Enthalpy=	0.668043
Thermal correction to Gibbs Free Energy=	0.546623
Sum of electronic and zero-point Energies=	-2496.494176
Sum of electronic and thermal Energies=	-2496.451941
Sum of electronic and thermal Enthalpies=	-2496.450997
Sum of electronic and thermal Free Energies=	-2496.572417

cartesian

12	0.39618522	-0.00831600	0.80612534	1	-0.33831477	-0.68631601	-3.48837471
8	3.54258537	-2.15861607	-1.44747472	1	-0.63811475	0.18698400	-1.96587467
8	-1.36531472	-0.18701601	0.21162534	1	5.41748524	-2.85931611	-0.85347468
6	4.53808498	-3.19411612	-1.40997469	8	0.95178527	1.96198404	0.72722536
1	4.81038523	-3.38791609	-2.44717479	15	1.35068536	2.95688391	-0.29767466
1	4.12698507	-4.10021591	-0.95947468	8	0.39468524	3.05168390	-1.58157468
6	-2.64871478	-0.36481601	-0.06987467	8	2.73628521	2.69568396	-1.07477462

6	-3.66251469	0.07508401	0.84232533	6	1.11698532	2.76018405	-2.80207467
6	-5.00301504	-0.12451599	0.49862534	6	2.43178535	2.10508394	-2.36687469
1	-5.78631496	0.20168400	1.17482531	1	1.27628517	3.70498395	-3.32907486
6	-5.38011503	-0.73411602	-0.69337469	1	0.49808526	2.09408402	-3.40257478
6	-4.39211512	-1.16281605	-1.57307470	1	2.33418536	1.02518404	-2.23507476
1	-4.70201492	-1.64001596	-2.49717474	1	3.26118541	2.33858395	-3.03437471
6	-3.03171468	-0.99821603	-1.29727471	8	1.46518517	4.37308407	0.38792533
6	-3.30861473	0.75348401	2.17912531	6	1.75338531	5.55788422	-0.38107467
6	-1.98071480	-1.50101602	-2.30407476	1	2.72298527	5.46388435	-0.87557465
6	-2.52911472	2.05998397	1.92722535	1	0.95998526	5.73498440	-1.11077464
1	-1.63121486	1.89248395	1.33272529	1	1.78208518	6.37598419	0.33662534
1	-2.23391461	2.52318406	2.87722516	8	1.87628531	-0.57601601	-0.52157468
1	-3.15691471	2.77548409	1.38372529	15	2.88228536	-1.61151600	-0.10287467
6	-2.49721479	-0.20691600	3.07182527	8	2.38328528	-2.94841599	0.58532536
1	-1.58361483	-0.55391598	2.58772516	8	1.26348519	-0.94961596	2.21922517
1	-3.09581470	-1.09091604	3.31862521	6	2.24408531	-3.11081600	2.02972531
1	-2.21691465	0.28428400	4.01192522	6	1.08688521	-2.27861595	2.55992532
6	-4.55741501	1.14458394	2.99002528	1	3.18908525	-2.80691600	2.48962522
1	-5.16711473	0.27548400	3.25942516	1	2.09378529	-4.18401575	2.17062521
1	-5.19241476	1.86008406	2.45662522	1	0.14578524	-2.70831609	2.17102528
1	-4.24181509	1.62268400	3.92452526	1	1.06778526	-2.43271589	3.65522528
6	-2.61491466	-2.13541603	-3.55437469	8	4.10738516	-1.08261597	0.76332533
1	-3.24151468	-1.42761600	-4.10787487	6	4.10708523	0.20038401	1.46172535
1	-3.22171474	-3.01371598	-3.31037474	1	3.97438526	0.98998398	0.71772534
1	-1.81931472	-2.46761608	-4.23167467	1	3.25978541	0.20028400	2.15102530
6	-1.10101485	-2.58901596	-1.65707469	6	5.43298483	0.32948402	2.17612529
1	-0.61651474	-2.22701597	-0.75047469	1	5.46268511	1.28628397	2.70632529
1	-0.32201475	-2.92431593	-2.35317469	1	5.56278515	-0.47031599	2.91002512
1	-1.71221471	-3.45731592	-1.38757467	1	6.26998520	0.29908401	1.47282529
6	-1.11541486	-0.32541600	-2.80157471	1	-6.43161488	-0.87481600	-0.93257469
1	-1.73981476	0.40008399	-3.33647490				

MI-1m

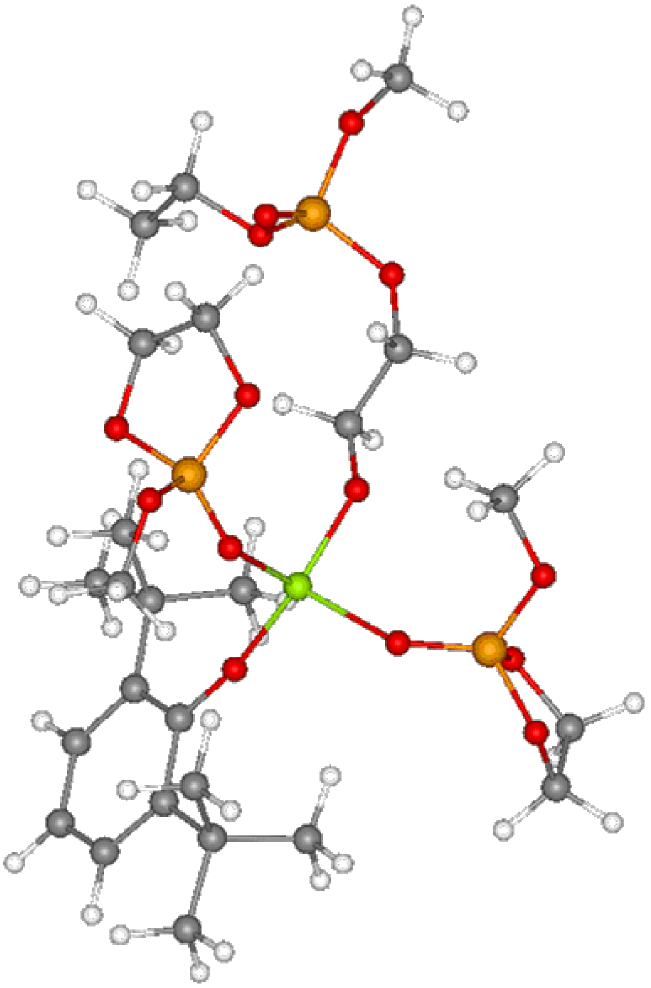

Zero-point vibrational energy	1944471.0 (Joules/Mol)
	464.73972 (Kcal/Mol)
Zero-point correction=	0.740610 (Hartree/Particle)
Thermal correction to Energy=	0.793970
Thermal correction to Enthalpy=	0.794915
Thermal correction to Gibbs Free Energy=	0.643328
Sum of electronic and zero-point Energies=	-3257.061075
Sum of electronic and thermal Energies=	-3257.007714
Sum of electronic and thermal Enthalpies=	-3257.006770
Sum of electronic and thermal Free Energies=	-3257.158357

cartesian

12	-0.78191978	0.80300331	0.25762117	6	3.13168025	0.86020327	3.39682126
8	0.83498025	1.41350329	-0.52077883	6	2.86308026	-0.56529677	2.91732121
8	-1.97581971	-0.58359671	-0.17047884	1	3.42058015	0.90120327	4.45042086
6	1.98348033	0.91010332	-1.10357881	1	3.86988020	1.35500324	2.76872134

1	1.86898029	0.72880328	-2.18597865
1	2.30588007	-0.05429673	-0.66697884
6	-2.67501974	-1.69249678	-0.35637885
6	-2.06531978	-2.85199666	-0.93887889
6	-2.83681989	-4.00569677	-1.10437882
1	-2.39181972	-4.89329672	-1.54187882
6	-4.17431974	-4.06279659	-0.72737885
6	-4.76741982	-2.93019676	-0.17997883
1	-5.81441975	-2.98629665	0.09952117
6	-4.06011963	-1.74009681	0.01262116
6	-0.59221977	-2.84669685	-1.38607883
6	-4.78031969	-0.50989676	0.59402114
6	0.33268026	-2.62909675	-0.17207883
1	0.08858025	-1.71499681	0.37112117
1	1.38138032	-2.57369685	-0.48997885
1	0.23568025	-3.46039677	0.53512114
6	-0.35921979	-1.76519680	-2.46047878
1	-0.62621975	-0.76899672	-2.10657883
1	-0.96761972	-1.97889686	-3.34657884
1	0.69298023	-1.75059676	-2.76957870
6	-0.16281976	-4.18299675	-2.01817870
1	-0.74441975	-4.42299652	-2.91477871
1	-0.24641974	-5.02189684	-1.31877887
1	0.88838023	-4.11509657	-2.32167864
6	-6.26611948	-0.77999675	0.89072114
1	-6.40481949	-1.56969678	1.63712120
1	-6.82571936	-1.05659676	-0.00927883
1	-6.72401953	0.13130328	1.29262114
6	-4.73731947	0.65070331	-0.42107886
1	-3.70921993	0.90680325	-0.67797887
1	-5.22951937	1.53960323	-0.00387883
1	-5.26451969	0.36560327	-1.33907890
6	-4.13671970	-0.08379671	1.92862117
1	-4.24311972	-0.88479674	2.66942120
1	-4.62931967	0.81360328	2.32482123
1	-3.07411981	0.12820329	1.81142116
6	3.11018014	1.91980326	-0.89847887
8	-0.40501976	0.71120328	2.26802135
15	0.64738023	0.53580326	3.30532122
8	1.86738026	1.56330323	3.25702119
8	1.47488034	-0.83399677	3.24612117

1	2.99818015	-0.65699673	1.83972120
1	3.47338009	-1.30459678	3.43492126
8	0.04948026	0.65800327	4.76662111
6	-1.32901967	0.31890327	5.04992104
1	-1.98221982	0.68850327	4.25912094
1	-1.42581975	-0.76419675	5.14692116
1	-1.56361973	0.80240327	5.99712086
8	-1.77261972	2.58810329	0.15462117
15	-1.81491971	3.70770335	-0.82487887
8	-3.18701982	4.53910303	-0.89547884
8	-1.75891972	3.23600316	-2.35997868
6	-3.89301991	4.21950340	-2.11417866
6	-2.80141973	3.87070322	-3.12727880
1	-4.47271967	5.09790325	-2.39887881
1	-4.55891943	3.37460327	-1.92227876
1	-3.13741994	3.15920329	-3.88177896
1	-2.38731980	4.76090336	-3.60927868
8	-0.72921973	4.82210302	-0.57177883
6	0.40698025	4.58150339	0.30762118
1	0.08268026	4.70280313	1.34242117
1	0.81228024	3.58060336	0.13912116
1	1.13378024	5.35040331	0.04802117
8	4.39028025	1.47060323	-1.43347883
1	2.89718008	2.84540319	-1.43727887
1	3.22478008	2.14110327	0.16622117
1	-4.74891949	-4.97559690	-0.86547887
15	5.32568026	0.49280328	-0.60427886
8	5.15968037	-0.90089679	-1.35967886
8	6.80798054	0.91180325	-1.05567884
8	5.10408020	0.48060328	0.86602116
6	7.39258051	2.09380341	-0.48817885
6	5.92628050	-2.04749680	-0.91587889
6	5.49418020	-3.24299669	-1.73557889
1	6.98948050	-1.83019686	-1.05937886
1	5.74068022	-2.20559669	0.15122117
1	6.85728025	2.98380327	-0.83137888
1	7.37968063	2.04400325	0.60362118
1	8.42168045	2.12820339	-0.84587884
1	6.06318045	-4.12519693	-1.42677879
1	4.43068027	-3.44959664	-1.59247887
1	5.67478037	-3.06909680	-2.79937863

MTS-45

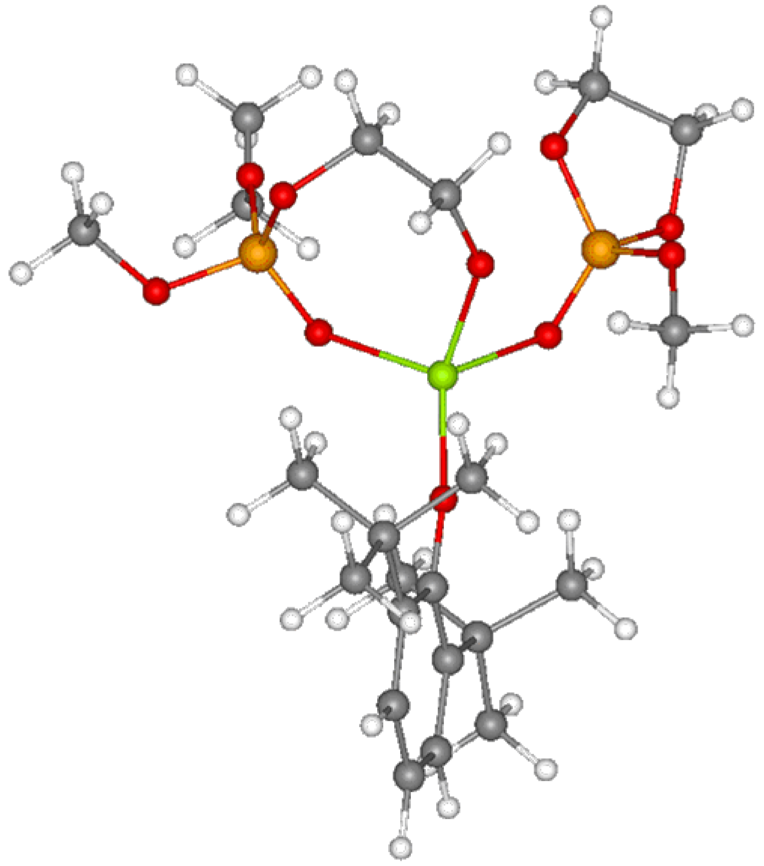

Zero-point vibrational energy	1639699.4 (Joules/Mol)
	391.89757 (Kcal/Mol)
Zero-point correction=	0.624529 (Hartree/Particle)
Thermal correction to Energy=	0.665930
Thermal correction to Enthalpy=	0.666874
Thermal correction to Gibbs Free Energy=	0.547817
Sum of electronic and zero-point Energies=	-2496.495346
Sum of electronic and thermal Energies=	-2496.453944
Sum of electronic and thermal Enthalpies=	-2496.453000
Sum of electronic and thermal Free Energies=	-2496.572057

cartesian

12	-0.30504012	-0.26997203	0.07067200	1	1.46065986	2.71562815	2.46107197
8	-1.45064020	3.77982807	-0.75682801	1	1.22625983	1.88042796	0.91127199
8	1.55065989	-0.31307203	0.00417200	1	-3.12283993	5.03772831	-0.76342803
6	-2.11214018	4.99322796	-0.34952801	8	-1.26494014	-1.99667203	-0.42202801
1	-1.51104009	5.80832815	-0.75002801	15	-2.31073999	-2.49837208	0.53067201
1	-2.14654016	5.05272818	0.73977202	8	-3.02424002	-3.76087189	-0.21942800
6	2.86236000	-0.18627203	-0.14542800	8	-3.69343996	-1.70147204	0.82997197

6	3.51796007	-0.76787204	-1.27792799	6	-4.38334036	-3.87587214	0.19787200
6	4.90455961	-0.63027203	-1.39012802	6	-4.84944010	-2.43257189	0.36997199
1	5.42265987	-1.06577206	-2.23822784	1	-4.43884039	-4.43587208	1.13827205
6	5.65915966	0.05292796	-0.44212800	1	-4.93624020	-4.40857172	-0.57722801
6	5.01495981	0.62272799	0.65087199	1	-5.17574024	-1.99557209	-0.57822800
1	5.61765957	1.15542793	1.37917197	1	-5.63394022	-2.32067204	1.12027204
6	3.63165998	0.52662796	0.82967198	8	-1.83574009	-3.16087198	1.89127195
6	2.73176003	-1.53137207	-2.35952806	6	-0.49244010	-3.05767202	2.41087198
6	2.96815991	1.18592799	2.05297184	1	0.24055991	-3.20447206	1.61527205
6	1.71425974	-0.59507203	-3.04272795	1	-0.36124012	-2.08047199	2.87237215
1	1.04075980	-0.12517203	-2.32472801	1	-0.40354010	-3.85777187	3.14567184
1	1.11075985	-1.14837205	-3.77322817	8	-1.12524009	1.29762793	-0.90402800
1	2.23456001	0.20982796	-3.57382822	15	-2.07084012	2.35712814	-0.44032800
6	2.02376008	-2.75717211	-1.74742806	8	-2.42584014	2.43552780	1.09037197
1	1.32795990	-2.47457218	-0.95782799	8	-1.51014018	-0.39027202	1.57367194
1	2.76225996	-3.44467211	-1.31962800	6	-3.14213991	1.38792801	1.82967198
1	1.46155989	-3.30097198	-2.51662779	6	-2.18004012	0.40752795	2.48117208
6	3.64195991	-2.06947184	-3.47822809	1	-3.81724000	0.87202799	1.14447200
1	4.38175964	-2.78487206	-3.10302782	1	-3.72244000	1.93702805	2.57567215
1	4.17275953	-1.26927209	-4.00522804	1	-1.48234010	0.98022795	3.11587191
1	3.02745986	-2.59457207	-4.21852827	1	-2.80134010	-0.19907203	3.16507196
6	3.98715997	1.88272798	2.97267199	8	-3.49544001	2.26282787	-1.15632796
1	4.52675962	2.68812799	2.46307182	6	-3.56944013	1.87992799	-2.56052780
1	4.72235966	1.18202794	3.38287210	1	-3.05944014	2.64302778	-3.15642786
1	3.45756006	2.33222818	3.82067204	1	-3.04313993	0.93102795	-2.68752813
6	2.25345993	0.12822796	2.91747189	6	-5.02914047	1.77182794	-2.93752813
1	1.48795986	-0.40207204	2.35027218	1	-5.11184025	1.48182797	-3.98902798
1	1.77825987	0.60032797	3.78697205	1	-5.53374004	1.01552796	-2.33092785
1	2.97375989	-0.61057204	3.28637218	1	-5.54364014	2.72672796	-2.80222797
6	1.97605979	2.27542782	1.59797204	1	6.73715973	0.14172797	-0.55492800
1	2.50616002	3.07862806	1.07447195				

MI-5

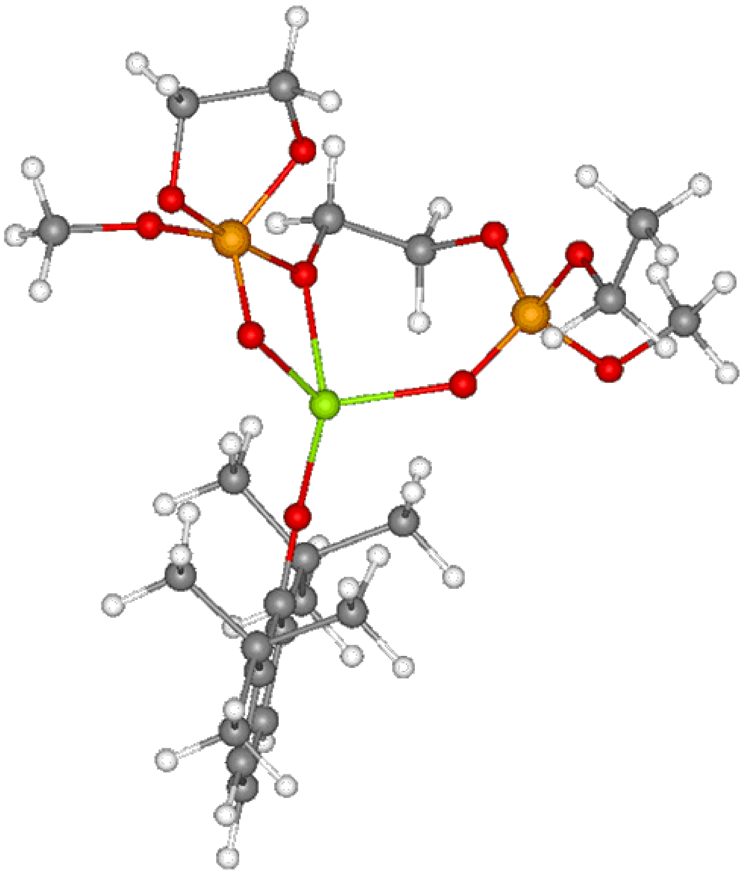

Zero-point vibrational energy	1642692.3 (Joules/Mol)
	392.61288 (Kcal/Mol)
Zero-point correction=	0.625668 (Hartree/Particle)
Thermal correction to Energy=	0.667186
Thermal correction to Enthalpy=	0.668130
Thermal correction to Gibbs Free Energy=	0.547247
Sum of electronic and zero-point Energies=	-2496.494973
Sum of electronic and thermal Energies=	-2496.453455
Sum of electronic and thermal Enthalpies=	-2496.452511
Sum of electronic and thermal Free Energies=	-2496.573394

cartesian

12	-0.28790274	-0.37243477	-0.22901058	1	1.43029714	-3.02943468	-2.74811053
8	-1.45250273	3.79756522	-0.24011061	1	1.34929729	-2.36803460	-1.11051059
8	1.54509735	-0.21953475	-0.03601059	1	-3.07170272	5.11586523	-0.36891058
6	-2.18480277	4.93866539	0.24478941	8	-1.33290267	-1.98763478	-0.65801060
1	-1.50480270	5.78576517	0.16848941	15	-2.35600281	-2.20033479	0.46668941
1	-2.47880268	4.79086542	1.28658938	8	-3.06700277	-3.58933473	-0.20071059
6	2.86729741	-0.15133476	-0.13311060	8	-3.82730269	-1.45853484	0.56378943

6	3.62339735	0.44676524	0.92398942	6	-4.43580294	-3.68043470	0.12608941
6	5.01479721	0.50106525	0.80438942	6	-4.92530298	-2.23953462	0.08568941
1	5.60829687	0.94816524	1.59528935	1	-4.58210278	-4.11583471	1.12668943
6	5.67799711	-0.00373476	-0.30931059	1	-4.93630314	-4.31463480	-0.61011058
6	4.93589687	-0.57903475	-1.33551061	1	-5.15870285	-1.92293477	-0.93611062
1	5.46929693	-0.96713477	-2.19701052	1	-5.78340292	-2.05023479	0.73498940
6	3.54169726	-0.67033476	-1.28341055	8	-1.88990283	-2.89903474	1.86588943
6	2.93039727	1.03426516	2.16678929	6	-1.58460283	-4.29873466	1.94058943
6	2.76869726	-1.32763481	-2.44131064	1	-2.49060273	-4.90893459	1.91238940
6	2.13569736	-0.05753476	2.91218948	1	-0.92020273	-4.60213470	1.12878942
1	1.39639735	-0.53333479	2.26698947	1	-1.07890272	-4.43583488	2.89808941
1	1.62259722	0.36896524	3.78448939	8	-1.09710276	1.39586520	-0.78481060
1	2.81239724	-0.83933473	3.27438951	15	-2.12250280	2.35206532	-0.27191058
6	2.00499725	2.19436526	1.74638939	8	-2.74890280	2.07666540	1.15368938
1	1.28029728	1.87746513	0.99408942	8	-1.71660280	-0.62393475	1.20108938
1	2.59149742	3.01076531	1.31078947	6	-1.97850275	1.50306523	2.25068951
1	1.47089720	2.59706521	2.61818933	6	-2.22050285	0.01266524	2.35198951
6	3.93089747	1.61696517	3.18008947	1	-2.33490276	2.01166534	3.14938951
1	4.51879692	2.43836522	2.75708938	1	-0.91640276	1.72886515	2.11988950
1	4.62249708	0.85846525	3.56168938	1	-1.71500278	-0.35783476	3.25448942
1	3.38089728	2.01856542	4.03918934	1	-3.29350281	-0.17713475	2.44668937
6	3.69649720	-1.80253482	-3.57441068	8	-3.44720268	2.45426536	-1.13561058
1	4.40549707	-2.56553459	-3.23641062	6	-3.35400271	2.47386527	-2.59141064
1	4.26319695	-0.97873473	-4.02141047	1	-2.75050259	1.62066519	-2.91031051
1	3.09049726	-2.25173473	-4.36951065	6	-4.75870275	2.40266538	-3.14331055
6	1.79649734	-0.31713477	-3.08571053	1	-2.84830260	3.39576530	-2.89521050
1	1.13959718	0.15796524	-2.35421062	1	-4.71870279	2.41456532	-4.23641062
1	1.17689729	-0.80783474	-3.84631062	1	-5.25290298	1.48196518	-2.82491064
1	2.35529733	0.48956525	-3.57271051	1	-5.35680294	3.25466537	-2.81021070
6	2.02229738	-2.58143473	-1.94101059	1	6.76189709	0.05066524	-0.37701058
1	2.74209738	-3.33063459	-1.59341061				

MTS-56

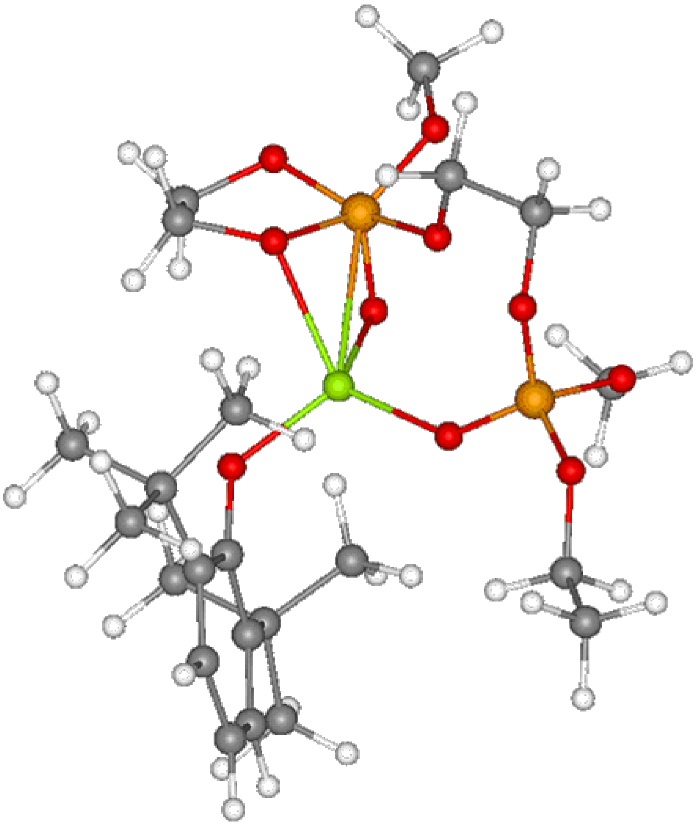

Zero-point vibrational energy	1644056.5 (Joules/Mol)
	392.93894 (Kcal/Mol)
Zero-point correction=	0.626188 (Hartree/Particle)
Thermal correction to Energy=	0.666465
Thermal correction to Enthalpy=	0.667409
Thermal correction to Gibbs Free Energy=	0.551891
Sum of electronic and zero-point Energies=	-2496.482601
Sum of electronic and thermal Energies=	-2496.442324
Sum of electronic and thermal Enthalpies=	-2496.441380
Sum of electronic and thermal Free Energies=	-2496.556898

cartesian

12	-0.81081843	-0.35784656	0.18830393	1	1.19228148	-2.46884656	-2.18579602
8	-2.04101849	3.30685353	1.43130386	1	-2.79001832	1.47135341	2.12430406
8	0.77068156	-1.33184659	-0.16919607	8	-2.40371847	-1.02724659	1.25570393
6	-2.34831834	2.38915348	2.51310396	15	-3.39051843	-0.99164653	0.07590393
1	-1.44191837	2.16395354	3.07770395	8	-3.89701843	-2.56954646	-0.16579607
6	2.04558158	-0.94294655	-0.05739607	8	-2.26351833	-1.22924650	-1.28219604
6	2.71358156	-1.02894652	1.20380390	6	-2.86111832	-3.40614653	-0.65509611
6	4.00568151	-0.50324655	1.30930388	6	-2.17911839	-2.57984662	-1.74009609

1	4.52618122	-0.52874655	2.26100397	1	-3.31501842	-4.31924677	-1.04529607
6	4.66518164	0.05475347	0.21870393	1	-2.16861844	-3.65654659	0.15630393
6	4.04468155	0.04155347	-1.02639604	1	-1.12901843	-2.84494662	-1.87389612
1	4.59418154	0.43445349	-1.87529612	1	-2.70621824	-2.65474653	-2.69719601
6	2.75368166	-0.46594656	-1.20519614	8	-4.92571878	-0.53104657	0.38970396
6	2.06138158	-1.72384655	2.41530395	6	-5.96411848	-1.41514659	0.83400393
6	2.14988160	-0.55504656	-2.62059593	1	-6.29831839	-2.06514645	0.02370393
6	1.74978161	-3.19254661	2.06020403	1	-5.63061857	-2.02544665	1.67570388
1	1.09988165	-3.25284648	1.18500388	1	-6.78131866	-0.76384652	1.15140390
1	1.25238156	-3.69304657	2.90070415	8	-0.41801843	1.57695341	0.43240395
1	2.67378163	-3.73834658	1.84060395	15	-1.09041834	2.90265346	0.22310393
6	0.76238161	-1.01884651	2.86680412	8	-1.92911839	3.10295343	-1.10299611
1	-0.10141841	-1.33354652	2.27720404	8	-3.07651830	0.67585343	-0.28739604
1	0.86258155	0.07155347	2.82410407	6	-3.34411836	2.81765342	-1.22129607
1	0.52008158	-1.28704655	3.90220404	6	-3.59871840	1.34085345	-1.42129612
6	2.99708176	-1.74254656	3.63770413	1	-3.67531824	3.39185357	-2.08859587
1	3.21428156	-0.73524654	4.01120377	1	-3.85481834	3.18225336	-0.32659605
1	3.94678187	-2.24324656	3.42460394	1	-4.68321848	1.18885350	-1.50079608
1	2.51448154	-2.29504657	4.45140409	1	-3.11491847	0.97915345	-2.33679605
6	3.12488174	-0.04914653	-3.69889593	8	-0.04621843	4.07295370	0.03910393
1	4.06858158	-0.60394657	-3.69659591	6	1.19918156	4.07435369	0.79870391
1	3.35118175	1.01725352	-3.58759594	1	1.64618158	3.07935357	0.73940390
1	2.67108154	-0.18414654	-4.68709612	6	2.09948158	5.13085365	0.20170394
6	0.86658162	0.28805345	-2.76269603	1	0.95838159	4.30015326	1.84230387
1	0.02728158	-0.16314654	-2.23109603	1	5.66698122	0.46305346	0.33080393
1	0.57228160	0.35775346	-3.81739593	1	-3.05891848	2.91995335	3.14570403
1	1.01608157	1.30575347	-2.38819599	1	3.03958154	5.16065359	0.76030391
6	1.84078169	-2.03084660	-2.94699597	1	2.32648158	4.90095329	-0.84199607
1	2.76518154	-2.61724663	-2.98449588	1	1.63358164	6.11825323	0.25180393
1	1.34758162	-2.11414647	-3.92409587				

MI-6

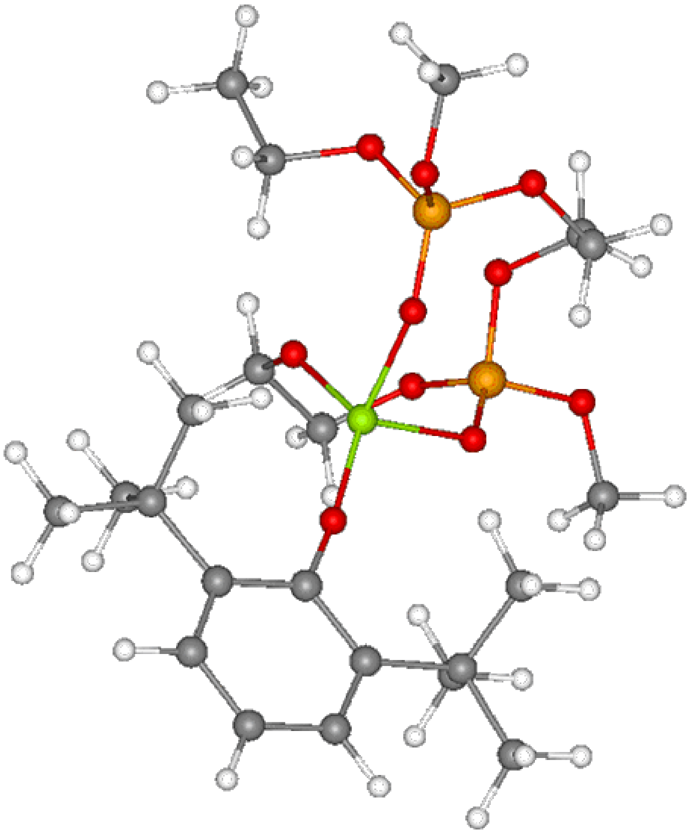

Zero-point vibrational energy	1640789.1 (Joules/Mol)
	392.15801 (Kcal/Mol)
Zero-point correction=	0.624944 (Hartree/Particle)
Thermal correction to Energy=	0.666902
Thermal correction to Enthalpy=	0.667846
Thermal correction to Gibbs Free Energy=	0.547219
Sum of electronic and zero-point Energies=	-2496.501729
Sum of electronic and thermal Energies=	-2496.459770
Sum of electronic and thermal Enthalpies=	-2496.458826
Sum of electronic and thermal Free Energies=	-2496.579453

cartesian

12	0.32792419	0.15321603	-0.32939997	1	-1.56357586	-2.55158401	-2.89409995
8	2.39532423	-2.65828395	2.48600006	1	-1.39867580	-0.94308400	-2.17269993
8	-1.50977576	0.15041603	-0.04820000	1	4.07962418	-3.88158393	2.27340007
6	3.47182417	-3.42458391	3.05820012	8	1.21752417	2.00781608	-0.10550000
1	3.00002432	-4.19928408	3.66090012	15	2.55792427	2.29231596	-0.71880001
1	4.09292412	-2.78638411	3.69070005	8	2.63482428	2.27521610	-2.28889990
6	-2.80837584	-0.01448397	0.15940000	8	1.37912416	-0.30178395	-1.85080004

6	-3.55267572	0.99111599	0.85659999	6	1.60152423	1.70561600	-3.15910006
6	-4.92207575	0.79411602	1.05550003	6	1.60942423	0.18651603	-3.11529994
1	-5.50477600	1.54371595	1.58089995	1	1.84832418	2.09611607	-4.14919996
6	-5.57787609	-0.34428397	0.59869999	1	0.63342416	2.10621595	-2.84189987
6	-4.84917593	-1.31838393	-0.07550000	1	0.84822416	-0.13398397	-3.85389996
1	-5.37657595	-2.19988394	-0.42499998	1	2.58452415	-0.15688396	-3.50749993
6	-3.47687578	-1.19078398	-0.31039998	8	1.38902426	-0.83308399	1.09669995
6	-2.87497568	2.27051592	1.38090003	15	2.67802429	-1.48658395	1.45280004
6	-2.72037601	-2.30428410	-1.05859995	8	3.72052431	-0.53638399	2.19610000
6	-2.27767563	3.07681608	0.20999999	8	3.76062441	1.36761606	-0.23480000
1	-1.54617584	2.49161601	-0.34759998	6	3.61462402	0.90161604	2.19140005
1	-1.78447592	3.98381591	0.58350003	6	4.38662434	1.53541601	1.05349994
1	-3.06927586	3.38371611	-0.48280001	1	4.06392431	1.22141600	3.13490009
6	-1.78247583	1.91681600	2.41059995	1	2.56322432	1.19961596	2.18420005
1	-1.03277576	1.24631596	1.98940003	1	4.52302408	2.60061598	1.26110005
1	-2.22597599	1.41851604	3.27979994	1	5.36592388	1.06441605	0.95530003
1	-1.27767575	2.82611609	2.76239991	8	3.55542421	-2.08388400	0.28460002
6	-3.86347580	3.20691609	2.09829998	6	2.94192410	-2.86038399	-0.80320001
1	-4.32147598	2.73521590	2.97429991	1	2.27312422	-2.18258405	-1.34309995
1	-4.66397572	3.55251598	1.43519998	6	4.06262398	-3.37028408	-1.67799997
1	-3.32647562	4.09531593	2.45050001	1	2.37352419	-3.68408394	-0.35889998
6	-3.64277601	-3.46428394	-1.47659993	1	-6.64487600	-0.47118399	0.76660001
1	-4.43017578	-3.14008403	-2.16549993	8	3.09002399	3.73811603	-0.29439998
1	-4.11527586	-3.95138407	-0.61680001	6	2.21732426	4.87691593	-0.44679999
1	-3.04867601	-4.22528410	-1.99559999	1	2.71562433	5.70821619	0.05060000
6	-1.63917577	-2.91938400	-0.14690000	1	2.08842421	5.10631609	-1.50740004
1	-0.95567584	-2.16768408	0.25020000	1	1.25082421	4.68161583	0.02230000
1	-1.05347586	-3.66908407	-0.69449997	1	3.63502407	-3.94048405	-2.50810003
1	-2.10547590	-3.41658401	0.71090001	1	4.63502407	-2.53758407	-2.09389997
6	-2.09967566	-1.75688398	-2.36019993	1	4.74272394	-4.02448416	-1.12500000
1	-2.88667583	-1.37728393	-3.02149987				

MI-4m

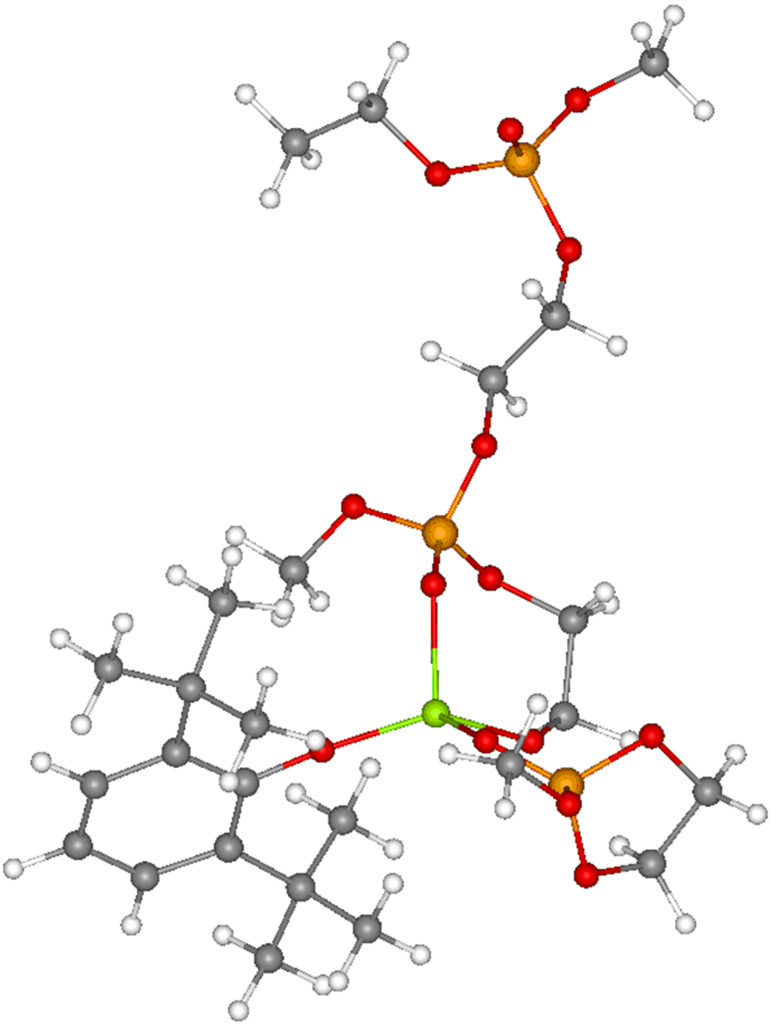

Zero-point vibrational energy	1946318.9 (Joules/Mol)
	465.18137 (Kcal/Mol)
Zero-point correction=	0.741314 (Hartree/Particle)
Thermal correction to Energy=	0.794161
Thermal correction to Enthalpy=	0.795105
Thermal correction to Gibbs Free Energy=	0.644976
Sum of electronic and zero-point Energies=	-3257.067540
Sum of electronic and thermal Energies=	-3257.014693
Sum of electronic and thermal Enthalpies=	-3257.013749
Sum of electronic and thermal Free Energies=	-3257.163878

cartesian

12	-1.61996853	0.97902334	-0.00587332	1	-0.77956855	1.40142334	-3.37387323
8	-2.48966837	-0.67927670	0.14692667	1	-0.67526853	3.14852333	-3.15357327
6	-3.17336845	-1.81527662	0.15302669	8	1.79513144	-1.26977670	-0.54267329

6	-3.89266825	-2.23897672	-1.01307321	6	0.80103147	-2.18807673	-1.07017326
6	-4.53546858	-3.48057675	-0.98907334	1	0.68843150	-2.02297664	-2.14287329
1	-5.07386827	-3.82587647	-1.86537337	1	-0.15296853	-2.05807662	-0.55667329
6	-4.51686859	-4.30187702	0.13272668	1	1.19373155	-3.18537664	-0.87987334
6	-3.86316824	-3.86187673	1.27862668	8	-2.39636850	2.32942319	1.29742670
1	-3.88256836	-4.50277710	2.15392685	15	-2.90546846	3.71762323	1.08962679
6	-3.19756842	-2.63317657	1.33102679	8	-1.78506863	4.77142286	0.63772666
6	-3.99586821	-1.35367668	-2.26977324	8	-3.95876837	3.96832323	-0.08177331
6	-2.53566837	-2.18087673	2.64692664	6	-2.19856834	5.47262287	-0.55767334
6	-4.66296864	-0.00927672	-1.91527343	6	-3.28306866	4.60332298	-1.20027328
1	-4.13076830	0.51502329	-1.12137341	1	-2.57086849	6.45662308	-0.25927332
1	-4.69886827	0.64712328	-2.79337335	1	-1.32286859	5.57932329	-1.19747329
1	-5.69006824	-0.17667672	-1.57287335	1	-2.85096836	3.81212330	-1.81797338
6	-2.60746861	-1.12457669	-2.89817333	1	-4.03076839	5.18632317	-1.73807335
1	-1.92646849	-0.62827671	-2.20647335	8	-3.63046837	4.26522303	2.38112664
1	-2.16206837	-2.08177662	-3.19387317	6	-3.51136875	3.61352324	3.66882682
1	-2.69096851	-0.49927673	-3.79567337	1	-2.64116859	4.01382303	4.19222689
6	-4.86566830	-1.99087667	-3.36877322	1	-3.41866875	2.53462338	3.54582667
1	-4.44876862	-2.93487668	-3.73607326	1	-4.42286825	3.86002326	4.21122694
1	-5.89106846	-2.17527676	-3.03127337	8	2.81313133	0.87982333	0.18682668
1	-4.92176819	-1.30627668	-4.22287321	6	4.10053158	0.58402330	-0.39677331
6	-2.74146843	-3.19597673	3.78552675	1	4.06323147	0.70482326	-1.48307323
1	-3.80056858	-3.36237669	4.00902653	1	4.37693167	-0.44527671	-0.15857331
1	-2.27896833	-4.16467714	3.56672668	6	5.07613182	1.57432330	0.21762668
1	-2.27156830	-2.81157660	4.69802666	8	6.37403154	1.36782336	-0.36387330
6	-1.01286852	-2.02027678	2.47562671	1	4.78293180	2.59782338	-0.02557332
1	-0.76436853	-1.26817667	1.72762680	1	5.11423159	1.46782327	1.30482674
1	-0.55006856	-1.71907663	3.42402673	15	7.49903154	0.55652332	0.44972667
1	-0.56156856	-2.97267675	2.17342663	8	7.27573156	-0.93537664	-0.07367332
6	-3.16266870	-0.85417664	3.12322664	8	8.83723068	0.98142326	-0.31437331
1	-4.22796822	-0.99337673	3.33982682	8	7.48813152	0.75702327	1.91402674
1	-2.67176867	-0.51057673	4.04312658	6	9.48563099	2.21282315	0.05522668
1	-3.07186842	-0.07227671	2.36842680	6	8.03053093	-2.01497674	0.53992665
1	-5.02186823	-5.26487684	0.12062668	6	7.49363136	-3.32297659	0.00372668
8	0.33113146	0.64302325	0.47262669	1	9.08643150	-1.88367677	0.28352669
15	1.47423148	0.27292329	-0.41167331	1	7.91893148	-1.94737673	1.62572670
8	1.32503152	0.73702329	-1.92107344	1	8.90573120	3.06572342	-0.30737332
8	-1.39746857	2.11202335	-1.52627325	1	9.60713100	2.27252316	1.13902664
6	0.91953146	2.09802318	-2.29047322	1	10.46063137	2.19932318	-0.43127334
6	-0.56626856	2.18062329	-2.61827326	1	8.05293179	-4.15487671	0.44222668
1	1.17083144	2.78422332	-1.47537327	1	6.43813181	-3.44547677	0.26032668

1	1.53243148	2.33222318	-3.16547322	1	7.59763145	-3.37157679	-1.08317327

S1.3. Molecular structures, energies and cartesian coordinates for binonuclear mechanism

DI-1__THF

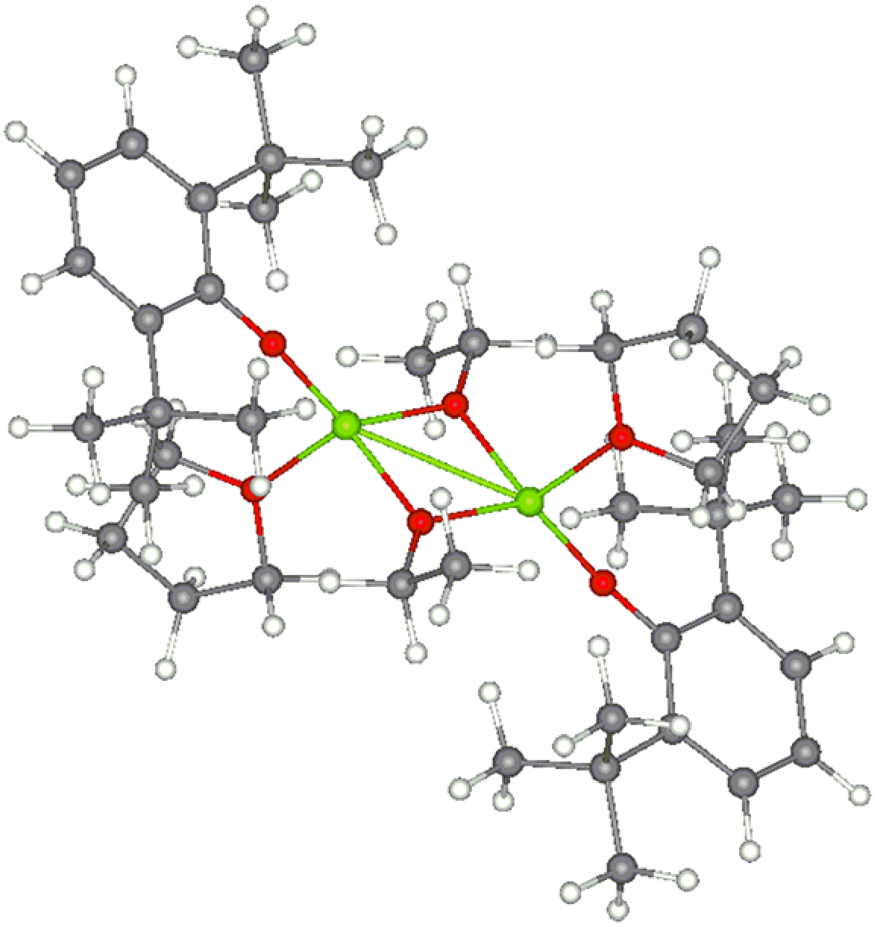

Zero-point vibrational energy	2688289.1 (Joules/Mol)
	642.51652 (Kcal/Mol)
Zero-point correction=	1.023915 (Hartree/Particle)
Thermal correction to Energy=	1.082452
Thermal correction to Enthalpy=	1.083396
Thermal correction to Gibbs Free Energy=	0.927286
Sum of electronic and zero-point Energies=	-2415.288266
Sum of electronic and thermal Energies=	-2415.229729
Sum of electronic and thermal Enthalpies=	-2415.228785
Sum of electronic and thermal Free Energies=	-2415.384895

cartesian

12	-1.42806900	-0.14603621	0.36572510	6	0.01873104	-2.29913616	-2.76147485
8	0.03603104	1.16836381	0.59032512	1	-1.03836906	-2.92593622	-1.00187492

8	-3.21136904	0.09606380	-0.16957490	6	2.83813095	1.05076373	-3.05007505
8	-1.64806902	-1.07903624	2.21852493	1	2.81733108	0.13526379	-3.65197492
6	0.09403104	2.42176366	1.24842501	1	3.70273089	1.02526379	-2.38627505
1	-0.71176893	3.06946373	0.87622511	6	2.72543097	2.29326367	-3.91567492
6	-0.01756896	2.29806376	2.76202512	1	3.07663107	3.17196369	-3.36607504
6	-2.84016895	-1.05043626	3.04942513	1	3.30763102	2.20726371	-4.83637476
1	-2.81956911	-0.13443621	3.65062499	6	1.21633101	2.37116385	-4.16427469
1	-3.70416903	-1.02513623	2.38472509	1	0.92603105	1.68256378	-4.96387482
6	-2.72876906	-2.29223633	3.91602492	1	0.87563103	3.37176371	-4.43997478
1	-3.07986903	-3.17123628	3.36682510	6	0.62823105	1.92046380	-2.82997489
1	-3.31156898	-2.20523620	4.83622551	1	0.44233105	2.75726366	-2.15177488
6	-1.21976900	-2.37063622	4.16582537	1	-0.28656894	1.33286381	-2.93047500
1	-0.92976892	-1.68133628	4.96482515	6	4.52913094	-0.22573622	0.30762509
1	-0.87986892	-3.37123632	4.44282532	6	5.16613102	-1.48223627	0.04282509
6	-0.63066894	-1.92163622	2.83152509	6	6.55943108	-1.56073630	0.12762509
1	-0.44546893	-2.75933623	2.15402508	1	7.06013107	-2.49953628	-0.08257490
1	0.28483105	-1.33503628	2.93182492	6	7.34183121	-0.46713620	0.47882509
6	-4.52836895	0.22586378	-0.30797490	6	6.71563101	0.73316383	0.79302508
6	-5.16536903	1.48226380	-0.04277491	1	7.33803082	1.56776381	1.09682500
6	-6.55866909	1.56076372	-0.12707491	6	5.32673120	0.88646382	0.73642510
1	-7.05936909	2.49946380	0.08352509	6	4.35913086	-2.74263620	-0.31887490
6	-7.34106922	0.46726379	-0.47837490	6	4.69633102	2.22036386	1.18002498
6	-6.71486902	-0.73293620	-0.79307491	6	3.65083098	-2.55313635	-1.67267501
1	-7.33736897	-1.56743622	-1.09697497	1	2.97033095	-1.70043623	-1.65077496
6	-5.32606888	-0.88613617	-0.73687488	1	3.07313108	-3.44563627	-1.94167495
6	-4.35816908	2.74266386	0.31902510	1	4.38493109	-2.37393618	-2.46607494
6	-4.69566917	-2.21993613	-1.18107498	6	3.33933091	-3.06213617	0.79282510
6	-3.64966893	2.55296373	1.67262506	1	2.65143108	-2.23233628	0.96262509
1	-2.96896911	1.70046377	1.65052509	1	3.85933089	-3.26423621	1.73572505
1	-3.07216907	3.44566369	1.94172502	1	2.75583100	-3.95453620	0.53342509
1	-4.38356924	2.37356377	2.46612501	6	5.24633121	-3.99233627	-0.46427491
6	-3.33856893	3.06236386	-0.79277492	1	5.79263115	-4.21993637	0.45702508
1	-2.65076900	2.23256373	-0.96267492	1	5.97073078	-3.90023613	-1.28047502
1	-3.85876894	3.26446366	-1.73557496	1	4.61333084	-4.85793638	-0.69047493
1	-2.75516891	3.95476365	-0.53337491	6	5.74393082	3.23266387	1.67802501
6	-5.24546909	3.99236369	0.46472511	1	6.45173120	3.52166367	0.89372510
1	-5.79196882	4.21996355	-0.45647490	1	6.31223106	2.85216379	2.53272510
1	-5.96966887	3.90016365	1.28102505	1	5.23353100	4.14506388	2.00682497
1	-4.61246920	4.85786390	0.69072509	6	3.73423100	1.96776378	2.35662508
6	-5.74326897	-3.23193622	-1.67947495	1	2.95903111	1.24866378	2.08852506
1	-6.45106888	-3.52133632	-0.89537489	1	3.25413108	2.90276384	2.67112494

1	-6.31166887	-2.85093617	-2.53397489	1	4.28363085	1.56646371	3.21532512
1	-5.23296881	-4.14423609	-2.00897503	6	3.95703101	2.90366387	0.01412509
6	-3.73346901	-1.96693623	-2.35757494	1	4.65263081	3.11866379	-0.80527490
1	-2.95826912	-1.24803627	-2.08917499	1	3.51943088	3.85596371	0.33962509
1	-3.25346899	-2.90183616	-2.67227507	1	3.15553093	2.27706385	-0.37907490
1	-4.28286886	-1.56523621	-3.21617508	1	1.03943098	2.92456365	1.00232506
6	-3.95646906	-2.90373635	-0.01547491	1	0.71283108	-3.07023621	-0.87547493
1	-4.65216923	-3.11903620	0.80392510	1	8.42443180	-0.55493617	0.52772510
1	-3.51896906	-3.85593629	-0.34127492	1	-8.42366886	0.55506384	-0.52697492
1	-3.15486908	-2.27743626	0.37802508	1	0.03763104	3.28666377	3.23242497
12	1.42843103	0.14496379	-0.36527491	1	0.79423106	1.68526375	3.16422510
8	-0.03576896	-1.16943622	-0.58967489	1	-0.97036892	1.84336376	3.05122495
8	3.21223092	-0.09583620	0.16882509	1	-0.03606896	-3.28773618	-3.23187494
8	1.64693105	1.07846379	-2.21807504	1	0.97143108	-1.84403622	-3.05037498
6	-0.09316895	-2.42283630	-1.24787498	1	-0.79316896	-1.68663621	-3.16377497

DI-1__MeOEP

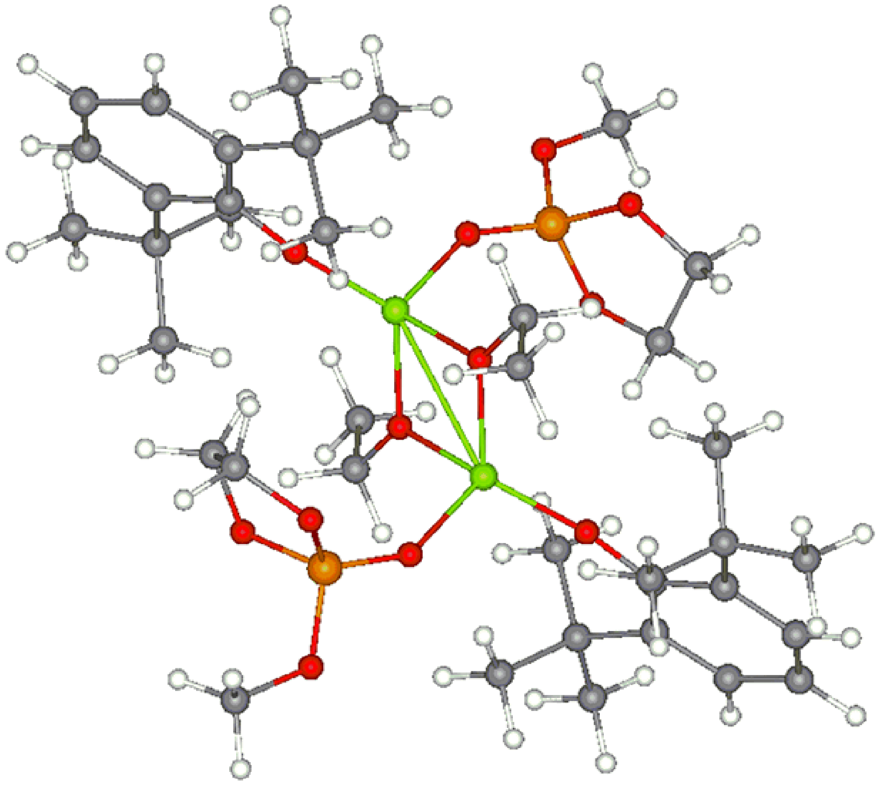

Zero-point vibrational energy	2675081.6 (Joules/Mol)
	639.35986 (Kcal/Mol)
Zero-point correction=	1.018885 (Hartree/Particle)
Thermal correction to Energy=	1.084481
Thermal correction to Enthalpy=	1.085425
Thermal correction to Gibbs Free Energy=	0.914368
Sum of electronic and zero-point Energies=	-3471.863116
Sum of electronic and thermal Energies=	-3471.797521
Sum of electronic and thermal Enthalpies=	-3471.796576
Sum of electronic and thermal Free Energies=	-3471.967633

cartesian

12	1.30913222	-0.71193415	0.02037165	6	-3.40286779	-1.04893410	-2.76862836
8	-0.01356778	-0.04133416	1.33867168	1	-2.67326784	-1.01683414	-1.95792830
8	3.15723205	-0.28443417	0.00057165	1	-3.93536782	-2.00713420	-2.72382832
6	0.00703222	-0.18383417	2.74797153	1	-2.86396790	-1.01273417	-3.72282839
6	-0.25436780	1.13066590	3.46767163	6	-5.34846783	-0.02543417	-3.88172841
1	0.98243219	-0.57623416	3.06357145	1	-5.86476803	-0.99103415	-3.91022849
6	4.48353195	-0.16973417	0.05407165	1	-6.10126781	0.76886582	-3.91462851
6	5.15363216	0.02506583	1.30797172	1	-4.75396776	0.05166584	-4.79932833

6	6.53503227	0.24236584	1.30877173	6	-5.69296789	0.59256583	3.63967156
1	7.05123234	0.40716583	2.24817157	1	-6.45906782	1.35856581	3.47997165
6	7.28723192	0.24826583	0.14047165	1	-6.19336796	-0.37173417	3.77937150
6	6.64733219	-0.00853417	-1.06562829	1	-5.18456793	0.83336586	4.58027172
1	7.24953222	-0.03903417	-1.96722829	6	-3.62816787	-0.52353418	2.89657164
6	5.26963234	-0.23393416	-1.14522827	1	-2.84006786	-0.61933416	2.14977145
6	4.40493202	-0.02913417	2.65157151	1	-3.16486788	-0.28733417	3.86207151
6	4.64633226	-0.58283418	-2.50762844	1	-4.12396765	-1.49663413	2.99437165
6	3.74173212	-1.41073418	2.81947160	6	-4.00776768	1.95346582	2.46017146
1	3.06213212	-1.64463413	1.99867165	1	-4.77016783	2.71296573	2.25497150
1	3.18223214	-1.46113420	3.76187158	1	-3.54336786	2.19726586	3.42347169
1	4.50573206	-2.19553423	2.84147167	1	-3.25156784	2.03586578	1.67807174
6	3.36593223	1.10276580	2.73927164	1	-0.73936778	-0.92753416	3.05957150
1	2.61733222	1.03336585	1.94967175	1	0.73003221	0.91096586	-3.00442839
1	3.86223221	2.07666588	2.65467167	1	-8.37666798	-0.35033417	-0.19942835
1	2.84853220	1.07836580	3.70567155	1	8.35953236	0.42566583	0.17257164
6	5.34033203	0.14926584	3.86147165	8	-1.05396771	2.72506595	0.02147165
1	5.83383226	1.12716579	3.86707163	15	-0.06146779	3.81826591	-0.11572835
1	6.11133194	-0.62673414	3.91017151	8	0.55123222	3.99366593	-1.59012830
1	4.75013208	0.07946583	4.78227186	8	1.31963229	3.69346595	0.68807161
6	5.68353224	-0.64013416	-3.64372849	6	1.99873221	3.93266582	-1.57062829
1	6.45333195	-1.39863420	-3.46762848	6	2.38493204	3.31316590	-0.22282836
1	6.17963219	0.32306585	-3.80502844	1	2.37843204	4.95176554	-1.68392825
1	5.17553234	-0.90433419	-4.57832813	1	2.31843209	3.32106590	-2.41332841
6	3.61733222	0.48626584	-2.91662836	1	2.45043206	2.22346592	-0.26042834
1	2.83643222	0.59006584	-2.16262841	1	3.31423211	3.71686578	0.17667164
1	3.14723206	0.23256584	-3.87452841	8	-0.74676782	5.16576576	0.34077165
1	4.11703205	1.45586586	-3.03512836	8	1.05843222	-2.73043418	0.11127165
6	4.00153208	-1.98103416	-2.43532848	15	0.09373222	-3.83783412	0.31507167
1	4.76523209	-2.73483419	-2.21502852	8	-0.74266779	-4.25463438	-0.99172837
1	3.53693223	-2.24513412	-3.39312840	8	-1.14436769	-3.58913422	1.30367172
1	3.24643207	-2.04523420	-1.65052831	6	-2.16866779	-4.21113443	-0.73752838
12	-1.31636775	0.70706582	0.05097165	6	-2.34806776	-3.36133409	0.52327162
8	0.00533222	0.03196584	-1.27792835	1	-2.51766777	-5.23823452	-0.59942836
8	-3.16586781	0.29086584	-0.01622835	1	-2.64946795	-3.76743412	-1.60802829
6	-0.01846778	0.17076585	-2.68612838	1	-2.43466783	-2.29503417	0.30247167
6	0.24133222	-1.14353418	-3.40672851	1	-3.19526792	-3.67723417	1.13047171
1	-0.99156779	0.56746584	-3.00202847	8	0.88283223	-5.09203434	0.86087161
6	-4.49326801	0.19376583	-0.06672835	6	0.21763222	-6.34773445	1.09967172
6	-5.16646767	0.03756583	-1.32452834	6	-0.03906778	6.42006540	0.29867166
6	-6.55066776	-0.16163416	-1.33212829	1	0.84633219	6.37866545	0.93707162

1	-7.06866789	-0.29603416	-2.27532840	1	0.23433222	6.66556549	-0.73042840
6	-7.30216789	-0.18743417	-0.16392836	1	-0.59516782	-6.21803427	1.81797171
6	-6.65846777	0.03116583	1.04787171	1	-0.15646778	-6.75983429	0.15947165
1	-7.26036787	0.04576584	1.94997168	1	-0.73506778	7.16516542	0.68017161
6	-5.27836800	0.23886584	1.13417172	1	0.97523218	-7.00993443	1.51527166
6	-4.41426802	0.10686583	-2.66502833	1	-0.22116777	0.98806584	4.55457163
6	-4.65376806	0.55456585	2.50417161	1	0.49613219	1.87826586	3.19547153
6	-3.71716785	1.47476590	-2.80382848	1	-1.24066770	1.52806580	3.21197152
1	-3.03536797	1.67536581	-1.97642827	1	0.21223222	-0.99973416	-4.49342823
1	-3.15406775	1.53096581	-3.74382854	1	1.22393227	-1.54663420	-3.14692855
1	-4.46346807	2.27656579	-2.81272840	1	-0.51176780	-1.89043415	-3.13972855

DI-1i

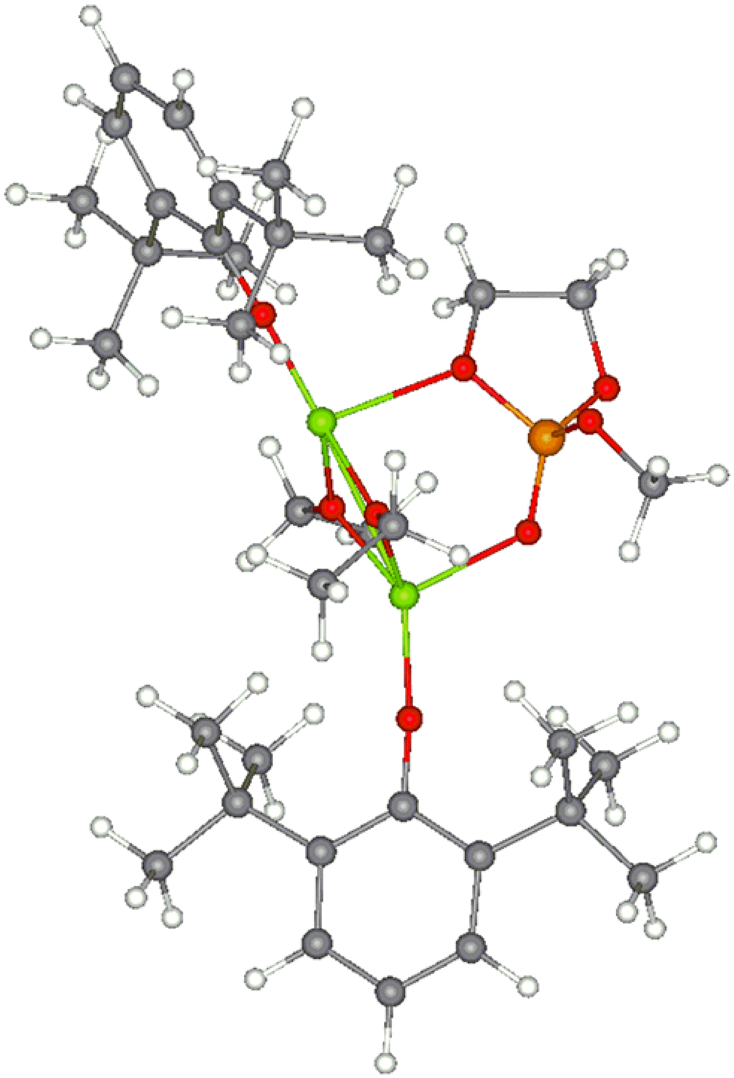

Zero-point vibrational energy	2364823.6 (Joules/Mol)
	565.20640 (Kcal/Mol)
Zero-point correction=	0.900714 (Hartree/Particle)
Thermal correction to Energy=	0.957068
Thermal correction to Enthalpy=	0.958012
Thermal correction to Gibbs Free Energy=	0.805897
Sum of electronic and zero-point Energies=	-2711.281411
Sum of electronic and thermal Energies=	-2711.225057
Sum of electronic and thermal Enthalpies=	-2711.224113
Sum of electronic and thermal Free Energies=	-2711.376228

cartesian

12	-1.53715086	-0.19066104	0.44635624	6	5.12244892	-0.28676105	-1.44454384
8	-0.17965086	-0.73596108	-0.91764373	6	4.52864885	2.30383897	1.41315627

8	-3.31445074	0.27383897	0.19815625	6	4.36644888	-1.27856112	-2.34914374
6	-0.35385084	-1.41176105	-2.15344381	6	3.41214919	3.16133904	0.78435624
1	-1.09515083	-2.21546102	-2.03004384	1	2.65494919	2.54983902	0.29105625
1	0.58754909	-1.89786112	-2.44104385	1	2.91724920	3.77403903	1.54755616
6	-4.52105093	0.66743898	-0.20584375	1	3.83174920	3.83653927	0.03125625
6	-5.57645082	-0.29016104	-0.33964375	6	3.97184920	1.42313898	2.54805613
6	-6.82755089	0.14883897	-0.78334373	1	3.23244929	0.71573901	2.16875625
1	-7.64025116	-0.56056106	-0.89674371	1	4.78014898	0.85033894	3.01715612
6	-7.07565117	1.48133898	-1.09064364	1	3.50144935	2.03923893	3.32415628
6	-6.05185080	2.40933895	-0.94064373	6	5.51264906	3.28713894	2.07165623
1	-6.26545095	3.44653916	-1.17494369	1	6.32874918	2.77493906	2.59225631
6	-4.77585077	2.04543900	-0.49944377	1	5.94854879	3.98363924	1.34815621
6	-5.36645079	-1.77966106	-0.01074374	1	4.97704887	3.88613892	2.81665611
6	-3.69885087	3.13143897	-0.31864375	6	5.28444910	-1.94336104	-3.39094377
6	-4.94545078	-1.95436108	1.46215630	1	5.74324894	-1.21376109	-4.06584358
1	-4.03635120	-1.40006101	1.69405627	1	6.08204889	-2.53276086	-2.92654371
1	-4.77195120	-3.01426101	1.68875623	1	4.69204903	-2.62826085	-4.00814390
1	-5.73875093	-1.59566104	2.12695622	6	3.75484920	-2.42566085	-1.52154374
6	-4.31245089	-2.38796091	-0.95614380	1	3.05824924	-2.06076097	-0.76604372
1	-3.34965086	-1.88276112	-0.86094373	1	3.21744919	-3.12696099	-2.17194390
1	-4.63375092	-2.29776096	-1.99944377	1	4.54464912	-2.98556089	-1.00744367
1	-4.17565107	-3.45546103	-0.73814374	6	3.27674913	-0.53326106	-3.14514375
6	-6.64785099	-2.61146092	-0.20064375	1	3.73084927	0.22183895	-3.79524374
1	-7.00515079	-2.59866095	-1.23584366	1	2.71364927	-1.22906113	-3.77864385
1	-7.46115112	-2.27136087	0.44865626	1	2.57264924	-0.01586103	-2.49064374
1	-6.44105101	-3.65616107	0.05925625	6	-0.81225085	-0.47856104	-3.26314378
6	-4.21645117	4.53983927	-0.66474372	1	-0.40685084	2.63773894	2.00075626
1	-5.05685091	4.84073925	-0.03064375	1	8.28434944	0.96573889	-1.06494379
1	-4.52645111	4.62363911	-1.71184373	1	-8.05775070	1.79433894	-1.43654370
1	-3.41195083	5.26663923	-0.50514376	8	-1.25385094	-1.99466109	1.50055623
6	-2.49695086	2.87773895	-1.24914384	15	0.01494915	-2.76476097	1.51195621
1	-2.04225087	1.90003884	-1.08334374	8	0.37964916	-3.36796093	2.95295620
1	-1.72415090	3.64173913	-1.09974384	8	1.40494907	-1.96146107	1.26355624
1	-2.81105065	2.91653895	-2.29784369	6	1.81864917	-3.41746092	3.08755612
6	-3.25315070	3.18333912	1.15545630	6	2.32724929	-2.16396093	2.37945628
1	-4.09885120	3.45003915	1.79855633	1	2.19124913	-4.33436108	2.62245607
1	-2.47075081	3.93933916	1.29595613	1	2.04864931	-3.41826105	4.15235615
1	-2.87325072	2.21873903	1.49435616	1	2.27114916	-1.28076112	3.01685619
12	1.34864914	0.00073896	0.04135625	1	3.32634926	-2.26916099	1.95945621
8	0.01224915	0.76453900	1.24505615	8	0.10874915	-3.92876101	0.44905624
8	3.16774917	0.25973895	-0.16424376	6	-1.05735087	-4.71406078	0.09075625

6	0.05474915	1.69343889	2.31655622	1	-1.27355087	-5.42966080	0.88685626
6	-0.65445089	1.17413890	3.55945611	1	-1.91375089	-4.06316090	-0.08424374
1	1.09864914	1.92273891	2.56345630	1	-0.78675079	-5.23886108	-0.82344377
6	4.46224880	0.46213898	-0.41944373	1	-0.93795085	-1.03156102	-4.20094395
6	5.19844913	1.42733896	0.33815625	1	-1.77015090	-0.01446104	-3.01054382
6	6.56464911	1.57773888	0.08155625	1	-0.07885085	0.31603897	-3.43234372
1	7.14334917	2.29913902	0.64785624	1	-0.60375082	1.91363895	4.36635637
6	7.22024918	0.82963896	-0.88874376	1	-1.71325088	0.97433889	3.36125612
6	6.49204922	-0.08326104	-1.64204383	1	-0.19765085	0.24553895	3.91815615
1	7.01514912	-0.64526105	-2.40784383				

DI-1ic

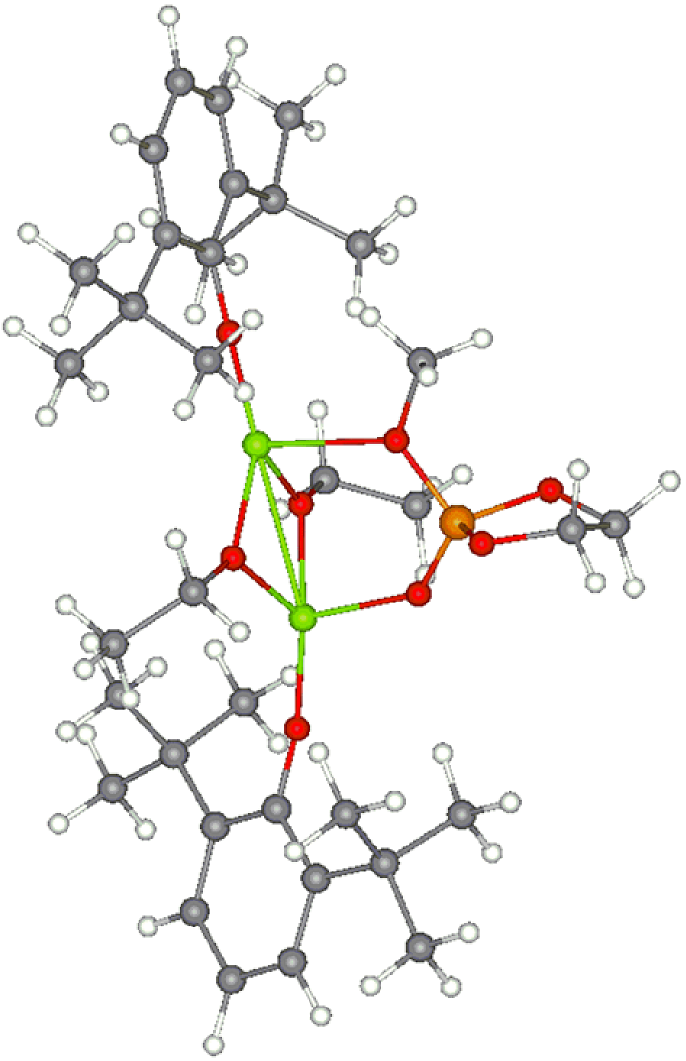

Zero-point vibrational energy	2366047.4 (Joules/Mol)
	565.49889 (Kcal/Mol)
Zero-point correction=	0.901180 (Hartree/Particle)
Thermal correction to Energy=	0.957280
Thermal correction to Enthalpy=	0.958224
Thermal correction to Gibbs Free Energy=	0.807264
Sum of electronic and zero-point Energies=	-2711.280121
Sum of electronic and thermal Energies=	-2711.224021
Sum of electronic and thermal Enthalpies=	-2711.223077
Sum of electronic and thermal Free Energies=	-2711.374037

cartesian

12	1.52467597	0.06705249	0.21460950	6	-5.11182404	-0.38324749	-1.56039047
8	0.17317595	0.01075250	-1.24539042	6	-4.61352396	-1.34754753	2.18950939
8	3.34497595	-0.27924752	0.20350951	6	-4.30862427	-0.12024750	-2.84899068

6	0.33267596	0.30885249	-2.62329054	6	-3.70272422	-2.57654738	1.99640954
1	0.87357593	1.26075244	-2.73409057	1	-2.91302419	-2.39134741	1.26590955
1	-0.65252405	0.45345247	-3.08449054	1	-3.23452425	-2.86284733	2.94590950
6	4.59787607	-0.61624748	-0.09199050	1	-4.28762436	-3.43104744	1.64010954
6	5.53377581	0.38925248	-0.49429053	6	-3.82002425	-0.17344750	2.79490948
6	6.83617592	-0.00214751	-0.81969053	1	-3.00032425	0.14375249	2.14830947
1	7.56147575	0.74115252	-1.13349044	1	-4.47652435	0.68885249	2.95910954
6	7.24637604	-1.32894754	-0.75409049	1	-3.39312410	-0.45714748	3.76440954
6	6.33897591	-2.29634738	-0.33739051	6	-5.66132402	-1.74434757	3.24560952
1	6.67997599	-3.32444739	-0.27849051	1	-6.33592415	-0.91804749	3.49470949
6	5.02067566	-1.98044765	0.00490950	1	-6.26612425	-2.59974742	2.92830944
6	5.14297581	1.87765241	-0.56529051	1	-5.14892435	-2.03424740	4.16970968
6	4.07157564	-3.09154749	0.49240950	6	-5.21332407	0.01195249	-4.08779049
6	4.67717600	2.37255263	0.81910950	1	-5.80782413	-0.89044744	-4.26329041
1	3.83407593	1.79235244	1.19370949	1	-5.89482403	0.86665249	-4.01969051
1	4.37787580	3.42765260	0.76690948	1	-4.58782434	0.16625249	-4.97399044
1	5.49467564	2.29025269	1.54400957	6	-3.52422404	1.20175242	-2.74579048
6	4.04457569	2.09685254	-1.62449050	1	-2.79482412	1.17825246	-1.93449044
1	3.15737581	1.49685240	-1.41969049	1	-2.98552418	1.40635240	-3.67889047
1	4.41157579	1.81775248	-2.61809063	1	-4.20862436	2.03835249	-2.56399059
1	3.74297595	3.15125251	-1.65769041	6	-3.35692406	-1.30004752	-3.13149047
6	6.32347584	2.77585268	-0.97629052	1	-3.92992425	-2.21874738	-3.29549050
1	6.70217609	2.53525257	-1.97539055	1	-2.76242423	-1.10914755	-4.03279018
1	7.15627575	2.71345258	-0.26789051	1	-2.67342424	-1.48454750	-2.30099058
1	5.99097586	3.82025266	-0.99779052	6	1.08127594	-0.78944749	-3.36279058
6	4.75987577	-4.46694756	0.55940950	1	0.57927591	-2.18714738	2.58670950
1	5.61097574	-4.47184753	1.24840951	1	-8.36662388	-0.72994751	-0.56499052
1	5.10797596	-4.80604744	-0.42199051	1	8.26487541	-1.60464752	-1.01649046
1	4.04167604	-5.21054745	0.92340946	8	1.09737599	2.05625248	0.70340949
6	2.87877584	-3.25544739	-0.46979052	15	-0.10492406	2.90775251	0.81180948
1	2.31277585	-2.33054733	-0.58859050	8	-0.12342405	4.18545246	-0.15859050
1	2.19177580	-4.02944756	-0.10609050	8	-0.33032405	3.68285251	2.20020938
1	3.22717595	-3.55404735	-1.46439040	6	-0.26242405	5.41575241	0.58820951
6	3.58357596	-2.77824736	1.92070961	6	0.07187594	5.06345272	2.04200959
1	4.43057585	-2.75594735	2.61520934	1	-1.29042399	5.76835251	0.47380948
1	2.88437581	-3.54984736	2.26650953	1	0.42847595	6.14405251	0.16290951
1	3.08847594	-1.80784750	1.97240961	1	1.14157593	5.14135265	2.25170946
12	-1.31592405	-0.28834748	-0.03959050	1	-0.49222404	5.65485239	2.76250935
8	0.03207594	-0.66944748	1.30520952	8	-1.43102407	2.05145240	0.55740947
8	-3.14812422	-0.48784751	-0.19089049	6	-2.74202418	2.70165253	0.59600949
6	0.02657594	-1.23834753	2.60370946	1	-2.89852405	3.13125253	1.58600950

6	0.63997591	-0.31294751	3.64520955	1	-2.78802419	3.45945263	-0.18739049
1	-1.00352407	-1.47874761	2.89550948	1	-3.46252418	1.91085243	0.40060949
6	-4.47272396	-0.60904747	-0.30029050	1	1.18637598	-0.53414750	-4.42349052
6	-5.25702429	-0.95814747	0.84420949	1	2.08387589	-0.93034750	-2.94789052
6	-6.64932394	-0.98304754	0.71230948	1	0.54397595	-1.74004757	-3.28959060
1	-7.26832390	-1.22934759	1.56790960	1	0.63597590	-0.79174751	4.63090944
6	-7.28192425	-0.70744747	-0.49409050	1	1.67967594	-0.07164751	3.39840937
6	-6.50842428	-0.42984748	-1.61489046	1	0.07877594	0.62425250	3.71930933
1	-7.01802397	-0.24984752	-2.55499053				

DTS-12i

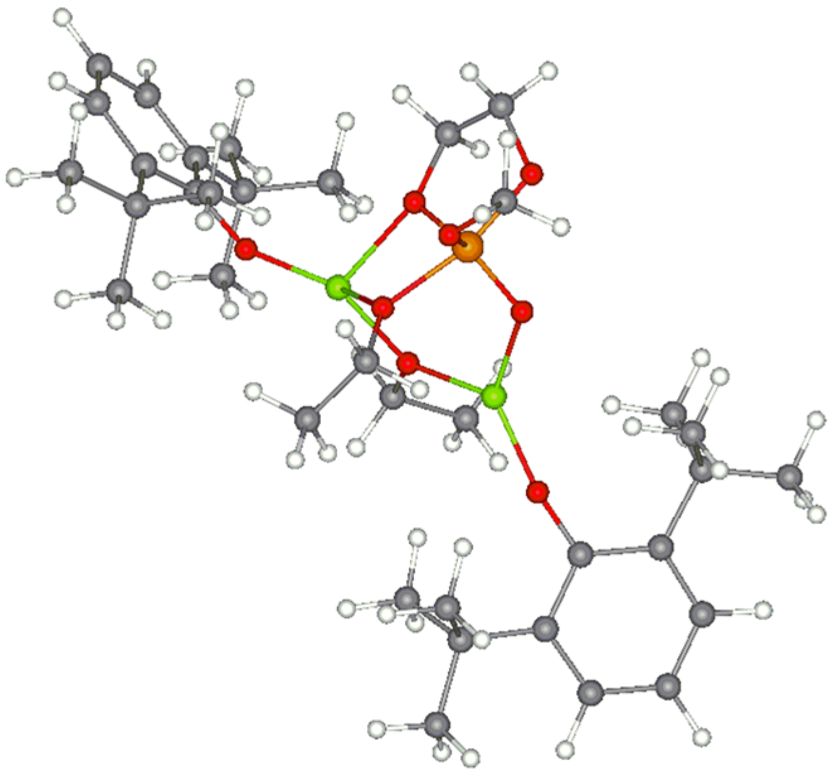

Zero-point vibrational energy	2366483.8 (Joules/Mol)
	565.60320 (Kcal/Mol)
Zero-point correction=	0.901346 (Hartree/Particle)
Thermal correction to Energy=	0.956081
Thermal correction to Enthalpy=	0.957025
Thermal correction to Gibbs Free Energy=	0.811222
Sum of electronic and zero-point Energies=	-2711.264133
Sum of electronic and thermal Energies=	-2711.209397
Sum of electronic and thermal Enthalpies=	-2711.208453
Sum of electronic and thermal Free Energies=	-2711.354256

cartesian

12	1.75231040	0.47863626	0.28072289	6	-5.41218948	-0.29436374	-0.87317711
8	-0.57038957	1.40593624	-1.21627712	6	-4.15588951	-1.87606382	2.46162271
8	3.46151042	-0.04436372	-0.14227712	6	-4.91578913	0.10103627	-2.27727723
6	-0.04518957	1.38343620	-2.55817723	6	-3.42378950	-3.08866382	1.85122287
1	0.99171042	1.73903620	-2.54127717	1	-2.84558964	-2.81056380	0.96902287
1	-0.62808961	2.07803631	-3.16717720	1	-2.75088954	-3.54506373	2.58832288
6	4.72651052	-0.46066374	-0.05547711	1	-4.14968920	-3.85016394	1.54692292
6	5.62621069	0.14513627	0.87572289	6	-3.15218949	-0.86546373	3.04952288
6	6.92291069	-0.37016374	0.97422290	1	-2.48698950	-0.45606375	2.28612280

1	7.62271070	0.06103627	1.68192291	1	-3.68078971	-0.02736373	3.51682281
6	7.35921049	-1.42626369	0.18282288	1	-2.52708960	-1.33976376	3.81572270
6	6.49321079	-1.96936369	-0.75977713	6	-4.96518946	-2.41196370	3.65692282
1	6.86071062	-2.77316380	-1.38847709	1	-5.48438931	-1.61706376	4.20302296
6	5.18301058	-1.50696373	-0.91497707	1	-5.70418930	-3.15886378	3.35012269
6	5.21791077	1.35833621	1.73412287	1	-4.28428936	-2.89986372	4.36352301
6	4.28351068	-2.10226369	-2.01497722	6	-6.07358932	0.39373627	-3.24937725
6	4.10191059	0.98553628	2.72952271	1	-6.74448919	-0.46416372	-3.36037731
1	4.44841051	0.21903628	3.43062282	1	-6.66908932	1.26113629	-2.94537711
1	3.21881032	0.57313627	2.23392272	1	-5.66168928	0.61753625	-4.23987722
1	3.78341031	1.86123621	3.30672288	6	-4.07048941	1.38693631	-2.21547723
6	4.78131056	2.52853632	0.82822287	1	-3.19548965	1.28353631	-1.57307708
1	3.96961045	2.25513625	0.15402290	1	-3.71838951	1.66793621	-3.21587729
1	5.62491083	2.85953617	0.21292290	1	-4.66648912	2.21863627	-1.82407713
1	4.44871044	3.38003635	1.43352294	6	-4.11318922	-1.05816376	-2.90277719
6	6.38191080	1.88863623	2.59192276	1	-4.75848913	-1.93136382	-3.04727721
1	7.23161077	2.20563626	1.97902286	1	-3.72278953	-0.76656371	-3.88597727
1	6.73691082	1.15113628	3.31952286	1	-3.28138971	-1.36786377	-2.26827717
1	6.04261065	2.76453614	3.15622282	6	-0.12528957	-0.02526373	-3.11237717
6	5.01021051	-3.17306376	-2.84807730	1	0.44531044	-2.68976378	0.51752287
1	5.31401062	-4.03476381	-2.24417710	1	-8.36318970	-0.56996375	0.83802289
1	5.89771080	-2.77486372	-3.35067725	1	8.37121010	-1.80996370	0.28622290
1	4.33451080	-3.54446363	-3.62667727	8	1.19021046	2.33393621	0.22612289
6	3.85551047	-0.99316370	-2.99797726	15	-0.23178956	2.80253625	-0.09297711
1	3.33201051	-0.18406372	-2.48517728	8	-0.31308958	4.04283619	1.04792285
1	3.19811034	-1.40286374	-3.77537727	8	-1.53658962	2.03883624	0.70112288
1	4.73241043	-0.56226373	-3.49277711	6	-1.55978954	4.13223648	1.70732296
6	3.04421043	-2.78826380	-1.40857708	6	-1.98488963	2.68753624	1.90572286
1	3.34191036	-3.59306383	-0.72767711	1	-2.29528952	4.67903614	1.10042286
1	2.42411041	-3.22846365	-2.19937730	1	-1.41858959	4.65503645	2.65542269
1	2.43161035	-2.08286381	-0.84547710	1	-1.48088956	2.22813630	2.76002288
12	-1.52888954	0.06923628	-0.03017711	1	-3.06358957	2.54503632	1.97562289
8	0.18191044	-0.63746375	0.65862292	8	-0.46798956	3.75763631	-1.37417710
8	-3.19758964	-0.73576373	-0.04947711	6	-0.10918957	5.15203619	-1.37267709
6	0.28071043	-1.92456365	1.28712285	1	-0.82418960	5.73713636	-0.79257709
6	1.39791036	-1.97996378	2.31382275	1	0.89571041	5.29903603	-0.97387713
1	-0.67258960	-2.15796375	1.77362287	1	-0.14268957	5.45683622	-2.41947722
6	-4.52058935	-0.74016374	0.15292290	1	0.27571043	-0.03946371	-4.13047743
6	-5.05068922	-1.22246373	1.39112294	1	0.46191043	-0.73376375	-2.51967716
6	-6.42988920	-1.13516378	1.60562289	1	-1.16118956	-0.37426376	-3.16047716
1	-6.85288954	-1.47616374	2.54432273	1	1.43261039	-2.96886373	2.78332281

6	-7.29548931	-0.63056374	0.64252287	1	2.37761045	-1.80766368	1.85382295
6	-6.78028917	-0.23926373	-0.58747709	1	1.24101043	-1.23866379	3.10502267
1	-7.47438955	0.11263628	-1.34287715				

DTS-12

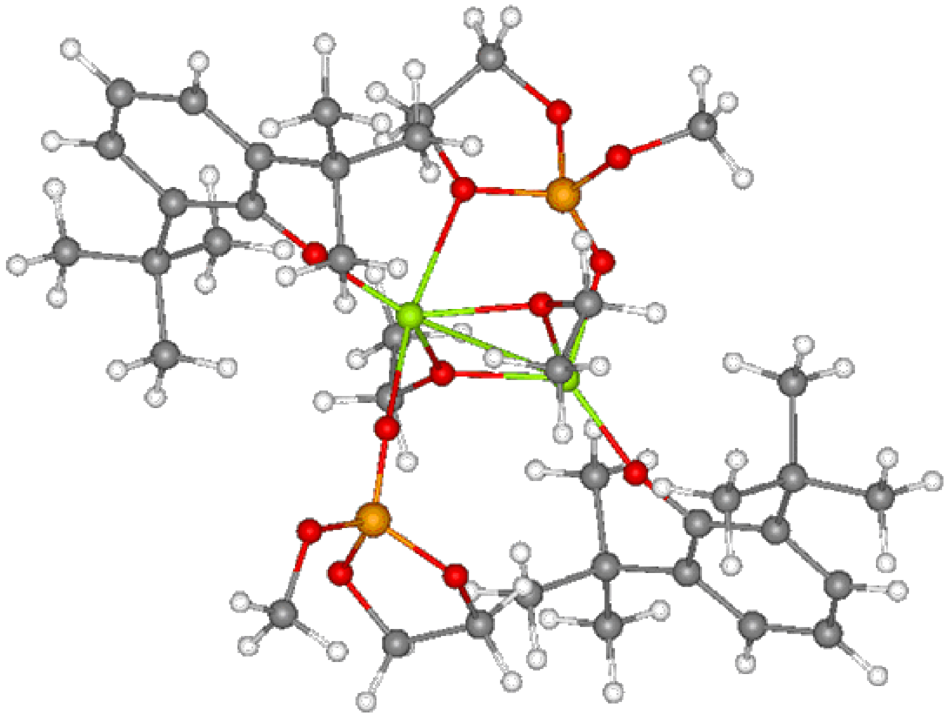

Zero-point vibrational energy	2677084.1 (Joules/Mol)
	639.83845 (Kcal/Mol)
Zero-point correction=	1.019647 (Hartree/Particle)
Thermal correction to Energy=	1.083697
Thermal correction to Enthalpy=	1.084641
Thermal correction to Gibbs Free Energy=	0.919498
Sum of electronic and zero-point Energies=	-3471.846660
Sum of electronic and thermal Energies=	-3471.782610
Sum of electronic and thermal Enthalpies=	-3471.781666
Sum of electronic and thermal Free Energies=	-3471.946809

cartesian

12	1.60025001	-0.71859998	-0.50744498	6	-3.55065012	0.71190000	-3.06664491
8	0.07594994	-1.36530006	0.74805504	1	-2.80334997	0.22669999	-2.43764496
8	3.35995007	-0.15270001	-0.17334498	1	-4.11055040	-0.06760000	-3.59644508
6	0.19584994	-1.76270008	2.12475514	1	-3.02915001	1.31949997	-3.81614494
1	1.15915000	-2.26819992	2.26185513	6	-5.46665049	2.25790000	-3.21344495
1	-0.58395004	-2.49709988	2.34405494	1	-6.02115011	1.53569996	-3.82234502
6	4.69434977	-0.09650001	-0.14614499	1	-6.18835020	2.90720010	-2.70684505
6	5.42674971	-0.69739997	0.92905504	1	-4.88975048	2.88590002	-3.90204501
6	6.82044983	-0.58529997	0.94025505	6	-5.65985012	-1.68700004	3.20675492
1	7.38914967	-1.03240001	1.74825501	1	-6.38965034	-0.93330002	3.51965499

6	7.51794958	0.08250000	-0.05854498	1	-6.20415020	-2.55110002	2.81055498
6	6.80624962	0.65079999	-1.10754502	1	-5.13015032	-2.02279997	4.10585499
1	7.36424971	1.16049993	-1.88524497	6	-3.67175007	-2.28189993	1.87965500
6	5.41174984	0.58039999	-1.18564498	1	-2.90055013	-1.98420000	1.17025506
6	4.72814989	-1.47210002	2.06015515	1	-3.18304992	-2.64339995	2.79335499
6	4.69504976	1.22049999	-2.38774490	1	-4.22045040	-3.12500000	1.44295502
6	4.00664949	-2.70609999	1.48305500	6	-3.90195012	0.02209999	2.92015505
1	3.27985001	-2.44039989	0.71365505	1	-4.62135029	0.77990001	3.24965501
1	3.48804998	-3.25689983	2.27835512	1	-3.37844992	-0.35189998	3.80935502
1	4.73064995	-3.38569999	1.02075505	1	-3.18155003	0.51709998	2.26745510
6	3.74705005	-0.55199999	2.80945492	6	0.07944994	-0.60600001	3.10105515
1	3.01075006	-0.11850001	2.13235497	1	1.01424992	1.74909997	-2.56304502
1	4.29064989	0.27130002	3.28665495	1	-8.43485069	-0.24110001	-0.41294497
1	3.21784997	-1.10340011	3.59605503	1	8.60225010	0.15329999	-0.02334498
6	5.71364975	-2.00440001	3.11635494	8	-0.96205008	1.67379999	1.13375509
1	6.26694965	-1.19860005	3.61005497	15	-0.40345007	2.97900009	1.54045498
1	6.43604994	-2.71169996	2.69575500	8	0.11664994	3.91720009	0.34365499
1	5.15274954	-2.53679991	3.89265513	8	0.95424998	2.95609999	2.40485501
6	5.66644955	1.90950000	-3.36384487	6	1.50335002	4.28749990	0.52535504
1	6.38634968	1.20919991	-3.79964495	6	2.07404995	3.30719995	1.55575502
1	6.22274971	2.72560000	-2.89044499	1	1.53304994	5.32189989	0.87885505
1	5.09444952	2.34179997	-4.19244480	1	1.99994993	4.21049976	-0.44114500
6	3.72304988	2.31380010	-1.90524495	1	2.47014999	2.39960003	1.09425509
1	2.96495008	1.89879990	-1.23904502	1	2.83704996	3.75850010	2.18945503
1	3.22215009	2.79150009	-2.75644493	8	-1.47654998	3.75890017	2.39595509
1	4.27784967	3.08850002	-1.36194491	8	1.21504998	-2.56130004	-1.21224499
6	3.95724988	0.14139999	-3.20244503	15	-0.14275005	-3.02810001	-0.75194496
1	4.67324972	-0.57639998	-3.61574507	8	-0.57755005	-4.08440018	-1.93964493
1	3.40695000	0.59000003	-4.03794479	8	-1.52645004	-2.16689992	-0.86344498
1	3.25274992	-0.42510000	-2.59164500	6	-2.00075006	-4.14800024	-2.04164505
12	-1.32365000	-0.02120000	-0.01514498	6	-2.46165013	-2.71239996	-1.83414495
8	0.15784994	0.35280001	-1.30584502	1	-2.40884995	-4.81830025	-1.27614498
8	-3.21475005	0.09269999	-0.05604498	1	-2.25565004	-4.52730036	-3.03224492
6	0.12834994	1.10289991	-2.50764489	1	-2.37755013	-2.12019992	-2.74634504
6	0.08144994	0.21859999	-3.74694490	1	-3.45915008	-2.61229992	-1.40774500
1	-0.74135005	1.77379990	-2.50254488	8	-0.23415007	-3.97399998	0.53405505
6	-4.53945017	0.06999999	-0.10574498	6	0.59934998	-5.15230036	0.57365501
6	-5.23845005	0.71450001	-1.18134499	6	-1.22375000	5.08220005	2.90525508
6	-6.62695026	0.57270002	-1.26354492	1	-0.36085007	5.07340002	3.57525492
1	-7.16755009	1.03450000	-2.08294487	1	-1.06895006	5.78160000	2.08005500
6	-7.35635042	-0.13990000	-0.31854498	1	0.38074994	-5.80250025	-0.27664500

6	-6.68655014	-0.68650001	0.77035505	1	1.65474999	-4.87170029	0.57055503
1	-7.27325010	-1.19850004	1.52585506	1	-2.11855006	5.36179972	3.45875502
6	-5.30025005	-0.58520001	0.92105502	1	0.35124993	-5.65660000	1.50705504
6	-4.50395012	1.57690001	-2.22394490	1	0.22894993	-0.98610002	4.11835480
6	-4.63755035	-1.12690008	2.20115495	1	0.82964993	0.16710000	2.92055511
6	-3.72815013	2.70880008	-1.52004492	1	-0.90935004	-0.14330001	3.06115508
1	-3.02815008	2.31980014	-0.77964497	1	0.09354994	0.82999998	-4.65684509
1	-3.17135000	3.30739999	-2.25204492	1	0.94004989	-0.45879999	-3.77934504
1	-4.42445040	3.37680006	-1.00084496	1	-0.82835007	-0.38859999	-3.76204491

DTS-12r

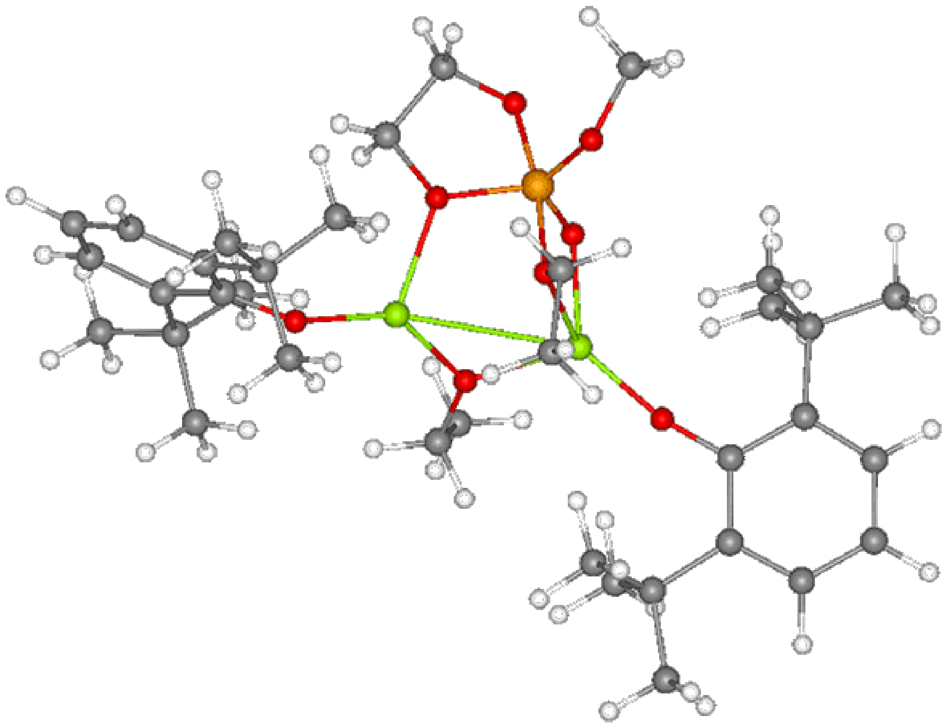

Zero-point vibrational energy	2366006.0 (Joules/Mol)
	565.48901 (Kcal/Mol)
Zero-point correction=	0.901164 (Hartree/Particle)
Thermal correction to Energy=	0.955850
Thermal correction to Enthalpy=	0.956794
Thermal correction to Gibbs Free Energy=	0.811183
Sum of electronic and zero-point Energies=	-2711.256473
Sum of electronic and thermal Energies=	-2711.201787
Sum of electronic and thermal Enthalpies=	-2711.200843
Sum of electronic and thermal Free Energies=	-2711.346454

cartesian

12	1.69343948	0.25357798	0.67307812	6	-5.18076038	0.03337798	-1.42072177
8	0.52263939	1.48557806	-0.56542188	6	-4.67696047	-1.81072187	1.97917819
8	3.37303948	-0.43352205	0.31177813	6	-4.40266037	0.42727798	-2.69102192
6	0.51943940	1.84217799	-1.96512175	6	-3.95816064	-3.05782199	1.42477822
1	1.34203947	2.53807807	-2.14592171	1	-3.20786047	-2.79362202	0.67737812
1	-0.41726062	2.35837793	-2.19672179	1	-3.47066045	-3.61482191	2.23457813
6	4.62153959	-0.64652205	-0.10822188	1	-4.67956066	-3.72762203	0.94547814
6	5.59563923	0.40097797	-0.06742188	6	-3.70206046	-0.94772202	2.80367827
6	6.87893963	0.13847798	-0.55812186	1	-2.82246065	-0.64562201	2.22717810
1	7.63113928	0.91977793	-0.54362190	1	-4.19416046	-0.03902203	3.16597819

6	7.23513937	-1.10372198	-1.06912184	1	-3.33296061	-1.50312197	3.67327809
6	6.29383945	-2.12662196	-1.07222188	6	-5.73236036	-2.31812191	2.97937822
1	6.59323931	-3.09552193	-1.45682180	1	-6.25816059	-1.50052202	3.48377824
6	4.99353933	-1.94022202	-0.59382188	1	-6.47546053	-2.96212196	2.49927807
6	5.27243948	1.79557800	0.50057811	1	-5.23766041	-2.91422200	3.75387812
6	4.01563931	-3.13002205	-0.56302190	6	-5.33596039	0.86857796	-3.83362174
6	4.80793953	1.68847799	1.96737814	1	-6.04676056	0.08277797	-4.10862160
1	3.95423937	1.02127802	2.08707809	1	-5.90046072	1.77477801	-3.58962178
1	4.52583933	2.67327809	2.35777807	1	-4.73536062	1.09177804	-4.72232199
1	5.61983967	1.29667807	2.58957815	6	-3.46676064	1.61797798	-2.41222191
6	4.21343946	2.49377799	-0.37402189	1	-2.74386048	1.39517796	-1.62512183
1	3.30323935	1.90117800	-0.47492188	1	-2.91036057	1.89227808	-3.31682181
1	4.60583925	2.65877795	-1.38352180	1	-4.04116058	2.49477792	-2.09302187
1	3.93873954	3.46717811	0.04917812	6	-3.60166049	-0.77822202	-3.22542191
6	6.49823952	2.72767806	0.50967813	1	-4.28026056	-1.58832192	-3.51272178
1	6.88163948	2.92487812	-0.49692190	1	-3.02866054	-0.49042207	-4.11542177
1	7.31583929	2.32817793	1.11837816	1	-2.91266060	-1.17532194	-2.47842193
1	6.21173954	3.69307804	0.94237810	6	0.67763937	0.59157795	-2.80572176
6	4.64203930	-4.42122221	-1.11982179	1	0.75353938	-2.86182189	1.08127820
1	5.52213955	-4.73532200	-0.54932189	1	-8.39946079	-0.02602203	-0.26322189
1	4.92943954	-4.32302237	-2.17222190	1	8.23923969	-1.27672195	-1.44872177
1	3.90813947	-5.23272228	-1.05712187	8	1.13673949	1.88747799	1.69637823
6	2.76663947	-2.84902191	-1.42032182	15	0.14763939	2.64077806	0.81797814
1	2.21653938	-1.98012209	-1.05742180	8	-0.48556060	3.67177796	1.98897815
1	2.09063935	-3.71332192	-1.40942180	8	-1.43946052	1.95747805	0.63547814
1	3.05153942	-2.66032195	-2.46132183	6	-1.86256051	3.93087792	1.80977821
6	3.62113953	-3.42522192	0.89767814	6	-2.45546055	2.57757807	1.46387815
1	4.50283957	-3.72112203	1.47637820	1	-2.03736067	4.65437794	1.00027812
1	2.89683938	-4.24852228	0.94547814	1	-2.26866055	4.33247757	2.74017811
1	3.18813944	-2.54582191	1.37677813	1	-2.59666061	1.96777797	2.35957813
12	-1.51766050	-0.08782203	0.24177814	1	-3.37786055	2.62147808	0.88337809
8	0.08103940	-0.91682202	0.89937812	8	0.58963937	3.82197809	-0.19562188
8	-3.19836044	-0.63302207	-0.25052190	6	0.89163941	5.15867758	0.24507812
6	0.09713940	-2.16142201	1.60897815	1	-0.02036061	5.71437788	0.47057810
6	0.56303936	-1.98652196	3.04507828	1	1.54043949	5.14647770	1.12147820
1	-0.90856063	-2.60202193	1.60147822	1	1.40863943	5.62537766	-0.59422189
6	-4.53376055	-0.53282201	-0.28012189	1	0.70553941	0.86647797	-3.86502171
6	-5.30926037	-1.01792192	0.81717813	1	1.60943949	0.06927797	-2.57132173
6	-6.69186068	-0.80672204	0.79387814	1	-0.15646061	-0.10522203	-2.67032194
1	-7.30406046	-1.14322197	1.62317812	1	0.55073941	-2.94812202	3.56927824
6	-7.32366037	-0.18152203	-0.27482188	1	1.58933949	-1.60492194	3.08867812

6	-6.56796074	0.20687798	-1.37492180	1	-0.08376060	-1.28902197	3.58617806
1	-7.08386040	0.65197796	-2.21872187				

DI-2i

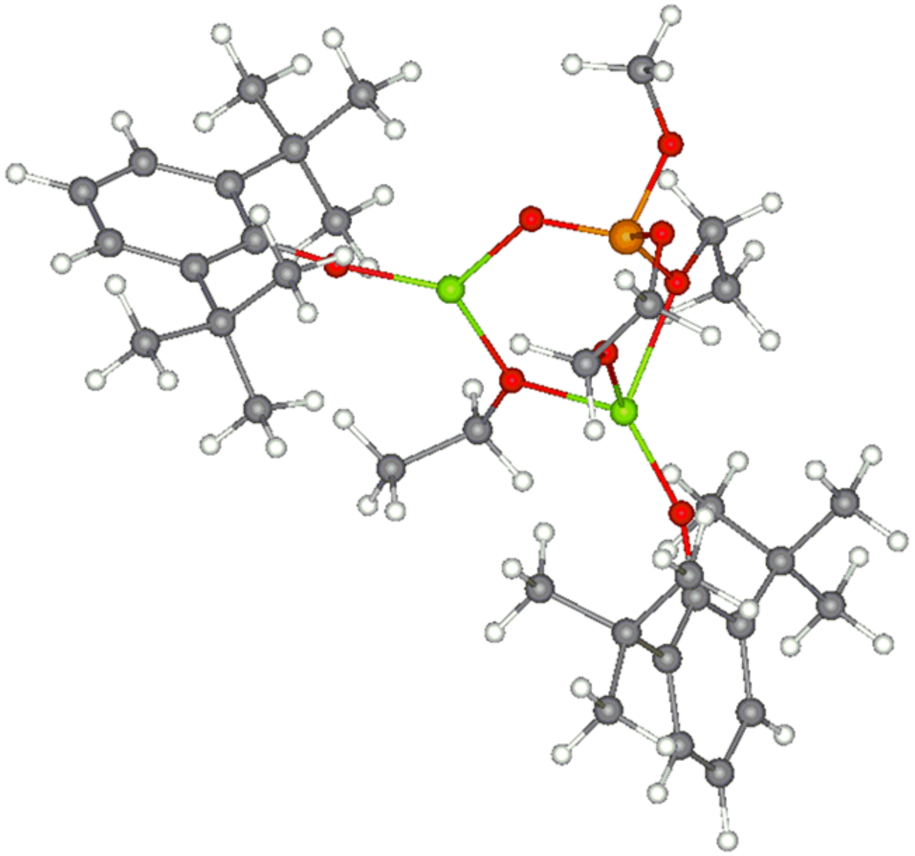

Zero-point vibrational energy	2368512.0 (Joules/Mol)
	566.08796 (Kcal/Mol)
Zero-point correction=	0.902119 (Hartree/Particle)
Thermal correction to Energy=	0.956579
Thermal correction to Enthalpy=	0.957524
Thermal correction to Gibbs Free Energy=	0.812642
Sum of electronic and zero-point Energies=	-2711.267556
Sum of electronic and thermal Energies=	-2711.213095
Sum of electronic and thermal Enthalpies=	-2711.212151
Sum of electronic and thermal Free Energies=	-2711.357032

cartesian

12	1.67499804	-0.51287043	-0.19634765	6	-5.29080200	0.27622956	1.35405231
8	-1.02200186	-2.62527037	-1.12104762	6	-3.66490197	2.52142954	-1.38864768
8	3.30439806	0.21922956	0.19255234	6	-5.24330187	-1.15347040	1.92045224
6	-1.04810190	-3.90587044	-0.40454766	6	-2.19680190	2.69772959	-0.95614767
1	-0.02390195	-4.18797064	-0.16254766	1	-1.76220191	1.78602958	-0.54074764
1	-1.46280193	-4.65077066	-1.08384764	1	-1.57360196	3.01812959	-1.80014765
6	4.52779818	0.60812956	0.54775232	1	-2.11990190	3.46272945	-0.17664766

6	5.08319807	1.79632950	-0.01854765	6	-3.79390192	1.54562950	-2.57594752
6	6.34649801	2.20782948	0.41665232	1	-3.46130180	0.53732955	-2.32134748
1	6.78929806	3.11282945	0.01445234	1	-4.84070206	1.47362959	-2.88934755
6	7.07179785	1.48872960	1.36055231	1	-3.21800184	1.90882945	-3.43694758
6	6.53889799	0.31142956	1.87345231	6	-4.11440182	3.89642954	-1.91704762
1	7.12899828	-0.24587044	2.59315252	1	-5.14900208	3.88272953	-2.27424765
6	5.28149796	-0.16217044	1.48595238	1	-4.01930189	4.68492937	-1.16374767
6	4.33689785	2.60452962	-1.09724760	1	-3.48030186	4.18212938	-2.76404762
6	4.75329828	-1.49427044	2.05225253	6	-6.39050198	-1.44227040	2.90485239
6	3.00459814	3.15532947	-0.55184764	1	-6.34800196	-0.80477047	3.79415250
1	2.34289813	2.35582948	-0.21564767	1	-7.37500191	-1.32317042	2.44075251
1	2.48449802	3.73502946	-1.32474768	1	-6.31540203	-2.48047042	3.24765253
1	3.18069816	3.81732965	0.30285233	6	-5.39370203	-2.16877055	0.76905233
6	4.08539820	1.72602952	-2.34054756	1	-4.64100170	-2.01727033	-0.00644766
1	3.52129817	0.82372957	-2.09654760	1	-5.31860209	-3.19627047	1.14715230
1	5.03499794	1.40892959	-2.78484750	1	-6.37700176	-2.06007028	0.29855233
1	3.53159809	2.29122949	-3.10124755	6	-3.93650198	-1.37837040	2.70335245
6	5.14709806	3.82112956	-1.57874763	1	-3.88340187	-0.69627047	3.55875254
1	6.10729790	3.53142953	-2.01784754	1	-3.88200188	-2.40467048	3.08715248
1	5.33869791	4.53752947	-0.77294767	1	-3.06010199	-1.19647050	2.08065248
1	4.58039808	4.34902954	-2.35414767	6	-1.88130188	-3.74227047	0.84255230
6	5.75319815	-2.16137052	3.01415253	1	0.17199805	-0.48157042	2.75805235
1	5.95649815	-1.54447043	3.89555240	1	-7.19010210	3.10692954	1.64845228
1	6.70569801	-2.39047050	2.52565241	1	8.04979801	1.83642960	1.68395233
1	5.33399820	-3.10917044	3.37075233	8	1.46489811	-1.74247050	-1.67824769
6	4.51669788	-2.50327039	0.90895236	15	0.15659803	-2.28977036	-2.27494764
1	3.85479808	-2.11167049	0.13385235	8	-0.10290196	-2.14037037	-3.88074756
1	4.08589792	-3.43417048	1.29835236	8	-0.68770200	-0.61697042	-2.14054751
1	5.46489811	-2.75177050	0.42065233	6	-0.55960196	-0.89927047	-4.42314768
6	3.46109819	-1.27497041	2.86395240	6	-0.42800194	0.12992956	-3.31174755
1	3.65179801	-0.62027043	3.72095251	1	-1.60090196	-1.03177047	-4.73054790
1	3.07699800	-2.22867036	3.24695253	1	0.05639805	-0.66567045	-5.29374790
1	2.67729807	-0.80017048	2.26985240	1	0.58509803	0.55002958	-3.27454758
12	-1.64040196	-0.66017044	-0.39464766	1	-1.15310192	0.93782949	-3.40954757
8	-0.04410195	-0.28587043	0.70425230	8	0.49389806	-3.88217044	-2.44884753
8	-3.35870194	-0.06597044	-0.02754766	6	1.63049805	-4.28267050	-3.22044754
6	-0.20570195	0.24852957	2.02985239	1	1.45199811	-4.10807037	-4.28474760
6	0.50109804	1.57962954	2.21535254	1	2.52589798	-3.74537039	-2.89584756
1	-1.27660191	0.37602955	2.23235250	1	1.76159811	-5.35147047	-3.04264760
6	-4.36170197	0.72992957	0.36435235	1	-1.92720187	-4.70407057	1.36355233
6	-4.53260183	2.02642965	-0.21664765	1	-1.43450189	-3.01947045	1.53195238

6	-5.54990196	2.85192966	0.27405232	1	-2.90540195	-3.44007039	0.61595231
1	-5.68680191	3.84472966	-0.14014766	1	0.34569806	1.94562960	3.23615241
6	-6.41260195	2.44132948	1.28205228	1	1.57989812	1.48932958	2.05375242
6	-6.28440189	1.15592957	1.79375231	1	0.10799804	2.33012962	1.52325237
1	-6.98860168	0.83712953	2.55455256				

DI-2r

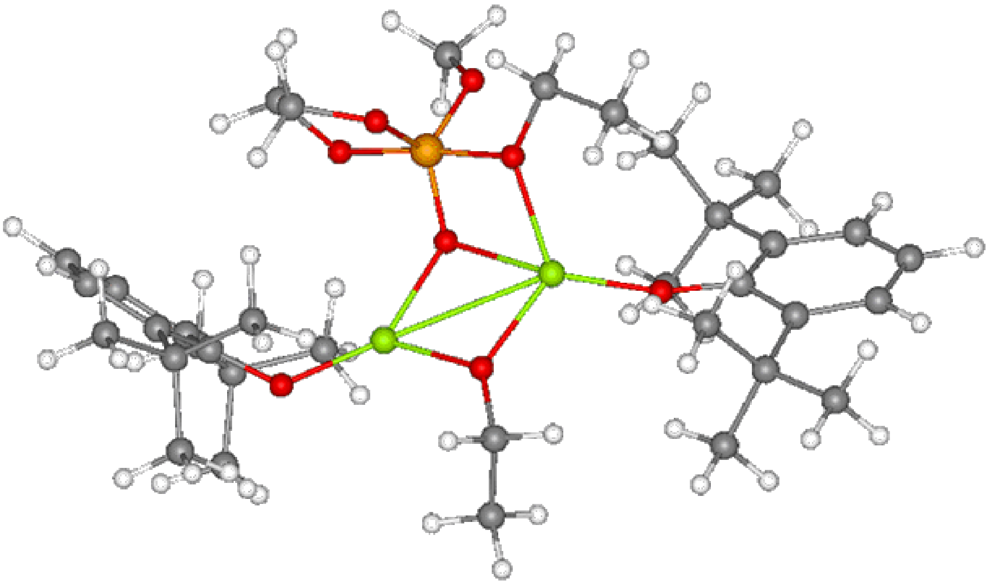

Zero-point vibrational energy	2369398.6 (Joules/Mol)
	566.29986 (Kcal/Mol)
Zero-point correction=	0.902456 (Hartree/Particle)
Thermal correction to Energy=	0.957493
Thermal correction to Enthalpy=	0.958438
Thermal correction to Gibbs Free Energy=	0.809607
Sum of electronic and zero-point Energies=	-2711.266067
Sum of electronic and thermal Energies=	-2711.211030
Sum of electronic and thermal Enthalpies=	-2711.210086
Sum of electronic and thermal Free Energies=	-2711.358916

cartesian

12	0.96483839	0.02281040	0.50933141	6	-5.57366133	0.80051023	0.66733158
8	0.46853828	-1.07198989	2.20163035	6	-4.45956135	-1.17289007	-2.48956966
8	2.73793840	0.08961041	-0.02976881	6	-5.29746151	2.16231060	1.33503032
6	1.09273827	-1.38839006	3.46463037	6	-4.70396137	-0.03008961	-3.49746966
1	1.46683836	-2.41448951	3.41163039	1	-4.33646154	0.92161006	-3.10526967
1	0.32983828	-1.32879007	4.24563026	1	-4.19446182	-0.23638959	-4.44726944
6	4.05763865	-0.07248962	-0.13176875	1	-5.77336121	0.07631044	-3.70426965
6	4.60623884	-1.32869005	-0.54256845	6	-2.93296146	-1.39389002	-2.33776975
6	5.99693871	-1.47749007	-0.55256844	1	-2.38236189	-0.45438975	-2.50446963
1	6.43523884	-2.42588949	-0.84456867	1	-2.67156172	-1.85989010	-1.37976968
6	6.85373878	-0.44098973	-0.20196867	1	-2.55366182	-2.06388974	-3.11716962
6	6.31403875	0.79921025	0.11933121	6	-5.02536154	-2.46488953	-3.10516953
1	6.99723864	1.60980999	0.34843129	1	-4.86506128	-3.33568954	-2.45976973

6	4.93463850	1.02570999	0.14073120
6	3.71443892	-2.50618958	-0.97806954
6	4.40243864	2.45321059	0.37213132
6	2.76843834	-2.08068967	-2.12036967
1	2.14693832	-1.22719014	-1.84486961
1	2.11313820	-2.91368937	-2.40386963
1	3.34653854	-1.79259002	-3.00496960
6	2.91643810	-3.04498959	0.22223118
1	2.29413843	-2.28088951	0.68933153
1	3.59663868	-3.42508936	0.99243158
1	2.26243830	-3.86908937	-0.08716880
6	4.53173876	-3.69228935	-1.52296960
1	5.18153858	-4.13778830	-0.76246846
1	5.14993858	-3.40828943	-2.38086963
1	3.84533882	-4.47778749	-1.85936952
6	5.53523874	3.47521043	0.58173144
1	6.21103859	3.52501059	-0.27786863
1	6.12973881	3.26041055	1.47633040
1	5.10033846	4.47271252	0.71113157
6	3.50833845	2.54291058	1.62253034
1	2.62643814	1.90690994	1.53363037
1	3.16933823	3.57421064	1.77973032
1	4.05693865	2.22811055	2.51663041
6	3.62683868	2.90131044	-0.88306880
1	4.29783869	2.93831062	-1.74796963
1	3.20583820	3.90501165	-0.74206841
1	2.81713820	2.20831060	-1.11766958
12	-1.88266170	0.32251024	-0.47046849
8	-0.28846177	1.39060998	-0.19816867
8	-3.62086153	0.95631003	-0.71096843
6	-0.14346179	2.78561044	-0.45736849
6	-0.30086178	3.10601044	-1.93486953
1	-0.89216173	3.34021044	0.12493120
6	-4.76066113	0.32021022	-0.40276855
6	-5.16246128	-0.81638974	-1.16366959
6	-6.27276134	-1.54579008	-0.72576845
1	-6.58666134	-2.43598938	-1.26086962
6	-7.00976133	-1.15389013	0.38833135
6	-6.67536116	0.02661041	1.04793119
1	-7.30326128	0.34841025	1.87253034
1	-6.09566116	-2.38388944	-3.31546974
1	-4.52586126	-2.66318941	-4.05956984
6	-6.38006115	2.52631044	2.36573029
1	-7.37766123	2.56911063	1.91673040
1	-6.41086149	1.82450998	3.20683026
1	-6.16626120	3.51781058	2.77953029
6	-3.94946146	2.18441057	2.08133054
1	-3.11016178	2.01491046	1.40403032
1	-3.79596186	3.16081047	2.55623031
1	-3.92116165	1.42860997	2.87383032
6	-5.30716133	3.26691055	0.25723121
1	-6.28296137	3.31061053	-0.23846860
1	-5.11886120	4.24571228	0.71523154
1	-4.54486132	3.08731055	-0.50276846
6	2.21833849	-0.41148975	3.72773027
1	0.84133828	3.11661053	-0.11106877
1	-7.86576128	-1.74009001	0.71353155
1	7.93073559	-0.58978975	-0.20676866
8	-0.75616169	-1.12629008	0.26863119
15	-0.95616174	-1.93169010	1.61073041
8	-2.40926170	-2.60908937	1.03453124
8	-1.72976172	-1.37459004	2.94473028
6	-3.33836174	-2.86498952	2.07263041
6	-3.11476183	-1.73309004	3.06793046
1	-3.15056181	-3.84518957	2.53353047
1	-4.34536171	-2.85158944	1.65163040
1	-3.71906185	-0.85718977	2.82143044
1	-3.29286146	-2.02478981	4.10483027
8	-0.14886180	-3.32128954	1.76693034
6	-0.54666173	-4.55328751	1.14763057
1	-1.44016171	-4.96658754	1.62003040
1	-0.72746170	-4.41608763	0.08073123
1	0.29223830	-5.23388767	1.29733038
1	2.70433855	-0.66188973	4.67633057
1	2.97643852	-0.45398974	2.94083047
1	1.84343803	0.61371022	3.80503035
1	-0.17406181	4.17961264	-2.11146975
1	0.44593829	2.57181048	-2.52996969
1	-1.29926169	2.82951045	-2.29446959

DTS-23i

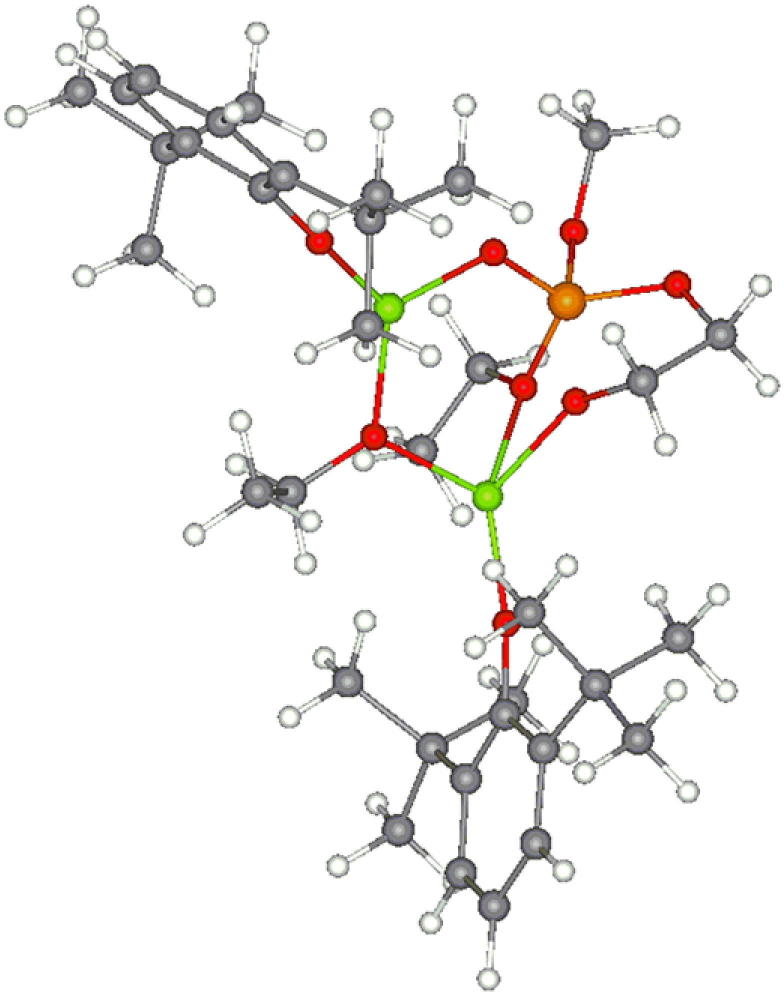

Zero-point vibrational energy	2368509.7 (Joules/Mol)
	566.08739 (Kcal/Mol)
Zero-point correction=	0.902118 (Hartree/Particle)
Thermal correction to Energy=	0.956579
Thermal correction to Enthalpy=	0.957523
Thermal correction to Gibbs Free Energy=	0.812635
Sum of electronic and zero-point Energies=	-2711.267557
Sum of electronic and thermal Energies=	-2711.213096
Sum of electronic and thermal Enthalpies=	-2711.212152
Sum of electronic and thermal Free Energies=	-2711.357039

cartesian

12	1.67498267	-0.51273811	-0.19668184	6	-5.29091740	0.27596188	1.35421813
8	-1.02201724	-2.62503815	-1.12118185	6	-3.66491723	2.52196193	-1.38788188
8	3.30448294	0.21916185	0.19251816	6	-5.24321699	-1.15383816	1.92021823
6	-1.04781735	-3.90573812	-0.40478185	6	-2.19681716	2.69846201	-0.95528185

1	-0.02361726	-4.18783808	-0.16288184	1	-1.76221728	1.78676188	-0.53968185
1	-1.46261728	-4.65063810	-1.08408189	1	-1.57361734	3.01896191	-1.79918194
6	4.52788305	0.60806185	0.54751819	1	-2.12011719	3.46346188	-0.17568184
6	5.08328295	1.79626191	-0.01898184	6	-3.79361725	1.54626191	-2.57528186
6	6.34658289	2.20776200	0.41611814	1	-3.46081710	0.53796184	-2.32078195
1	6.78928280	3.11266208	0.01381816	1	-4.84031725	1.47406185	-2.88888192
6	7.07198286	1.48856187	1.35991812	1	-3.21771717	1.90966189	-3.43618178
6	6.53918266	0.31136185	1.87291813	6	-4.11461735	3.89686203	-1.91618192
1	7.12928295	-0.24593812	2.59251809	1	-5.14911699	3.88306189	-2.27348185
6	5.28158283	-0.16223814	1.48571813	1	-4.01981735	4.68526220	-1.16268182
6	4.33678293	2.60456204	-1.09748185	1	-3.48041725	4.18286180	-2.76298189
6	4.75348282	-1.49433815	2.05211806	6	-6.39051723	-1.44303811	2.90441823
6	3.00468278	3.15536189	-0.55178183	1	-6.34831715	-0.80563813	3.79381800
1	2.34298277	2.35596204	-0.21548185	1	-7.37501717	-1.32403815	2.44031811
1	2.48448277	3.73516202	-1.32458186	1	-6.31531715	-2.48123813	3.24701810
1	3.18098283	3.81746197	0.30281818	6	-5.39341736	-2.16893792	0.76871818
6	4.08498287	1.72606182	-2.34068179	1	-4.64051723	-2.01713800	-0.00678185
1	3.52068281	0.82376188	-2.09658194	1	-5.31811714	-3.19653797	1.14651811
1	5.03438282	1.40876186	-2.78518200	1	-6.37661695	-2.06023812	0.29811817
1	3.53108287	2.29126191	-3.10128188	6	-3.93651724	-1.37873816	2.70331812
6	5.14698267	3.82096195	-1.57918191	1	-3.88351727	-0.69673812	3.55871820
1	6.10708284	3.53116202	-2.01858187	1	-3.88201714	-2.40503812	3.08711815
1	5.33888292	4.53736210	-0.77348185	1	-3.06001711	-1.19673812	2.08071804
1	4.58018303	4.34896183	-2.35438180	6	-1.88091731	-3.74223804	0.84241813
6	5.75328302	-2.16133809	3.01401806	1	0.17268273	-0.48263812	2.75791812
1	5.95658302	-1.54423809	3.89531827	1	-7.19051695	3.10636187	1.64891815
1	6.70578289	-2.39053798	2.52561808	1	8.04998207	1.83626187	1.68321812
1	5.33398294	-3.10893798	3.37081814	8	1.46488273	-1.74223816	-1.67878187
6	4.51678276	-2.50333810	0.90881819	15	0.15648274	-2.28943801	-2.27528191
1	3.85478282	-2.11193800	0.13371815	8	-0.10341725	-2.13993812	-3.88098192
1	4.08618259	-3.43433809	1.29831815	8	-0.68781722	-0.61663812	-2.14048171
1	5.46508265	-2.75173807	0.42041814	6	-0.56011724	-0.89873815	-4.42308187
6	3.46118283	-1.27493811	2.86371803	6	-0.42831728	0.13036187	-3.31158185
1	3.65188289	-0.62003815	3.72051811	1	-1.60151732	-1.03113806	-4.73028183
1	3.07718277	-2.22863793	3.24681807	1	0.05568274	-0.66503817	-5.29388142
1	2.67738271	-0.80033815	2.26941800	1	0.58478278	0.55046183	-3.27448177
12	-1.64041734	-0.66023815	-0.39448184	1	-1.15331733	0.93826187	-3.40928197
8	-0.04401726	-0.28603813	0.70421815	8	0.49368274	-3.88183808	-2.44938183
8	-3.35861707	-0.06573813	-0.02728184	6	1.63018274	-4.28223801	-3.22108197
6	-0.20531726	0.24776186	2.03001809	1	1.45168269	-4.10753822	-4.28528166
6	0.50128275	1.57896185	2.21601820	1	2.52568293	-3.74503803	-2.89658189

1	-1.27621734	0.37506187	2.23291802	1	1.76138270	-5.35113811	-3.04348183
6	-4.36171722	0.72996187	0.36461818	1	-1.92661726	-4.70403814	1.36341810
6	-4.53271723	2.02656198	-0.21608184	1	-1.43411732	-3.01943803	1.53181815
6	-5.55011702	2.85186195	0.27471817	1	-2.90511727	-3.44013810	0.61611819
1	-5.68711710	3.84466195	-0.13938184	1	0.34598273	1.94446194	3.23701811
6	-6.41291714	2.44086194	1.28241813	1	1.58008265	1.48876190	2.05421805
6	-6.28461695	1.15546191	1.79391813	1	0.10798274	2.32956195	1.52421808
1	-6.98891735	0.83636183	2.55451822				

DI-3i

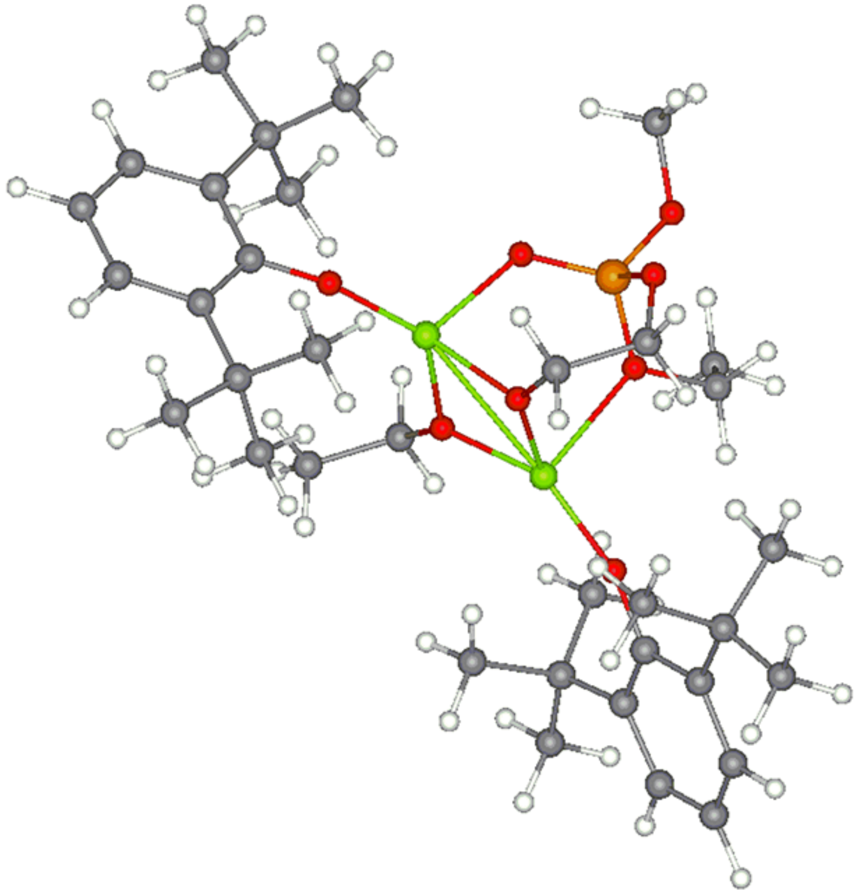

Zero-point vibrational energy	2366479.9 (Joules/Mol)
	565.60227 (Kcal/Mol)
Zero-point correction=	0.901345 (Hartree/Particle)
Thermal correction to Energy=	0.957150
Thermal correction to Enthalpy=	0.958094
Thermal correction to Gibbs Free Energy=	0.809885
Sum of electronic and zero-point Energies=	-2711.292350
Sum of electronic and thermal Energies=	-2711.236545
Sum of electronic and thermal Enthalpies=	-2711.235601
Sum of electronic and thermal Free Energies=	-2711.383810

cartesian

12	1.61461520	-0.36706188	-0.07907338	6	-4.84858465	1.19083810	1.37392652
8	-1.22338474	-2.45706201	0.05442663	6	-4.65568447	0.42493814	-2.44847322
8	3.35091519	0.22053812	-0.31317338	6	-4.00308466	1.15523803	2.66052675
6	-2.44038486	-3.27376199	0.28832662	6	-3.38218474	1.19933808	-2.84067321
1	-2.52978468	-3.99146199	-0.52937341	1	-2.57638478	1.05343807	-2.11997342
1	-3.25188470	-2.54916191	0.21852662	1	-3.02798486	0.88103813	-3.82947326

6	4.53621531	0.81893814	-0.22097339	1	-3.58948469	2.27333808	-2.89137340
6	4.73921537	2.10923815	-0.80527335	6	-4.39958429	-1.09366190	-2.45347333
6	5.99331570	2.71213818	-0.66837335	1	-3.66208482	-1.36666191	-1.69697344
1	6.17031527	3.69323802	-1.09637344	1	-5.32428455	-1.63826191	-2.23337340
6	7.04101563	2.09273815	0.00402662	1	-4.04288435	-1.42506194	-3.43747330
6	6.84261560	0.82823813	0.54702663	6	-5.68598461	0.69663817	-3.55937338
1	7.67491531	0.35493815	1.05692661	1	-6.61968470	0.14593811	-3.40527320
6	5.61631536	0.16363813	0.45032662	1	-5.92418432	1.76113808	-3.65247321
6	3.62351537	2.83103800	-1.58467340	1	-5.27238464	0.37073815	-4.52047348
6	5.46011543	-1.24646187	1.05032659	6	-4.77428436	1.66663814	3.89142680
6	2.43041515	3.14893818	-0.66197336	1	-5.07978439	2.71243811	3.78642678
1	2.02311540	2.25413799	-0.18937337	1	-5.66488457	1.06553805	4.10242653
1	1.62401521	3.63333797	-1.22637343	1	-4.12478447	1.60713804	4.77172661
1	2.73791528	3.82903814	0.13942662	6	-3.56968474	-0.28406188	2.99982667
6	3.17371535	1.97143817	-2.78357339	1	-2.99098468	-0.73626184	2.19252658
1	2.84851527	0.98033816	-2.46437335	1	-2.96518469	-0.30206186	3.91452670
1	4.00191545	1.83953810	-3.48837328	1	-4.44928455	-0.91566187	3.16642666
1	2.34801531	2.45803809	-3.31777334	6	-2.78458476	2.08633804	2.50242662
6	4.08871555	4.17793846	-2.16637325	1	-3.11688471	3.11493802	2.32822680
1	4.92661572	4.06133842	-2.86157322	1	-2.16608477	2.08133817	3.40732670
1	4.38411570	4.88833809	-1.38707340	1	-2.14978480	1.80963802	1.65742660
1	3.26241517	4.63323832	-2.72417331	6	-2.39118481	-3.94196200	1.64172661
6	6.76481533	-1.76256192	1.68442655	1	0.99191523	-0.64816189	3.04002666
1	7.09921551	-1.13706183	2.51892662	1	-7.94618464	2.23523808	0.36282665
1	7.58001566	-1.83076191	0.95642668	1	8.00471497	2.58753800	0.09712662
1	6.59731531	-2.77036190	2.08142662	8	1.35541523	-2.41036201	-0.14297338
6	5.07991552	-2.25656199	-0.05147338	15	0.10311527	-3.04556203	-0.65357339
1	4.15501547	-1.97696197	-0.55627340	8	-0.12138474	-3.01386189	-2.22037339
1	4.95401525	-3.25906181	0.37772661	8	0.04091527	-0.25606188	-1.34727347
1	5.87381554	-2.31026196	-0.80457336	6	-0.63688475	-1.88536191	-2.98697329
6	4.40731525	-1.24476194	2.17622662	6	0.10091527	-0.59226185	-2.70717335
1	4.71601534	-0.57806188	2.98852658	1	-1.69948471	-1.79016197	-2.75307322
1	4.28221560	-2.25206184	2.59232664	1	-0.52628475	-2.19066191	-4.02937365
1	3.43391514	-0.90166187	1.82212663	1	1.14491522	-0.69216186	-3.03557324
12	-1.29728472	-0.13366188	0.09642662	1	-0.35898474	0.18423812	-3.33497334
8	0.23681527	0.08953811	1.26382661	8	0.03351526	-4.59446192	-0.32687336
8	-3.12338471	0.19233812	0.02692662	6	1.20321524	-5.42626190	-0.52757341
6	0.42971525	0.22023813	2.66512680	1	1.37811530	-5.56396151	-1.59627342
6	1.16941524	1.50003803	3.02182674	1	2.07191515	-4.97166157	-0.04937338
1	-0.54288477	0.20473813	3.17262673	1	0.96911526	-6.38176155	-0.06127338
6	-4.34198427	0.73953813	0.11442661	1	-3.34008479	-4.46286154	1.80562663

6	-5.15178442	0.86613816	-1.05877340	1	-1.58208477	-4.67186165	1.69822657
6	-6.43538427	1.40573812	-0.93127334	1	-2.26858473	-3.20476198	2.43802667
1	-7.06718445	1.50923812	-1.80657339	1	1.30771530	1.57293808	4.10652685
6	-6.94258451	1.82343805	0.29272664	1	2.15801525	1.52693808	2.55302668
6	-6.14418459	1.71533811	1.42412663	1	0.60931528	2.37763810	2.68562675
1	-6.55098438	2.05683804	2.36932659				

DI-3

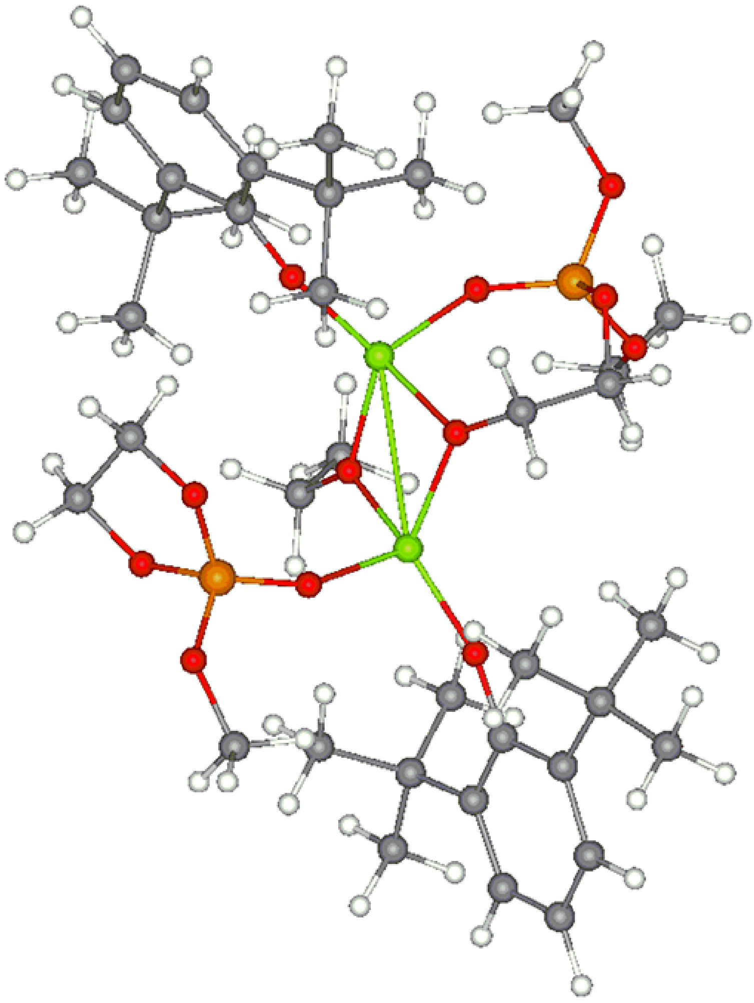

Zero-point vibrational energy	2674867.4 (Joules/Mol)
	639.30865 (Kcal/Mol)
Zero-point correction=	1.018803 (Hartree/Particle)
Thermal correction to Energy=	1.084629
Thermal correction to Enthalpy=	1.085573
Thermal correction to Gibbs Free Energy=	0.911889
Sum of electronic and zero-point Energies=	-3471.884180
Sum of electronic and thermal Energies=	-3471.818355
Sum of electronic and thermal Enthalpies=	-3471.817411
Sum of electronic and thermal Free Energies=	-3471.991094

cartesian

12	1.39179766	0.57710826	-0.25066918	6	-3.80180240	2.07320833	2.36733079
8	0.09679771	4.50490808	-0.26006916	1	-3.11060238	1.97310817	1.52943087
8	2.98979759	-0.39779174	0.02983083	1	-4.50360250	2.87960839	2.12753081
6	-0.16790231	4.64860821	-1.68766928	1	-3.24320245	2.37220836	3.26393080

1	-1.24830234	4.52560806	-1.77776909	6	-5.52230215	1.03390825	3.79713082
1	0.31869772	3.82960844	-2.22216916	1	-6.23220253	1.83750820	3.57523084
6	4.22669744	-0.85729170	0.19313082	1	-6.08930254	0.14430827	4.09133101
6	5.01029778	-1.25959182	-0.93976921	1	-4.93690252	1.34740818	4.66933107
6	6.28569746	-1.79129171	-0.72456914	6	-5.69490242	-0.71369171	-3.56096911
1	6.88849783	-2.10319161	-1.57056928	1	-6.30160236	-1.61109173	-3.40016913
6	6.82209778	-1.93549180	0.54933083	1	-6.36930227	0.13780826	-3.70056915
6	6.07359743	-1.51639175	1.64303088	1	-5.14950228	-0.85649168	-4.50086927
1	6.51259756	-1.61879182	2.62953091	6	-3.85150242	0.75510830	-2.84016919
6	4.79399776	-0.96899170	1.50673079	1	-3.09920239	1.00830829	-2.09236908
6	4.48429775	-1.12319171	-2.37976909	1	-3.35030246	0.57290828	-3.79896903
6	4.05269766	-0.46679175	2.75973082	1	-4.50490236	1.62610829	-2.96406913
6	4.17169762	0.35140827	-2.70316911	6	-3.81280231	-1.74159181	-2.34456921
1	3.47419763	0.79160827	-1.98986912	1	-4.44170237	-2.61649156	-2.14306903
1	3.74539757	0.44440827	-3.70946908	1	-3.28870225	-1.91889179	-3.29176903
1	5.09019756	0.94770831	-2.67156911	1	-3.07240224	-1.68169177	-1.54456925
6	3.23679757	-2.00619173	-2.56746912	6	0.28459772	6.00600815	-2.18086910
1	2.45659757	-1.75489175	-1.84716916	1	-1.17360234	-0.79989171	-2.98006916
1	3.50799775	-3.06029153	-2.43216920	1	-8.51640224	-0.21449172	0.27673084
1	2.82869768	-1.89569175	-3.57966900	1	7.81499767	-2.35639167	0.68723083
6	5.50659752	-1.58499181	-3.43406916	8	1.77759767	2.54530835	-0.50296915
1	5.76489782	-2.64409161	-3.32956910	15	1.36529768	3.74910831	0.29643083
1	6.43149757	-0.99989170	-3.39926910	8	1.03799760	3.49020839	1.81553078
1	5.07549763	-1.45349181	-4.43306923	8	-0.06530229	0.74820828	1.10433078
6	4.86249781	-0.67829168	4.05183077	6	-0.18420230	2.85880828	2.31163096
1	5.81919765	-0.14559174	4.03803062	6	-0.07840229	1.34680820	2.37053084
1	5.06039762	-1.73739171	4.24933100	1	-1.02030230	3.17810845	1.68613076
1	4.28839779	-0.29189172	4.90163088	1	-0.30850229	3.27430844	3.31413078
6	2.72099757	-1.21459174	2.95723081	1	0.81979769	1.07940829	2.94313097
1	2.04709768	-1.08709180	2.10993099	1	-0.94200230	1.00620818	2.96023083
1	2.20999765	-0.85849172	3.86043096	8	2.50839758	4.84750795	0.32813084
1	2.90439773	-2.28669167	3.08873081	6	3.88119769	4.47290802	0.58343083
6	3.81819773	1.05120826	2.63783097	1	4.00609779	4.22620821	1.63923073
1	4.77939749	1.57590818	2.59463096	1	4.16879749	3.62680840	-0.04316917
1	3.26509762	1.43370819	3.50443077	1	4.47899771	5.34790802	0.33203083
1	3.26229763	1.30080819	1.73283076	1	0.01069771	6.11540794	-3.23506904
12	-1.44970238	-0.22009173	0.04933083	1	-0.19860230	6.80610800	-1.61476922
8	-0.08650229	-0.08029173	-1.38176918	1	1.36749768	6.12190819	-2.09306908
8	-3.28820229	0.18330827	0.05823083	1	0.01799771	0.52510828	-4.70756912
6	-0.17640230	-0.40039173	-2.75766921	1	-0.65910226	1.58970821	-3.46086907
6	0.08149771	0.80620831	-3.64976907	1	1.07899761	1.22220826	-3.47126913

1	0.53769773	-1.19659173	-3.00496912	8	-1.38730240	-2.17519164	0.62283081
6	-4.61310244	0.09440827	0.10953083	15	-0.78270227	-3.53289175	0.66303080
6	-5.31000233	0.33920828	1.33903074	8	0.53459769	-3.75829172	1.53243089
6	-6.70210218	0.21220827	1.36223078	8	-0.22020230	-4.01799154	-0.76026917
1	-7.24370241	0.38610825	2.28583097	6	1.66689765	-4.03399181	0.66473085
6	-7.43370247	-0.12579173	0.22963083	6	1.05839765	-4.67309189	-0.58336920
6	-6.75860214	-0.33209175	-0.96766919	1	2.33359766	-4.70629168	1.20373082
1	-7.34390211	-0.57849169	-1.84726906	1	2.17669773	-3.09289169	0.44833082
6	-5.36780214	-0.22849172	-1.06626916	1	1.64899766	-4.49019194	-1.47976923
6	-4.56860256	0.76030827	2.62043095	1	0.88449770	-5.74549198	-0.45646918
6	-4.68550253	-0.47339174	-2.42376900	8	-1.78460228	-4.60879183	1.24303079
6	-3.61410236	-0.35609174	3.08703089	6	-3.22160244	-4.44269180	1.13483071
1	-2.89510226	-0.63219172	2.31483078	1	-3.49940228	-3.39979172	1.28753090
1	-3.06150246	-0.04729173	3.98363090	1	-3.55110240	-4.78529167	0.15243083
1	-4.18310213	-1.25669181	3.34473085	1	-3.64950228	-5.07029200	1.91473079

DTS-34a

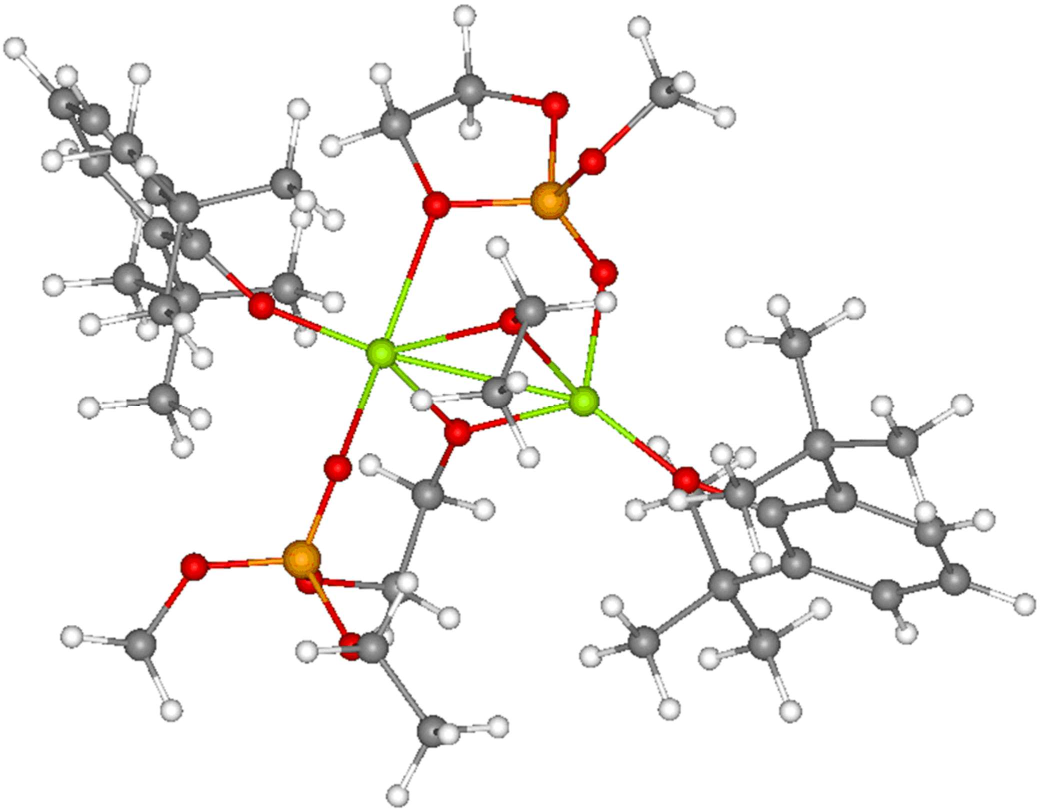

Zero-point vibrational energy	2677556.9 (Joules/Mol)
	639.95145 (Kcal/Mol)
Zero-point correction=	1.019828 (Hartree/Particle)
Thermal correction to Energy=	1.084439
Thermal correction to Enthalpy=	1.085384
Thermal correction to Gibbs Free Energy=	0.918929
Sum of electronic and zero-point Energies=	-3471.860748
Sum of electronic and thermal Energies=	-3471.796136
Sum of electronic and thermal Enthalpies=	-3471.795192
Sum of electronic and thermal Free Energies=	-3471.961647

cartesian

12	-1.28265727	-0.03242502	0.23479581	6	3.82534266	2.26117492	-2.16570401
8	-0.02175735	4.04617500	1.40289581	1	3.20124269	1.97767508	-1.31700420
8	-3.14255738	0.00317498	-0.16950417	1	4.54464293	3.00957489	-1.81520414
6	0.25744265	3.97557497	2.83069587	1	3.19954252	2.73347497	-2.93430400
1	0.35414264	2.92527485	3.11419582	6	5.43534279	1.57067502	-3.90140414
1	-0.59315735	4.40747499	3.36759591	1	6.16084290	2.31877494	-3.56560397
6	-4.37325716	-0.43862504	-0.38270417	1	5.97764301	0.77097499	-4.41650391
6	-5.17485714	-0.93472499	0.70109582	1	4.78894281	2.05317497	-4.64360428

6	-6.41485739	-1.51042497	0.40719581	6	6.13624287	-1.73722494	2.86529589
1	-7.02525711	-1.91222501	1.20909584	1	6.71434259	-2.57652521	2.46489596
6	-6.91295719	-1.58002496	-0.88950419	1	6.83264303	-0.93702501	3.13699603
6	-6.17815733	-1.00742507	-1.92190421	1	5.66094303	-2.08392501	3.79039598
1	-6.60475731	-1.02112508	-2.91950417	6	4.26764297	-0.14702502	2.62379599
6	-4.92965698	-0.41422501	-1.70750415	1	3.48854256	0.27857497	1.99059570
6	-4.72695732	-0.80962497	2.17099595	1	3.80824256	-0.53992504	3.53949594
6	-4.21045732	0.28447497	-2.87860417	1	4.94514275	0.66517502	2.90999603
6	-4.41285706	0.66347498	2.50219584	6	4.15094280	-2.47402501	1.60409582
1	-3.63775730	1.07557499	1.85689580	1	4.74034262	-3.27772522	1.14919579
1	-4.08395720	0.75667500	3.54519582	1	3.71604252	-2.85972500	2.53489590
1	-5.31295729	1.27607501	2.37969589	1	3.33864260	-2.24032521	0.91359580
6	-3.50545740	-1.70152497	2.45989585	6	1.53014266	4.74627495	3.09619594
1	-2.67515731	-1.49682498	1.78299582	1	1.40164268	-1.92992496	2.60949588
1	-3.76435733	-2.75992513	2.34099603	1	8.67454243	-0.35982502	-0.95410419
1	-3.15325737	-1.55722499	3.48869586	1	-7.87755728	-2.04102516	-1.08710420
6	-5.82245731	-1.25292504	3.15809584	8	-1.21375728	1.76137495	1.27319586
1	-6.07405710	-2.31462502	3.06069589	15	-1.18075728	3.16127491	0.75749582
1	-6.74115705	-0.66902500	3.04079604	8	-0.96235734	3.36557484	-0.79110420
1	-5.46525717	-1.09942496	4.18279600	8	0.17734265	0.56427503	-1.01190412
6	-5.06595707	0.31657496	-4.15790415	6	0.24614263	2.95247483	-1.49930418
1	-6.02145720	0.82797498	-4.00230408	6	0.12904266	1.53447497	-2.02480412
1	-5.27145720	-0.68502498	-4.55080414	1	1.10764265	3.06897497	-0.83770418
1	-4.52455711	0.86347502	-4.93820429	1	0.33394265	3.66207480	-2.32500410
6	-2.90335751	-0.44112504	-3.24980402	1	-0.80455738	1.45667505	-2.59670401
1	-2.21285748	-0.49842504	-2.40650415	1	0.95264262	1.39997506	-2.74050403
1	-2.39315748	0.07687498	-4.07170391	8	-2.56415749	3.87527490	1.04269576
1	-3.11575747	-1.46062505	-3.59380412	6	-2.84245753	5.20097494	0.54939580
6	-3.92255735	1.75297499	-2.50730395	1	-2.06245732	5.90057468	0.86069584
1	-4.86215734	2.28777480	-2.32920408	1	-2.92155743	5.18217468	-0.53860420
1	-3.39765739	2.26047492	-3.32700396	1	-3.79645753	5.48757505	0.98969579
1	-3.31695747	1.82767498	-1.60420418	1	1.75574267	4.71587467	4.16619587
12	1.67284274	-0.55152500	-0.35960418	1	2.37184262	4.30787468	2.55469584
8	0.27704263	-1.18282497	1.04519582	1	1.42634273	5.79207468	2.79569602
8	3.47754264	-0.07852502	-0.27670416	1	0.67944264	-0.32902503	4.30319595
6	0.46154267	-1.39182496	2.45529604	1	1.26074266	0.57497501	2.89729595
6	0.47504264	-0.09882502	3.25139594	1	-0.48175737	0.42547497	3.19549584
1	-0.34115735	-2.04182506	2.82149601	8	1.21214271	-2.38492513	-1.02110410
6	4.79894304	-0.14412501	-0.44540417	15	0.09014264	-3.02082515	-0.23900416
6	5.40224266	0.37087497	-1.63780415	8	-0.37725735	-4.33052492	-1.12060416
6	6.78884268	0.27297497	-1.78650415	8	-1.39045727	-2.32422519	-0.27530417

1	7.26254272	0.65117502	-2.68610406	6	-1.59535730	-4.07292509	-1.82540417
6	7.59864283	-0.29602501	-0.81090420	6	-2.39305735	-3.18122506	-0.89090419
6	7.01154280	-0.77252501	0.35499582	1	-2.08915734	-5.02712488	-2.01590419
1	7.65734291	-1.20282495	1.11279583	1	-1.37465727	-3.57622504	-2.77620411
6	5.63184261	-0.71072501	0.57229584	1	-3.11925745	-2.53622508	-1.38140416
6	4.56864262	1.04137504	-2.74450397	1	-2.87645745	-3.74742508	-0.09180418
6	5.04834270	-1.25412500	1.88859582	8	0.43254265	-3.75332522	1.13329577
6	3.58544254	0.03427498	-3.37210417	6	1.00444269	-5.08052492	1.14839578
1	2.91464257	-0.41012502	-2.63520408	1	0.29754263	-5.80312490	0.73939580
1	2.97594261	0.51677501	-4.14650393	1	1.93714273	-5.10142517	0.58139580
1	4.13154268	-0.79062498	-3.84130406	1	1.20604265	-5.29342508	2.19769597

DTS-34b

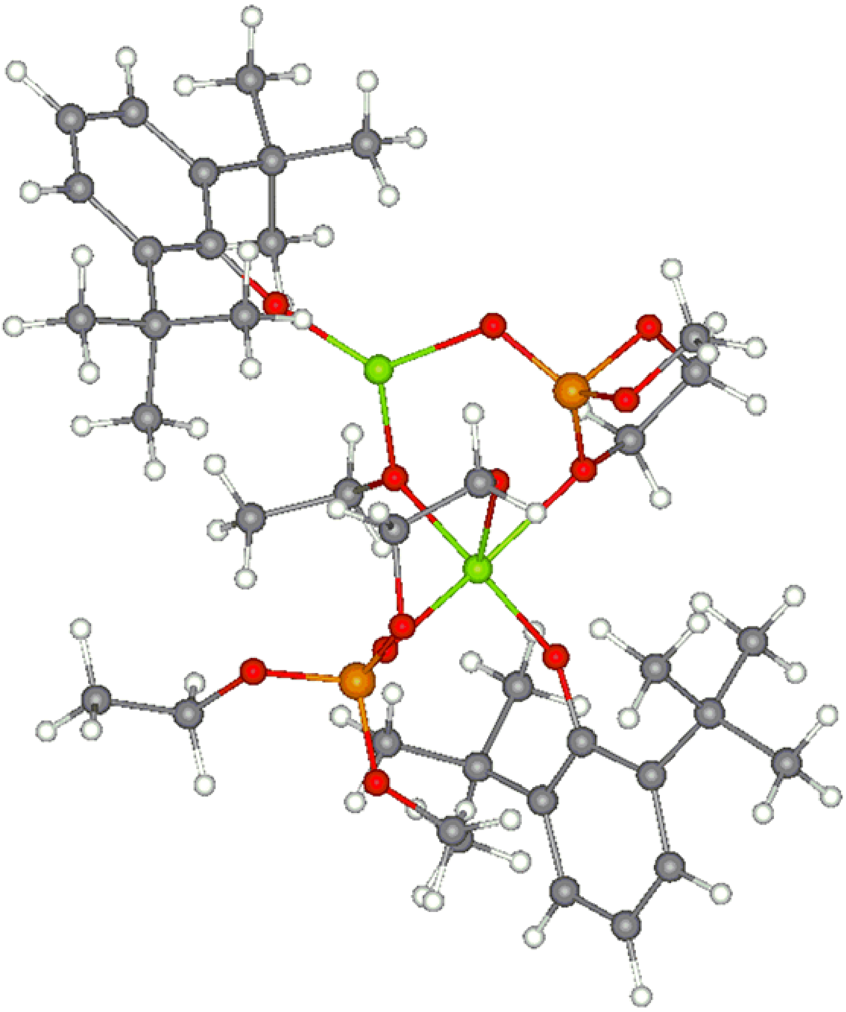

Zero-point vibrational energy	2675402.3 (Joules/Mol)
	639.43649 (Kcal/Mol)
Zero-point correction=	1.019007 (Hartree/Particle)
Thermal correction to Energy=	1.083277
Thermal correction to Enthalpy=	1.084221
Thermal correction to Gibbs Free Energy=	0.918416
Sum of electronic and zero-point Energies=	-3471.853723
Sum of electronic and thermal Energies=	-3471.789452
Sum of electronic and thermal Enthalpies=	-3471.788508
Sum of electronic and thermal Free Energies=	-3471.954313

cartesian

12	1.29129159	-0.34226170	-0.08221585	6	-3.59640837	2.72733831	0.77288413
8	0.35969162	3.82743835	1.27178407	1	-2.96420836	2.02323842	0.22948414
8	3.10579157	-0.68396175	-0.59791589	1	-4.02400827	3.42003846	0.03948414

6	0.39459160	4.69233799	0.09858415	1	-2.96940827	3.30873847	1.46068406
1	0.17149162	4.08373833	-0.78131580	6	-5.53350830	3.10563850	2.25438428
1	1.40479159	5.10153818	0.00438415	1	-5.97570801	3.81373835	1.54568410
6	4.36579180	-0.27166170	-0.68541586	1	-6.33610821	2.69263840	2.87408423
6	4.83299208	0.42013830	-1.85401595	1	-4.87240791	3.67693830	2.91618419
6	6.12199163	0.96313822	-1.84371579	6	-6.39640808	-2.62756157	-2.61391568
1	6.47889185	1.51523829	-2.70651579	1	-7.19890833	-3.03406167	-1.98971593
6	6.98129177	0.81353831	-0.76111591	1	-6.84230804	-1.96096170	-3.35941577
6	6.56899166	0.03703830	0.31688416	1	-5.94890833	-3.46906161	-3.15491581
1	7.27499199	-0.12886170	1.12368417	6	-4.23070812	-1.47176170	-2.78601575
6	5.29669189	-0.54076171	0.37808415	1	-3.43290830	-0.90526175	-2.30241585
6	3.96579170	0.57053828	-3.11951566	1	-3.78890848	-2.33436155	-3.30031586
6	4.96389198	-1.49926174	1.54058409	1	-4.66850805	-0.81556165	-3.54601574
6	2.82599163	1.57563829	-2.87391567	6	-4.75210810	-2.99276161	-0.81601584
1	2.20719171	1.30053830	-2.01991582	1	-5.55690813	-3.38786173	-0.18721586
1	2.18139172	1.65833831	-3.75801587	1	-4.32120800	-3.83286166	-1.37441587
1	3.23679161	2.57033849	-2.66561580	1	-3.98870826	-2.60636163	-0.13881585
6	3.40609169	-0.79856169	-3.55701566	6	-0.62590837	5.78913832	0.29508415
1	2.82039165	-1.26726174	-2.76551580	1	-1.17560840	-0.82816172	-3.00491571
1	4.22649193	-1.47796178	-3.81421590	1	-8.82320881	0.88993835	-0.44741586
1	2.77489161	-0.68476176	-4.44721603	1	7.97469187	1.25523829	-0.77711582
6	4.76819181	1.10573828	-4.32051611	8	1.39069164	1.66773832	0.28188413
1	5.63019180	0.47213832	-4.55461597	15	1.40019166	2.64243841	1.41748416
1	5.12499189	2.12973833	-4.16781616	8	1.01759160	1.97533834	2.80178428
1	4.12099171	1.12233829	-5.20471621	8	0.24859163	-0.85226166	1.67818415
6	6.17499208	-1.75896168	2.45518422	6	-0.23010837	1.23373830	2.90758419
1	6.51069164	-0.85566175	2.97628427	6	-0.02470837	-0.26436171	2.94988418
1	7.02549171	-2.17586160	1.90618408	1	-0.91790837	1.53343832	2.11188412
1	5.89299202	-2.48726153	3.22388434	1	-0.66230839	1.54823828	3.86008430
6	4.54909182	-2.86496162	0.96028411	1	0.78219163	-0.50006169	3.65028429
1	3.68309164	-2.75106168	0.30988413	1	-0.95230836	-0.68956172	3.35188413
1	4.29889202	-3.56626153	1.76668417	8	2.77019167	3.38123846	1.65738416
1	5.36869192	-3.29686165	0.37588415	6	4.06389189	2.78033829	1.38138413
6	3.84549165	-0.95596170	2.44778419	1	4.74879169	3.61393833	1.23618412
1	4.15289164	-0.02266170	2.93118429	1	4.37099171	2.18593836	2.24098420
1	3.61259151	-1.67666173	3.24108434	1	4.02369165	2.16273832	0.48428416
1	2.92459154	-0.77206171	1.89528418	1	-0.61380839	6.45223808	-0.57511580
12	-2.00660849	-0.87316167	-0.01961586	1	-1.63090837	5.37453794	0.39798415
8	-0.45520839	-0.41006172	-1.10321593	1	-0.39470840	6.38123798	1.18398416
8	-3.70660830	-0.16716170	-0.07151586	1	-1.16420841	1.52553833	-3.79821587
6	-0.54910839	-0.08486170	-2.49121571	1	-2.12610841	1.39563823	-2.31511569

6	-1.11490834	1.30673826	-2.72551584	1	-0.48070836	2.05993843	-2.24901581
1	0.44589162	-0.15866169	-2.94741583	8	-1.60670841	-2.38366151	1.13678420
6	-5.01350832	0.07973829	-0.15601584	15	-0.14330837	-2.69836164	1.45758414
6	-5.57710838	1.15743828	0.59518415	8	1.02079165	-2.47496152	0.28608415
6	-6.94360828	1.42083824	0.46498415	8	-0.17690837	-4.35046196	1.12438416
1	-7.39200830	2.23703837	1.02128410	6	1.16889167	-3.59306169	-0.60841584
6	-7.76350832	0.66193831	-0.36221585	6	0.86419159	-4.79116201	0.27148414
6	-7.21220827	-0.39896169	-1.07131588	1	2.18519163	-3.56656170	-0.99971592
1	-7.86960793	-0.98806167	-1.70161593	1	0.44569162	-3.48376155	-1.42191589
6	-5.85370827	-0.72156167	-0.99101591	1	0.50889164	-5.64926195	-0.30391586
6	-4.71830797	2.00823832	1.54748416	1	1.74819160	-5.08566189	0.85248411
6	-5.30770826	-1.92606175	-1.78121591	8	0.29939163	-2.85316157	2.99998426
6	-4.12740803	1.11713827	2.65928435	6	0.10539162	-4.06696177	3.74948430
1	-3.52330828	0.30523831	2.24858427	1	-0.91800839	-4.43176174	3.65138435
1	-3.50820827	1.71363831	3.34178424	1	0.80389160	-4.83866167	3.42378426
1	-4.92900801	0.66013825	3.24918413	1	0.30739161	-3.79606152	4.78688383

DI-4a

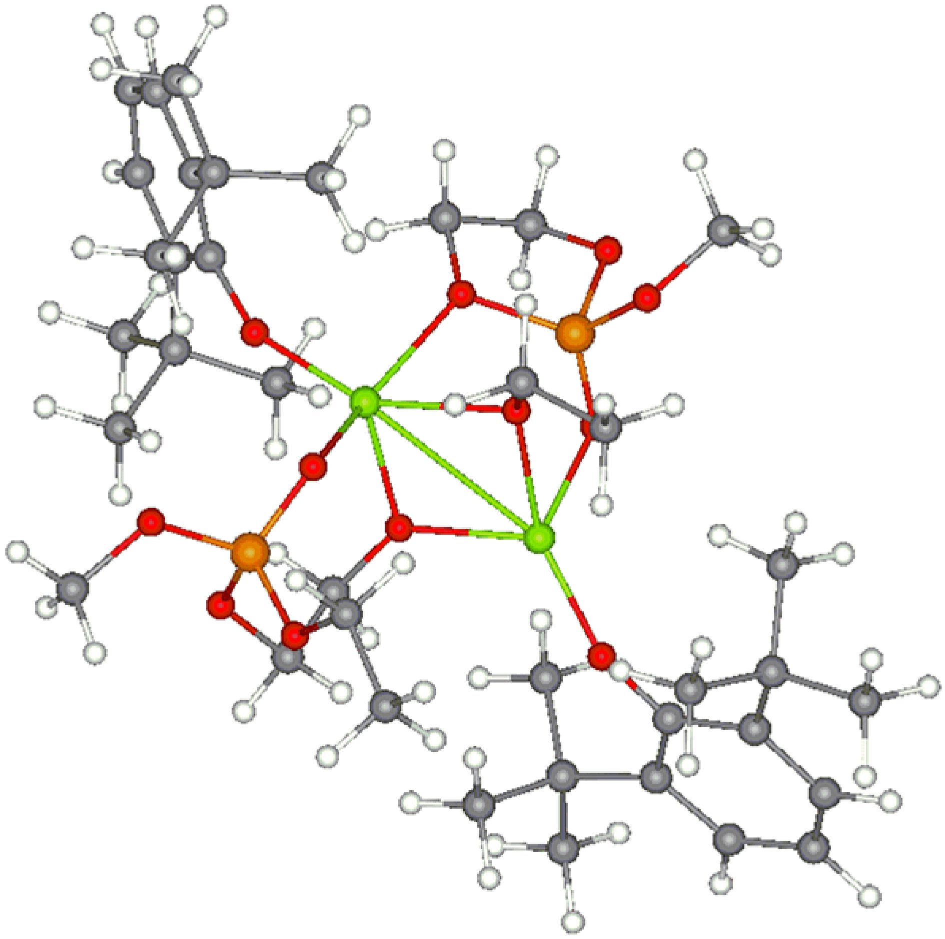

Zero-point vibrational energy	2675654.7 (Joules/Mol)
	639.49683 (Kcal/Mol)
Zero-point correction=	1.019103 (Hartree/Particle)
Thermal correction to Energy=	1.083295
Thermal correction to Enthalpy=	1.084239
Thermal correction to Gibbs Free Energy=	0.918799
Sum of electronic and zero-point Energies=	-3471.863012
Sum of electronic and thermal Energies=	-3471.798820
Sum of electronic and thermal Enthalpies=	-3471.797876
Sum of electronic and thermal Free Energies=	-3471.963316

cartesian

12	-1.40637171	0.01214166	0.29771671	6	3.64512825	1.60164165	-2.90508342
8	0.17102832	3.98694158	1.16441679	1	3.15262818	1.65724158	-1.93298328
8	-3.19937181	0.07704166	-0.34208328	1	4.46172810	2.33194160	-2.90658331
6	0.48162836	3.96464181	2.58651662	1	2.93572831	1.89354169	-3.69048333
1	0.56272835	2.92264175	2.90531659	6	4.90692806	0.24804164	-4.53638315

1	-0.34517166	4.43374205	3.12901664	1	5.71992826	0.98104167	-4.55278349
6	-4.38757181	-0.45495832	-0.60608327	1	5.31072807	-0.72355837	-4.83988333
6	-4.73387194	-0.81805837	-1.95178330	1	4.18112850	0.54984164	-5.30028343
6	-5.92967176	-1.50825834	-2.17508340	6	6.54142809	-0.69805837	2.77701664
1	-6.19477177	-1.81555831	-3.18128347	1	7.04202843	-1.66025841	2.62711668
6	-6.81177187	-1.81115842	-1.14388323	1	7.27972841	0.09954165	2.64241672
6	-6.52557182	-1.35525835	0.13841671	1	6.21242809	-0.66615838	3.82211661
1	-7.25327158	-1.54555833	0.92011672	6	4.67272806	0.82054162	2.26311660
6	-5.35117149	-0.65765834	0.43911672	1	3.78762817	1.03994167	1.66351676
6	-3.85027170	-0.42385834	-3.15178347	1	4.39512825	0.80834162	3.32461667
6	-5.15437174	-0.08535834	1.85771680	1	5.38542843	1.63964164	2.11451674
6	-2.49917173	-1.16335833	-3.12538338	6	4.38512850	-1.71005833	2.15211654
1	-1.93497169	-0.96465838	-2.21288347	1	4.88622808	-2.65105844	1.90181673
1	-1.87537169	-0.86245835	-3.97628355	1	4.11742830	-1.74135840	3.21491671
1	-2.65347171	-2.24605846	-3.20398331	1	3.46342826	-1.67405832	1.56901670
6	-3.63677168	1.10344160	-3.14908338	6	1.77722824	4.71504211	2.79291654
1	-3.19427180	1.44554162	-2.21268344	1	-0.72287166	-1.18415833	3.35421658
1	-4.59617186	1.61764169	-3.27428341	1	8.46752834	-0.94995838	-1.58868325
1	-2.98717165	1.40224159	-3.98208332	1	-7.72917175	-2.35945845	-1.34378326
6	-4.50317192	-0.77115834	-4.50188351	8	-1.29177177	1.86094165	1.22371674
1	-5.47637177	-0.28545833	-4.62698317	15	-1.09497178	3.20234156	0.59491676
1	-4.63627195	-1.84975839	-4.63858318	8	-0.88037169	3.26134157	-0.96508324
1	-3.85547185	-0.42165834	-5.31388330	8	0.15462831	0.41874167	-0.88438326
6	-6.40737152	-0.25505835	2.73711658	6	0.29312834	2.72604156	-1.65468323
1	-6.64747190	-1.30595839	2.93201661	6	0.11412831	1.26324165	-2.00838327
1	-7.28727150	0.22284167	2.29431653	1	1.17642832	2.89024162	-1.03248322
1	-6.22997189	0.22014166	3.70861673	1	0.37562832	3.33354163	-2.55828333
6	-4.87747192	1.42864168	1.77571678	1	-0.83677167	1.15224159	-2.54298329
1	-3.99637175	1.64604163	1.17401671	1	0.91152835	1.01214159	-2.71878338
1	-4.72337151	1.84374166	2.77971673	8	-2.38077164	4.09194183	0.83601671
1	-5.73387194	1.94384170	1.32671678	6	-2.52297163	5.39294195	0.23001671
6	-4.01087141	-0.79975837	2.60071659	1	-1.67287171	6.03144169	0.48331672
1	-4.23937178	-1.86325836	2.73471665	1	-2.61017179	5.29004192	-0.85268325
1	-3.85777164	-0.35965833	3.59371662	1	-3.43957162	5.81274176	0.64161676
1	-3.06777167	-0.73555839	2.05721664	1	2.03092837	4.71414185	3.85701656
12	1.66492832	-0.57485837	-0.08638328	1	2.59452820	4.24114180	2.24441671
8	0.20272832	-0.88795835	1.52131677	1	1.68652833	5.75244188	2.46121669
8	3.44372821	-0.04205834	-0.34238330	1	1.04252827	-0.12045834	4.73381662
6	-0.00417168	-0.49465832	2.90281653	1	1.73982823	-1.43645835	3.76821661
6	1.27312827	-0.45265833	3.71481657	1	1.99292827	0.25044167	3.28721666
1	-0.46267167	0.49814168	2.88261652	8	1.13192832	-2.47965837	-0.07898328

6	4.72692823	-0.25085834	-0.65778327	15	0.03622832	-2.75825834	0.94091672
6	5.15812826	-0.19375835	-2.02318335	8	-0.44387168	-4.30405807	0.55241674
6	6.49932814	-0.45645833	-2.31798339	8	-1.48917174	-2.16555834	0.55591673
1	6.83882809	-0.43285832	-3.34758329	6	-1.47697175	-4.29025793	-0.42458329
6	7.43152809	-0.74585837	-1.32978320	8	0.42512834	-3.11675835	2.45781660
6	7.01982832	-0.75175834	-0.00318328	6	0.53982830	-4.46145821	2.96451664
1	7.76312828	-0.95645839	0.75941676	1	-0.43337166	-4.95255804	2.97981668
6	5.69432831	-0.50425833	0.36701670	1	1.23932827	-5.04415798	2.36401653
6	4.20262814	0.18884166	-3.16818333	1	0.92212832	-4.34885836	3.97981668
6	5.31772804	-0.51995832	1.85861671	6	-2.39527178	-3.16735840	0.02581672
6	3.07322836	-0.84765834	-3.32138348	1	-2.97607183	-2.70785832	-0.77168328
1	2.47182822	-0.95215833	-2.41788340	1	-3.05867171	-3.47465825	0.83741677
1	2.40152836	-0.57365835	-4.14428329	1	-1.97327173	-5.26235819	-0.41778329
1	3.48872828	-1.83605838	-3.54268336	1	-1.05567169	-4.10035801	-1.41858327

DI-4b

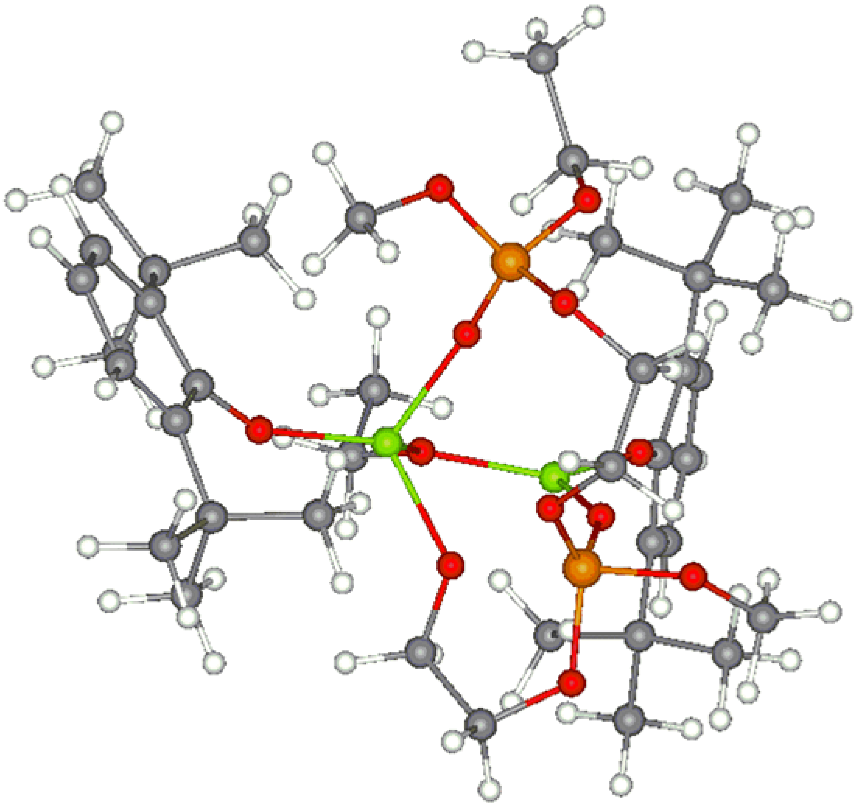

Zero-point vibrational energy	2676526.6 (Joules/Mol)
	639.70521 (Kcal/Mol)
Zero-point correction=	1.019435 (Hartree/Particle)
Thermal correction to Energy=	1.084350
Thermal correction to Enthalpy=	1.085294
Thermal correction to Gibbs Free Energy=	0.916106
Sum of electronic and zero-point Energies=	-3471.853971
Sum of electronic and thermal Energies=	-3471.789056
Sum of electronic and thermal Enthalpies=	-3471.788112
Sum of electronic and thermal Free Energies=	-3471.957300

cartesian

12	1.36022258	0.42852250	0.37905079	6	-3.39377737	-2.91677737	-0.98374921
8	0.61102259	-1.42517757	-3.68844914	1	-2.68607736	-2.12297750	-0.73714918
8	2.96222258	-0.01157750	1.29305077	1	-3.47877741	-3.57467747	-0.11234919
6	-0.17447741	-2.51357746	-3.11484909	1	-2.97797751	-3.50767732	-1.80994916
1	-1.18347740	-2.35227752	-3.49584913	6	-5.64167738	-3.54427743	-1.79254913
1	-0.19657741	-2.40557742	-2.02784920	1	-5.73207712	-4.29457760	-0.99984920
6	4.20472288	-0.48467749	1.20245075	1	-6.64857721	-3.23517752	-2.09154916
6	4.46082258	-1.88857758	1.35965085	1	-5.18237734	-4.03957748	-2.65554905

6	5.74572277	-2.37057734	1.08985090	6	-5.76807737	2.41482258	2.83885098
1	5.94882298	-3.43327737	1.16715074	1	-6.83067703	2.51762247	2.60075092
6	6.79232264	-1.52717757	0.73395079	1	-5.67497730	1.81462240	3.75065088
6	6.56882286	-0.15497752	0.71665078	1	-5.39127731	3.41932249	3.06125093
1	7.40992260	0.49662250	0.50555080	6	-3.47207737	1.83262241	2.09355092
6	5.30972290	0.40052250	0.97085077	1	-3.11007738	0.83642250	2.36895084
6	3.38412261	-2.86137748	1.87795091	1	-2.84247732	2.27802253	1.30915070
6	5.16082287	1.93432248	1.01115084	1	-3.31287742	2.47122264	2.96945095
6	2.21182251	-3.00857735	0.89015085	6	-5.16777706	2.74762249	0.45265079
1	1.66192257	-2.07767749	0.75495082	1	-6.23017740	2.81762266	0.19855082
1	1.50062263	-3.76147747	1.25185084	1	-4.80447721	3.75962257	0.67305082
1	2.57222247	-3.33277750	-0.09234919	1	-4.63427734	2.36872268	-0.42264920
6	2.88552260	-2.36567736	3.25075078	6	0.37902260	-3.85497761	-3.54404902
1	2.54072261	-1.33167756	3.20085096	1	-0.93837738	-0.05397750	3.11015081
1	3.69712257	-2.41197753	3.98505092	1	-8.05577755	-1.72257757	1.93565083
1	2.06582260	-2.99687743	3.61665082	1	7.77962303	-1.92977750	0.52065080
6	3.93602252	-4.28187752	2.09895086	8	1.31552255	-0.32927752	-1.48084927
1	4.77892303	-4.29347754	2.79725099	15	1.73352253	-0.66767746	-2.87924910
1	4.25412273	-4.75857735	1.16515088	8	2.09982252	0.56562251	-3.79754925
1	3.14752269	-4.90897751	2.52965093	8	1.33572257	2.77262259	-1.93104911
6	6.52422285	2.65152264	0.98215085	6	1.18472254	1.65582240	-4.08844948
1	7.05512285	2.51272249	0.03415081	6	1.58362257	2.90012264	-3.33304906
1	7.18002272	2.32432246	1.79565096	1	0.15562259	1.36742246	-3.86274910
1	6.36342287	3.72882247	1.10215092	1	1.27422261	1.82612240	-5.16394949
6	4.47542286	2.36672258	2.32295084	1	2.66252255	3.05302262	-3.41914916
1	3.51002264	1.87592244	2.45035076	1	1.06052256	3.76082253	-3.75054908
1	4.32892275	3.45432258	2.33405089	8	3.00282264	-1.59707749	-2.98574924
1	5.10092258	2.10562253	3.18365097	6	4.28952265	-1.20797753	-2.43334913
6	4.37282276	2.44702268	-0.20734920	1	4.64682293	-0.30997747	-2.94044924
1	4.86222267	2.13592243	-1.13734913	1	4.21522284	-1.04807758	-1.35734916
1	4.33172274	3.54352260	-0.20514919	1	4.95732260	-2.04277754	-2.63564920
1	3.34282255	2.09352255	-0.22604918	1	-0.26797742	-4.64927721	-3.15894914
12	-1.89937747	0.61232251	0.08005080	1	0.40822262	-3.93217754	-4.63374949
8	-0.42137742	-0.12107749	1.10285091	1	1.38722253	-4.01387739	-3.15494919
8	-3.65207744	0.08602250	-0.23404919	1	-1.58707738	-2.44347739	3.28435087
6	-0.56967741	-0.77567750	2.36525083	1	-2.51977754	-1.67777753	1.99205089
6	-1.50907743	-1.96857750	2.29995084	1	-1.13827741	-2.71287751	1.58935070
1	0.41372257	-1.10667753	2.71495080	8	-1.12507749	1.98962247	-1.01094913
6	-4.79997730	-0.35347748	0.28175080	15	-0.06557742	3.09872246	-1.17054915
6	-5.38367701	-1.56607759	-0.19584920	8	0.91562259	2.41072249	0.29535079
6	-6.54927731	-2.02687740	0.42365080	8	-0.18747742	4.47352266	-0.29184920

1	-7.00597715	-2.95367742	0.09285080	6	0.60952258	3.13032246	1.47125077
6	-7.15767717	-1.33157754	1.46395087	6	0.43692261	4.55752277	0.98805076
6	-6.62047720	-0.11607750	1.87405086	1	1.42812252	3.04142261	2.19075084
1	-7.12887716	0.42942250	2.66215086	1	-0.31367743	2.75302267	1.93595076
6	-5.46147728	0.40732250	1.29255080	1	-0.20817740	5.15502262	1.63585091
6	-4.77087736	-2.34737754	-1.37284923	1	1.40292263	5.05782270	0.86795086
6	-4.96827698	1.81672251	1.66765070	8	-0.70247746	3.86142254	-2.49744916
6	-4.64147711	-1.43557751	-2.61034918	6	-2.05717754	4.30182266	-2.48664904
1	-4.01787710	-0.56467748	-2.40394902	1	-2.73707747	3.46512246	-2.30134916
1	-4.20527697	-1.99347746	-3.44914913	1	-2.20937753	5.07882261	-1.73094916
1	-5.62757730	-1.07867754	-2.92704916	1	-2.25897741	4.72082281	-3.47514915

DI-5

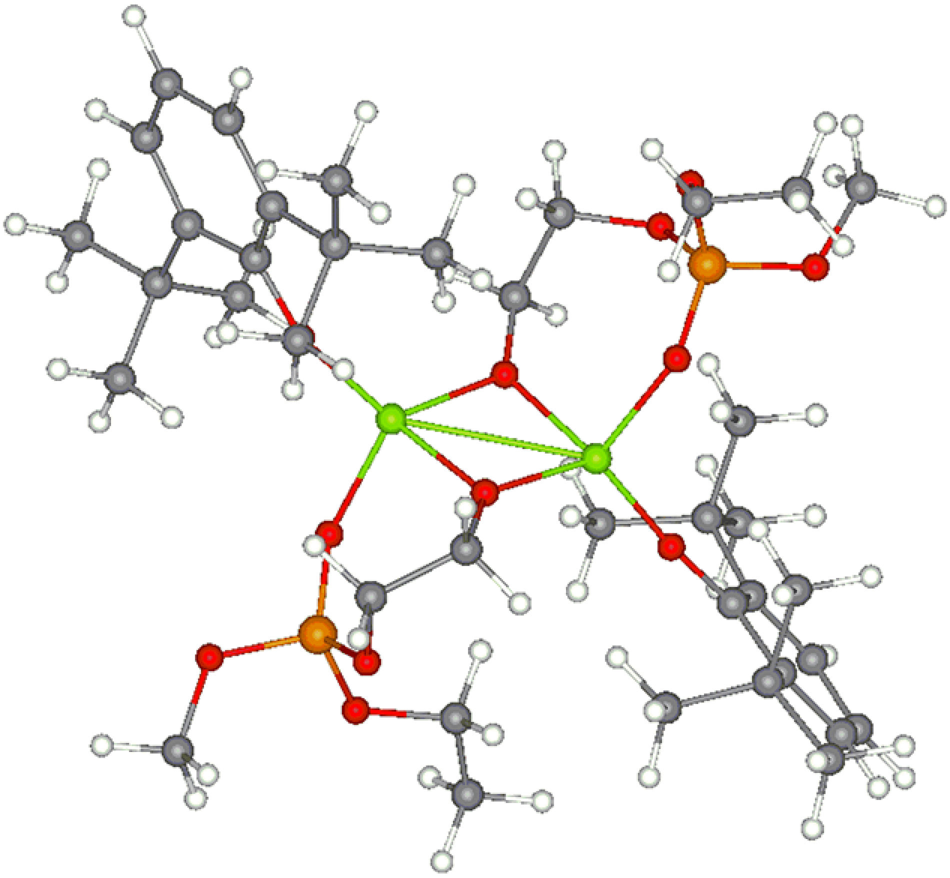

Zero-point vibrational energy	2673282.3 (Joules/Mol)
	638.92981 (Kcal/Mol)
Zero-point correction=	1.018199 (Hartree/Particle)
Thermal correction to Energy=	1.084123
Thermal correction to Enthalpy=	1.085067
Thermal correction to Gibbs Free Energy=	0.910990
Sum of electronic and zero-point Energies=	-3471.894056
Sum of electronic and thermal Energies=	-3471.828132
Sum of electronic and thermal Enthalpies=	-3471.827188
Sum of electronic and thermal Free Energies=	-3472.001265

cartesian

12	-1.14802015	-0.51941490	-0.23583333	6	6.03008032	1.88918507	3.18096662
8	1.77637982	4.94498539	-0.74133331	1	6.17727995	1.09858501	3.92486668
8	-2.90602016	0.06898512	0.06866667	1	6.99658012	2.10808516	2.71546674
6	1.53067982	6.15018511	-1.49263334	1	5.71748018	2.78908515	3.72246671
1	2.47347975	6.69538498	-1.49593329	6	5.08898020	-2.63271475	-2.77283335
1	0.75467992	6.74288511	-1.00483334	1	6.08428001	-2.27841473	-3.05983329

6	-4.19051981	0.34038511	0.26756668	1	5.20348024	-3.57931471	-2.23333335
6	-5.08262014	0.49518511	-0.84533334	1	4.54067993	-2.84951472	-3.69703341
6	-6.41411972	0.84238511	-0.59833336	6	2.93887997	-2.20481491	-1.64513338
1	-7.09982014	0.97218513	-1.42883337	1	2.28517985	-1.50971496	-1.11783338
6	-6.90341997	1.02848506	0.68936670	1	2.43367982	-2.50211477	-2.57303333
6	-6.04642010	0.83708513	1.76706672	1	3.06027985	-3.09871483	-1.02273333
1	-6.44961977	0.96388513	2.76616669	6	4.14788008	-0.35251489	-2.87263322
6	-4.70351982	0.48478511	1.59946668	1	5.12847996	0.02658512	-3.18033338
6	-4.61261988	0.28668511	-2.29643345	1	3.58867979	-0.61731488	-3.77953339
6	-3.83132005	0.22018512	2.84166670	1	3.62457991	0.45258513	-2.35523343
6	-4.07401991	-1.14551497	-2.48823333	1	7.91578007	-1.94671500	1.03216672
1	-3.28042006	-1.38981497	-1.78213334	1	-7.94242001	1.30498505	0.85176665
1	-3.68792009	-1.27851498	-3.50703335	8	1.18747985	2.72988510	0.27366668
1	-4.87771988	-1.87421489	-2.33683324	15	0.66847992	3.82228518	-0.60273331
6	-3.54062009	1.33098507	-2.65703344	8	0.23957989	3.34558511	-2.06083322
1	-2.70482016	1.29898500	-1.95683336	8	0.16097988	0.48568511	-1.33853328
1	-3.96882010	2.33948517	-2.62503338	6	0.96067989	2.29038525	-2.75953341
1	-3.16092014	1.16278505	-3.67313337	6	0.23457989	0.96078509	-2.65213323
6	-5.74921989	0.45898512	-3.32063341	1	1.98277986	2.22158527	-2.37113333
1	-6.17251968	1.46898508	-3.30913329	1	1.01327991	2.61338520	-3.80253339
1	-6.56201982	-0.25761488	-3.16273332	1	-0.76602006	1.08038509	-3.08593345
1	-5.35311985	0.28478512	-4.32783318	1	0.78707993	0.25968510	-3.29943323
6	-4.60542011	0.41528511	4.15826702	8	-0.59592009	4.61288500	-0.09053333
1	-5.46201992	-0.26161489	4.24276686	6	-1.89592016	3.95488524	0.07206667
1	-4.96532011	1.44268501	4.28006697	1	-2.20882010	3.58278513	-0.90513331
1	-3.93872023	0.20268512	5.00166702	1	-1.77052009	3.11158514	0.75276667
6	-2.62882018	1.18118501	2.89986658	6	-2.86452007	4.97328520	0.62246668
1	-1.97942019	1.07188511	2.03096676	8	0.18207988	-4.70161486	-0.22113334
1	-2.02962017	0.99288511	3.79956675	6	0.32537988	-4.95681477	-1.65043330
1	-2.97542024	2.21988511	2.94616675	1	1.36167991	-5.27791500	-1.76063335
6	-3.35322022	-1.24451494	2.82966661	1	0.19457988	-4.01341486	-2.18473339
1	-4.21072006	-1.92451489	2.87516665	6	-0.64112008	-6.01991463	-2.12983322
1	-2.71142006	-1.45081496	3.69536662	8	-1.11972010	-2.51501489	-0.58413333
1	-2.79692006	-1.47771490	1.92116666	15	-0.89272010	-3.67171478	0.33606666
12	1.52427983	0.74278510	0.10676667	8	-0.39302012	-3.34421492	1.79366672
8	3.36427975	0.41008511	-0.05273333	8	0.30637988	-0.42391488	1.13766670
6	4.54697990	-0.14561488	0.19166666	6	0.83457989	-2.60691476	2.09986663
6	5.34798002	0.30308512	1.29206669	6	0.55667990	-1.13121498	2.32146668
6	6.54048014	-0.37391487	1.56716669	1	1.56337988	-2.75901484	1.29996669
1	7.15307999	-0.06701488	2.40826678	1	1.20697987	-3.07071471	3.01586676
6	6.98758030	-1.43561494	0.78806669	1	-0.28422013	-1.03521490	3.02176666

6	6.24898005	-1.80791497	-0.32933334	1	1.44497991	-0.73411489	2.83436656
1	6.63407993	-2.60841489	-0.95233333	8	-2.25552011	-4.43831491	0.57806665
6	5.04648018	-1.17921495	-0.66603333	6	-2.35072017	-5.53331470	1.51166666
6	4.95458031	1.52848506	2.13986659	1	-1.61892009	-6.30841494	1.27106667
6	4.31207991	-1.58321500	-1.95813334	1	-2.19652009	-5.16821480	2.52836657
6	4.78957987	2.76378512	1.23146665	1	-3.35982013	-5.92811489	1.40416670
1	4.03808022	2.60088515	0.45916668	1	-0.44592011	-6.23091459	-3.18603325
1	4.48988008	3.63758516	1.82266665	1	-0.51192009	-6.94921494	-1.56853330
1	5.74007988	2.99838519	0.73936665	1	-1.67972016	-5.69171476	-2.03833342
6	3.66047978	1.26958501	2.93326664	1	1.23677981	5.91058540	-2.51753330
1	2.81467986	1.07168508	2.27316666	1	-3.84042025	4.49778509	0.75256670
1	3.77997994	0.40678513	3.59796667	1	-2.52902007	5.34848499	1.59256673
1	3.39987993	2.13978529	3.54696655	1	-2.97872019	5.81788540	-0.06243333

DI-1m2

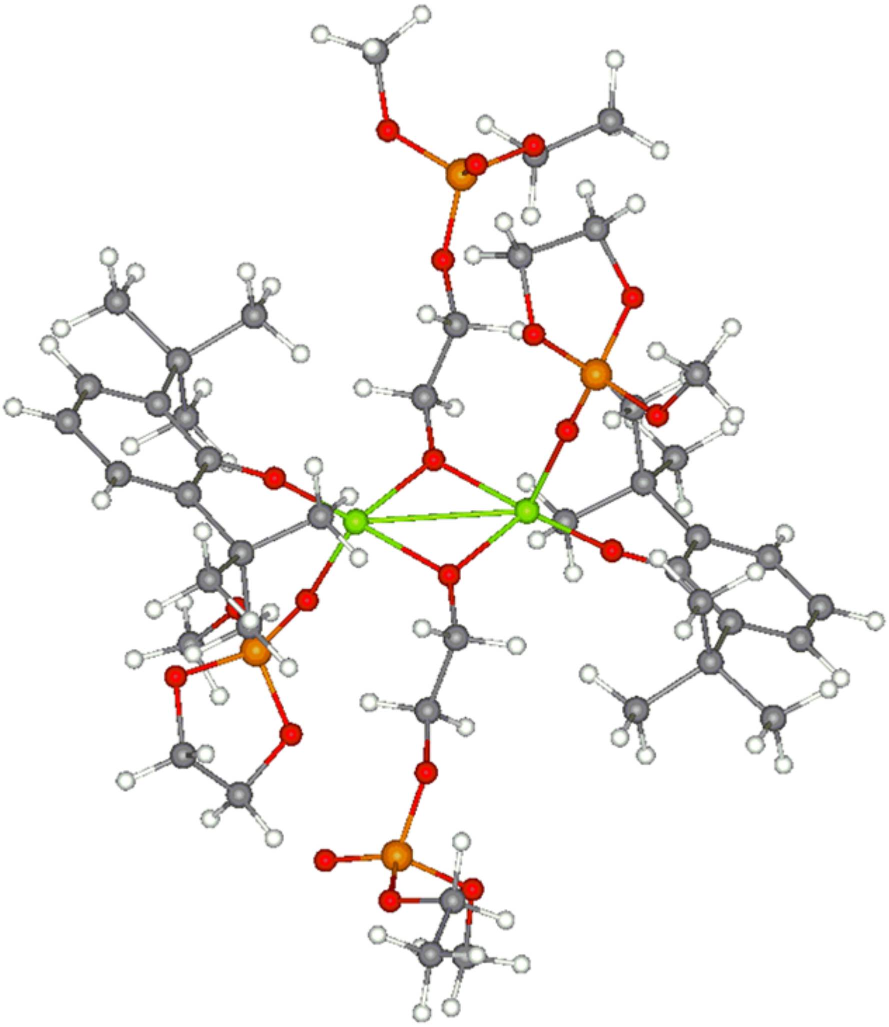

Zero-point vibrational energy	3285193.1 (Joules/Mol)
	785.17999 (Kcal/Mol)
Zero-point correction=	1.251264 (Hartree/Particle)
Thermal correction to Energy=	1.338420
Thermal correction to Enthalpy=	1.339365
Thermal correction to Gibbs Free Energy=	1.111674
Sum of electronic and zero-point Energies=	-4993.015820
Sum of electronic and thermal Energies=	-4992.928663
Sum of electronic and thermal Enthalpies=	-4992.927719
Sum of electronic and thermal Free Energies=	-4993.155410

cartesian

12	-0.48869064	1.40340960	-0.17518666	1	0.94410932	-2.98419046	-4.08458662
8	1.12910938	0.32810956	-0.56008667	6	3.34910941	-2.95539045	-1.55058670
8	-0.46909064	3.13330936	0.56261337	1	4.02230930	-3.71549058	-1.13838661

6	2.33440948	0.64530957	-1.20878661	1	3.96490932	-2.16109061	-1.98898661
6	3.46970940	0.65710956	-0.19498666	1	2.77940941	-2.53819060	-0.71808666
1	2.27500939	1.62730956	-1.69598663	1	2.56780934	-0.08979043	-1.98908663
6	-0.61229068	4.39600992	0.94651335	1	-2.48599052	0.04910957	2.19191337
6	0.16620934	5.43570948	0.33801335	1	1.03940940	-8.04549026	-2.10438657
6	-0.03509066	6.75670958	0.74781334	1	-1.08629060	8.13060951	2.03691339
1	0.53950930	7.55560970	0.29081336	8	-1.63209057	1.45420957	-1.86588669
6	-0.95089066	7.09380960	1.73811328	15	-2.41709065	1.49280953	-3.11988664
6	-1.68069065	6.08060980	2.34911323	8	-3.98029065	1.77970958	-2.91578674
1	-2.37999058	6.35570955	3.13181329	8	-2.53929067	0.13110957	-3.95708656
6	-1.53719068	4.73810959	1.98711336	6	-4.79549074	0.75080961	-3.53938675
6	1.23640943	5.12490988	-0.72288668	6	-3.86179066	-0.43979043	-3.76638675
6	-2.37429070	3.66490936	2.70471334	1	-5.18849087	1.16040957	-4.47408676
6	0.59920931	4.49680948	-1.97658670	1	-5.60089064	0.49410957	-2.85278654
1	0.01980934	3.60430956	-1.74208665	1	-3.84659052	-1.10369039	-2.90148664
1	1.37070942	4.22790956	-2.70958662	1	-4.10379076	-1.00449049	-4.66628647
1	-0.08289066	5.20940971	-2.45348668	8	-1.78949058	2.57420945	-4.08648682
6	2.29790950	4.18920994	-0.11418665	6	-2.37989068	2.86760950	-5.36678648
1	1.84210944	3.27460957	0.26761335	1	-2.38119054	1.97620952	-5.99848652
1	2.80260944	4.68440962	0.72271335	1	-3.39409065	3.25250936	-5.23598671
1	3.06070948	3.92620945	-0.85748667	1	-1.75149059	3.63650942	-5.81328678
6	1.98420942	6.38240957	-1.20038664	8	4.72470903	0.81310958	-0.91178668
1	2.50870943	6.88930988	-0.38338667	1	3.35300946	1.47870958	0.51631331
1	1.31800938	7.10560989	-1.68328667	1	3.49570942	-0.28879043	0.35021335
1	2.73840928	6.09060955	-1.94048667	8	-4.64599085	-1.00879049	1.03501332
6	-3.25699067	4.25000954	3.82191324	1	-3.56839061	0.43330956	-0.01558665
1	-3.99029040	4.97010994	3.44341326	1	-3.22839069	-1.25539041	-0.47268665
1	-2.66419053	4.73940992	4.60201311	8	1.67660940	-1.58589041	1.91791332
1	-3.81629062	3.43680954	4.29931355	15	2.50650930	-2.04089046	3.05671334
6	-1.45539057	2.62640953	3.37941337	8	3.63360929	-0.99399042	3.50651336
1	-0.80069065	2.13060951	2.66211343	8	3.45450950	-3.31229043	2.82291341
1	-2.05129051	1.86300957	3.89591336	6	4.95800924	-1.59129047	3.46291327
1	-0.82089067	3.11590958	4.12671328	6	4.81520891	-2.85139060	2.60791326
6	-3.32959056	2.99250937	1.70031333	1	5.25390911	-1.81559038	4.49161339
1	-4.03879070	3.72550941	1.30021334	1	5.63480902	-0.87459040	3.00011325
1	-3.90449071	2.19280958	2.18451333	1	4.95610905	-2.63489056	1.54751337
1	-2.78639054	2.57130957	0.85291332	1	5.48260927	-3.65509057	2.91761327
12	0.50590932	-1.37449038	0.26481333	8	1.56510937	-2.37469053	4.28031349
8	-1.08939064	-0.28169042	0.69971335	6	2.10450935	-2.80959058	5.54351330
8	0.42260936	-3.07279062	-0.54688668	1	2.69960928	-3.71589065	5.41121340
6	-2.28009057	-0.63359040	1.35751331	1	2.70330930	-2.01269054	5.99101353

6	-3.43009067	-0.58289039	0.36321333	1	1.24390936	-3.02219057	6.17551327
1	-2.22019053	-1.64739037	1.77401340	15	6.08540916	0.83190960	-0.08788665
6	0.56770933	-4.32889032	-0.95248663	8	7.19360924	0.75440961	-1.22988665
6	-0.25649065	-5.36929035	-0.40828666	8	6.10490894	2.34350944	0.46641335
6	-0.05509066	-6.68389034	-0.83858663	8	6.27810907	-0.20129043	0.95481336
1	-0.66419065	-7.48329020	-0.42998666	15	-5.97419071	-1.22029042	0.18221334
6	0.90480930	-7.01359034	-1.78868663	8	-6.99079084	-0.07019043	0.64101332
6	1.67980933	-5.99899054	-2.33848667	8	-5.83739090	-1.24179041	-1.29218662
1	2.41340947	-6.26749039	-3.09138656	8	-6.51309109	-2.57019043	0.86141336
6	1.53830934	-4.66249037	-1.95378661	6	7.00820923	2.69380951	1.52701330
6	-1.37059057	-5.06769037	0.60921335	6	7.15950918	1.64240956	-2.37598658
6	2.42370939	-3.58809042	-2.60888672	6	-7.21969080	0.20870958	2.04471326
6	-0.78069067	-4.48449039	1.90721333	6	-7.62649107	-3.24099064	0.24571334
1	-0.19009066	-3.58789063	1.71851337	1	-6.26769066	0.48700958	2.50571322
1	-1.57799065	-4.23139048	2.61731339	6	-8.22839069	1.33180964	2.13961339
1	-0.12169066	-5.21679020	2.38711333	1	-7.58629084	-0.70129043	2.53121328
6	-2.38869071	-4.09609032	-0.01808665	1	-7.77479076	-4.16099024	0.81061333
1	-1.89889061	-3.18109059	-0.35448664	1	-8.52969074	-2.62589049	0.30061334
1	-2.86519051	-4.56029034	-0.88888663	1	-7.40009069	-3.47539043	-0.79678667
1	-3.17689061	-3.83879066	0.70011336	1	6.76300907	3.71770954	1.80891335
6	-2.15929055	-6.32419014	1.01921332	1	6.87190914	2.02830935	2.38221335
1	-2.65259051	-6.80139017	0.16571334	1	8.04580975	2.64480948	1.18391335
1	-1.52719057	-7.07009029	1.51301336	6	8.41121006	1.39850962	-3.18948674
1	-2.94269061	-6.03879023	1.73081338	1	6.25660896	1.42560959	-2.95308661
6	3.34450936	-4.16219044	-3.70068669	1	7.10690928	2.67680955	-2.02168655
1	4.05010891	-4.90209007	-3.30788660	1	8.41030979	2.05030942	-4.06848669
1	2.77790928	-4.62439013	-4.51608658	1	9.30780983	1.61180961	-2.60158658
1	3.93650937	-3.34789062	-4.13398647	1	8.45710945	0.36060959	-3.52868676
6	1.55030942	-2.52059054	-3.29828668	1	-8.41709042	1.56750953	3.19131327
1	0.86960930	-2.03359056	-2.59938669	1	-7.85329103	2.23200941	1.64641333
1	2.17870927	-1.75419044	-3.76988673	1	-9.17559052	1.04810953	1.67331338

DI-6

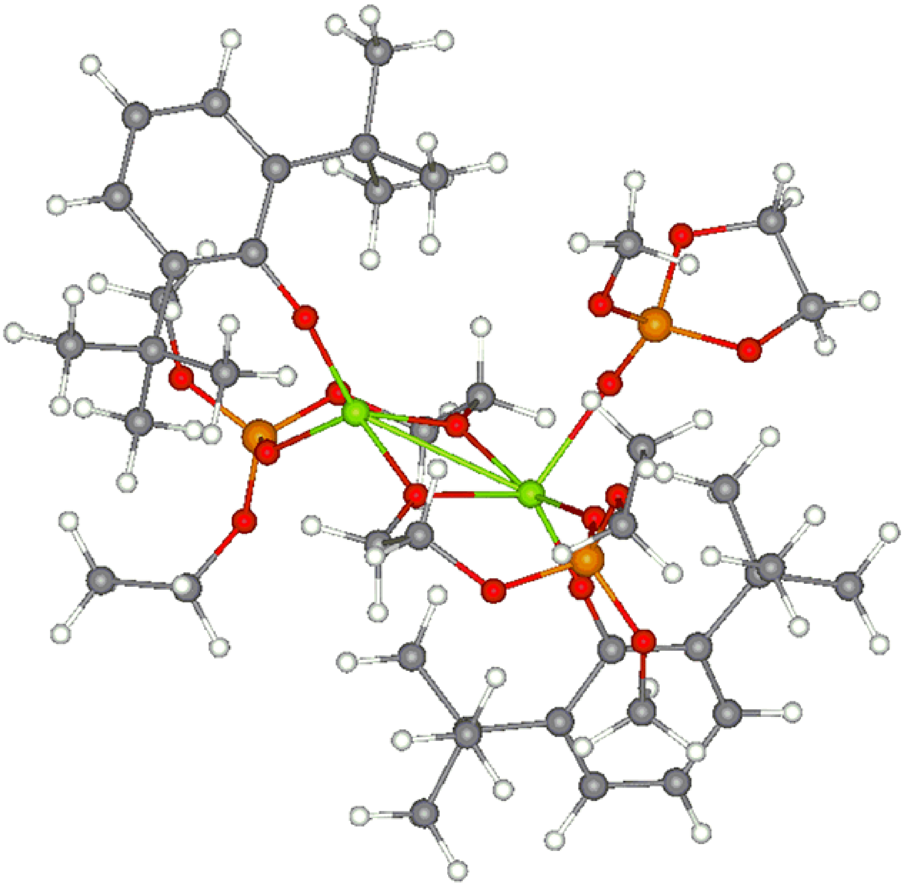

Zero-point vibrational energy	2980843.5 (Joules/Mol)
	712.43870 (Kcal/Mol)
Zero-point correction=	1.135343 (Hartree/Particle)
Thermal correction to Energy=	1.211051
Thermal correction to Enthalpy=	1.211996
Thermal correction to Gibbs Free Energy=	1.016741
Sum of electronic and zero-point Energies=	-4232.457906
Sum of electronic and thermal Energies=	-4232.382198
Sum of electronic and thermal Enthalpies=	-4232.381254
Sum of electronic and thermal Free Energies=	-4232.576508

cartesian

12	1.63710141	0.42053843	-0.10244668	6	-2.64319873	5.09163809	-2.13104677
8	2.24240136	4.69813824	0.73785335	1	-3.57459879	5.18583822	-2.69964671
8	3.09280133	-0.80566156	-0.15304667	1	-2.58329868	5.92993832	-1.42844665
6	2.42190123	5.32613802	-0.56374669	1	-1.81629860	5.20273829	-2.84244680
1	1.49970138	5.88543844	-0.72834665	6	-1.14649856	3.71553850	-0.73364669
1	2.49260139	4.54913807	-1.32924664	1	-0.96519858	2.77483845	-0.21464667
6	4.32870150	-1.26776159	-0.02244667	1	-0.34369859	3.85963845	-1.46754658

6	5.25450134	-1.20086169	-1.11874664	1	-1.08789861	4.52983809	-0.00174667
6	6.58340168	-1.57746160	-0.90044665	6	-2.56889868	2.66053843	-2.53384662
1	7.30300140	-1.50456166	-1.70934665	1	-3.52279878	2.71773839	-3.07064676
6	7.02470160	-2.06106162	0.32655331	1	-1.76389861	2.81953859	-3.26264668
6	6.09910154	-2.23256159	1.35035336	1	-2.46829867	1.65783834	-2.11684680
1	6.44380140	-2.66756153	2.28285336	6	3.63300133	6.23543835	-0.55754668
6	4.75460148	-1.87576163	1.20685339	1	1.22550142	0.08363841	-3.04324675
6	4.80770159	-0.78316158	-2.53244662	1	-6.43269825	5.08113813	0.77915335
6	3.76330137	-2.17956161	2.34595323	1	8.06640148	-2.33806157	0.47005332
6	4.39310169	0.69873840	-2.57554674	8	2.48790121	2.26493859	-0.09404667
1	3.61370134	0.93333840	-1.85204661	15	2.74110126	3.22703838	1.03075337
1	4.03170156	0.97143847	-3.57574677	8	2.08430123	2.84783840	2.41145325
1	5.25140142	1.34113836	-2.34734678	8	0.21370140	0.77543843	1.19785345
6	3.64650130	-1.69106162	-2.98714662	6	0.63500142	2.71973848	2.60755324
1	2.81460118	-1.65536165	-2.28224683	6	0.16350138	1.28083837	2.49645329
1	3.98600125	-2.73086143	-3.05524683	1	0.11760139	3.36133838	1.89205337
1	3.28950119	-1.38886166	-3.98054695	1	0.46810138	3.10223842	3.61745334
6	5.92510176	-0.95056158	-3.57854676	1	0.75110137	0.66723841	3.19585323
1	6.28890133	-1.98196161	-3.63494682	1	-0.86889857	1.28753841	2.87845325
1	6.78010130	-0.29346159	-3.38634682	8	4.28160143	3.39883852	1.35885334
1	5.53220129	-0.68646157	-4.56724691	6	5.15550137	2.24733853	1.46335340
6	4.40410137	-2.99996161	3.47975326	1	5.02480173	1.78053832	2.44035316
1	5.20640135	-2.45756149	3.99145317	1	4.95250130	1.52873838	0.66775334
1	4.80860138	-3.95196152	3.11995316	1	6.16890144	2.63443851	1.36705339
1	3.64030123	-3.22856140	4.23195314	1	3.70330119	6.74953842	-1.52164662
6	2.58870125	-3.02236152	1.80945337	1	3.54740119	6.98913813	0.22925334
1	2.06430125	-2.51676154	0.99875331	1	4.55470133	5.67053843	-0.39964667
1	1.87330139	-3.23006153	2.61515331	8	-1.75189865	-1.37356162	-3.50494671
1	2.95970130	-3.98106146	1.42815340	1	0.09280140	-1.43346167	-4.33214664
6	3.24150133	-0.88126159	2.98545337	1	0.02200139	-2.18606162	-2.72014666
1	4.06210136	-0.32346159	3.44995332	8	-1.47079861	-1.48736167	1.57415342
1	2.50360131	-1.10306168	3.76615334	15	-1.58269858	-2.94306159	1.78645337
1	2.76610136	-0.23266158	2.24915338	8	-3.05029869	-3.61506152	1.77345335
12	-1.36489856	0.04703841	0.10465333	8	-1.16469860	-3.40896153	3.27715325
8	0.11960139	0.02493841	-1.30474663	6	-3.49999881	-3.80186152	3.12905335
8	-2.80889869	1.32053840	0.22705333	6	-2.23359871	-4.11816168	3.93175316
6	0.18500140	-0.02796159	-2.69984674	1	-4.21889830	-4.62146187	3.13045335
6	-0.30109861	-1.32926166	-3.31854677	1	-3.98489881	-2.88416147	3.47185326
1	-0.38179860	0.79453844	-3.16144681	1	-2.29209876	-3.76616144	4.96265316
6	-3.72729874	2.26193857	0.35565332	1	-1.99729860	-5.18616199	3.92075324
6	-4.84019852	2.08573842	1.25145340	8	-0.71519858	-3.73726153	0.73465335

6	-5.77989864	3.11313844	1.37685335	6	-0.58469862	-5.16856194	0.78715330
1	-6.61859846	2.99623847	2.05495334	1	-0.11939861	-5.47106171	1.72825336
6	-5.68729830	4.29883814	0.65735334	1	-1.56119859	-5.64256191	0.66275334
6	-4.62759829	4.46063805	-0.22724667	1	0.06650140	-5.43766165	-0.04254666
1	-4.57439852	5.38613844	-0.79104668	15	-2.70959878	-1.93806159	-2.36844683
6	-3.64719868	3.48033857	-0.40734667	8	-2.44209862	-1.40686166	-1.00844657
6	-5.02839851	0.79743844	2.07535338	8	-4.15929842	-1.67416167	-2.95344663
6	-2.52019882	3.72863841	-1.42644656	8	-2.60549879	-3.52496147	-2.38534665
6	-5.15489864	-0.41976157	1.13995337	6	-4.71889830	-0.33796158	-2.96234679
1	-4.27339840	-0.54276156	0.51175332	6	-2.92949867	-4.27326155	-3.58694673
1	-5.29359865	-1.34156168	1.72045338	1	-5.77759838	-0.46276158	-3.18574667
1	-6.03149843	-0.30656159	0.49105334	1	-4.59419870	0.13563842	-1.98764670
6	-3.85169864	0.61143839	3.04995322	1	-4.23679829	0.25433841	-3.74164677
1	-2.90749860	0.50583839	2.51885319	6	-2.54889870	-5.71836185	-3.35604668
1	-3.77649879	1.47073841	3.72585320	1	-4.00049877	-4.16516161	-3.77754664
1	-3.99799871	-0.28596160	3.66685319	1	-2.37899876	-3.84646153	-4.43114662
6	-6.30629826	0.82553840	2.93385339	1	-2.79789877	-6.30786180	-4.24344683
1	-6.29209852	1.62773836	3.67945337	1	-1.47659862	-5.81616163	-3.16794682
1	-7.21259832	0.93303841	2.32825327	1	-3.09499860	-6.13206196	-2.50424671
1	-6.39159870	-0.12176158	3.47975326				

DTS-67

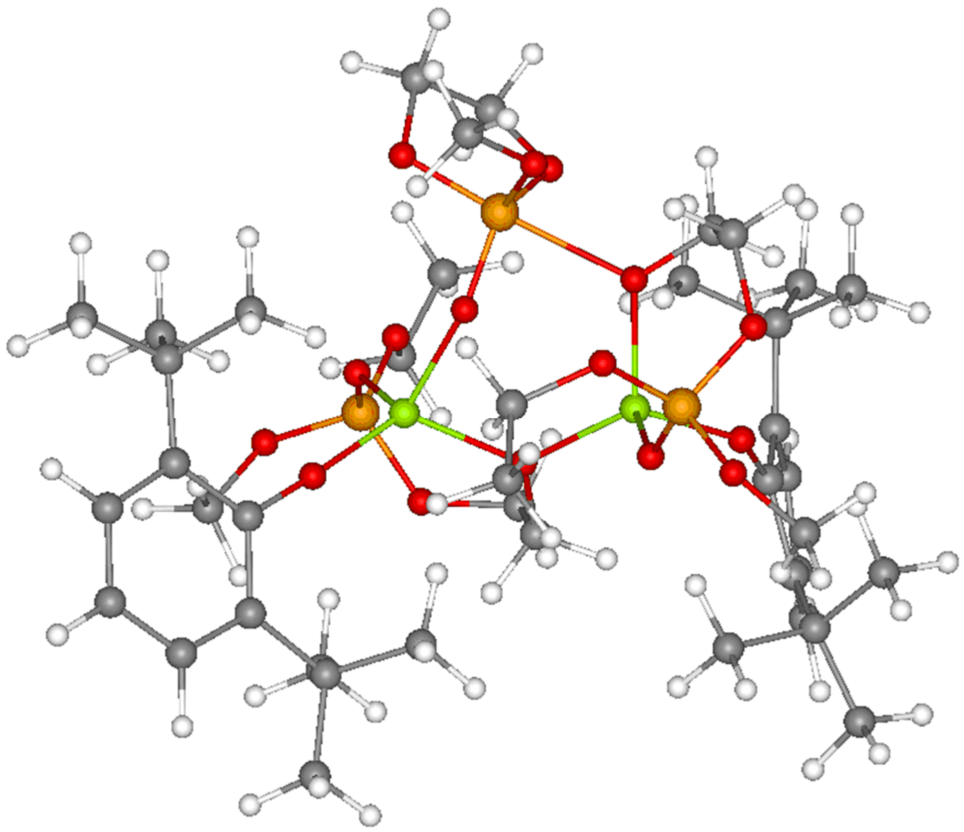

Zero-point vibrational energy	2980126.7 (Joules/Mol)
	712.26739 (Kcal/Mol)
Zero-point correction=	1.135070 (Hartree/Particle)
Thermal correction to Energy=	1.209745
Thermal correction to Enthalpy=	1.210690
Thermal correction to Gibbs Free Energy=	1.018171
Sum of electronic and zero-point Energies=	-4232.447059
Sum of electronic and thermal Energies=	-4232.372384
Sum of electronic and thermal Enthalpies=	-4232.371440
Sum of electronic and thermal Free Energies=	-4232.563958

cartesian

12	1.48326170	-0.91144741	-0.38568908	6	-3.44833827	-0.97094738	4.92311096
8	-0.48183829	-4.32634783	-1.44908917	1	-4.18983793	-0.32524741	5.40541124
8	3.31576157	-0.59634739	0.13391091	1	-3.80933833	-2.00394750	4.96841097
6	-1.74533820	-3.95264745	-0.81818908	1	-2.53333831	-0.91994739	5.52411127
1	-2.43703842	-3.84944725	-1.65468907	6	-2.03593826	-1.47934747	2.96621084
1	-1.62563825	-2.97534728	-0.34798908	1	-1.71163821	-1.22154737	1.95821095
6	4.09836197	0.22475258	0.82331091	1	-1.15843821	-1.45284748	3.62351084
6	4.42346191	-0.05354741	2.19471097	1	-2.40903831	-2.50944734	2.94871092

6	5.03416204	0.95315260	2.95081091
1	5.24156189	0.78605258	4.00281096
6	5.41106176	2.17225266	2.39541078
6	5.27296209	2.35015273	1.02181089
1	5.66776180	3.26075268	0.58201092
6	4.66896200	1.38915253	0.20431091
6	4.19666195	-1.44534731	2.82201099
6	4.71676207	1.55095267	-1.32848907
6	4.81716204	-2.51974726	1.90581083
1	4.35986185	-2.50704741	0.91511089
1	4.68566179	-3.51614738	2.34711099
1	5.89276171	-2.34774733	1.78861094
6	2.70766163	-1.75654745	3.05711079
1	2.14396167	-1.80834746	2.12541080
1	2.24626160	-1.00254738	3.70421076
1	2.59836173	-2.72624731	3.56041098
6	4.89346170	-1.57694745	4.18941116
1	4.45136166	-0.92554742	4.95111132
1	5.96446180	-1.35794735	4.12771130
1	4.78686190	-2.60664725	4.54891109
6	5.58886194	2.74555254	-1.75658917
1	6.60806179	2.67565274	-1.36288917
1	5.16576195	3.70715261	-1.44428909
1	5.65906191	2.76465273	-2.85018921
6	3.32806158	1.78105259	-1.94668913
1	2.65736151	0.93675256	-1.79418910
1	3.41366172	1.92525268	-3.03088903
1	2.86036158	2.68125272	-1.53108907
6	5.36236191	0.29105258	-1.94158912
1	6.39846182	0.18935259	-1.60028911
1	5.37136173	0.35835260	-3.03688908
1	4.82006168	-0.60944742	-1.64978909
12	-1.52193820	0.61995256	-0.20358908
8	0.29756171	0.40865257	0.53941095
8	-3.12943840	0.17675258	0.65261096
6	0.80746174	1.09315252	1.66511083
6	1.14196169	2.54945254	1.41061091
1	0.11046171	1.03205252	2.51161098
6	-4.29153824	-0.18664742	1.18391085
6	-5.48363829	-0.20424742	0.38701093
6	-6.68453836	-0.60004741	0.98461097

6	-2.59873843	0.91635257	3.58101082
1	-3.35773849	1.56955266	4.02691126
1	-1.70423830	0.95995259	4.21511126
1	-2.34903836	1.31375265	2.59581089
6	-2.21733832	-5.00534773	0.16131093
1	1.75036180	0.64195257	1.99671078
1	-7.70753813	-1.28094745	2.75681090
1	5.87016201	2.94235253	3.01061082
8	0.95346177	-2.81744742	0.10841091
15	0.91036177	-4.04244757	-0.75968909
8	1.97566164	-4.12494755	-1.92298913
8	1.26191235	-1.27968788	-2.32824111
6	1.89026177	-3.44714737	-3.21088910
6	2.20676160	-1.96654749	-3.10918903
1	0.89106172	-3.60834742	-3.61608911
1	2.62766171	-3.96214724	-3.83168912
1	3.21446157	-1.84284735	-2.68918920
1	2.23676157	-1.56934738	-4.13498878
8	1.19926178	-5.36054754	0.08031093
6	2.33076167	-5.41584778	0.97321099
1	3.25946164	-5.44554758	0.39901096
1	2.32786155	-4.55554771	1.64401090
1	2.21676159	-6.33774757	1.54251087
1	-3.20513844	-4.72084761	0.53621095
1	-2.29493833	-5.98274755	-0.32188907
1	-1.54173827	-5.09074736	1.01561093
8	-0.03663829	3.43135262	1.42621088
1	1.76846170	2.93585277	2.21611094
1	1.67126179	2.66745257	0.46281093
8	-1.03193831	-0.38634741	-1.82488906
15	-0.49643829	-0.33214742	-3.23598909
8	0.68796176	0.64705259	-3.75308919
8	-1.69853830	0.52985257	-3.99788904
6	0.19806170	1.77745247	-4.50128889
6	-1.16453826	1.35045266	-5.02908897
1	0.92366177	1.98805261	-5.28838873
1	0.12296171	2.62985253	-3.82128906
1	-1.84023821	2.19355273	-5.18558884
1	-1.07143831	0.78155261	-5.96378899
8	-0.54723823	-1.69174743	-4.09378910
6	-1.80353820	-2.16994739	-4.61358881

1	-7.59643793	-0.62114739	0.39741093	1	-2.57873845	-2.15624738	-3.84548903
6	-6.75943804	-0.97504741	2.32091093	1	-2.12543845	-1.56864738	-5.46648884
6	-5.60303831	-0.94864744	3.09191084	1	-1.61693823	-3.19654727	-4.93068886
1	-5.67723799	-1.24014735	4.13411093	15	-0.88483828	3.78055263	0.13721092
6	-4.36693811	-0.56094742	2.56621099	8	-1.45563829	2.59875274	-0.57828909
6	-5.47313833	0.18195258	-1.10358906	8	-1.95723832	4.81285238	0.67811096
6	-3.13103843	-0.52654743	3.48351097	8	-0.00343828	4.63745260	-0.85938907
6	-4.98293829	1.63135266	-1.29248917	6	-3.00843835	4.37825251	1.57591093
1	-3.98753834	1.78965259	-0.87838906	6	0.60226172	5.89525223	-0.44068909
1	-4.95213795	1.89005256	-2.35788918	1	-3.68543839	5.22605228	1.66921091
1	-5.66893816	2.32845259	-0.79718906	1	-3.53723836	3.52165270	1.15531087
6	-4.58973837	-0.80254740	-1.89488912	1	-2.58203840	4.12755251	2.54851079
1	-3.57193828	-0.83984739	-1.50608909	6	1.46176171	6.39735222	-1.57778907
1	-5.01343822	-1.81214738	-1.83728909	1	-0.20243829	6.59505224	-0.20138907
1	-4.54083824	-0.51034743	-2.95098901	1	1.19506168	5.71605253	0.46111095
6	-6.87313795	0.11785258	-1.74098909	1	1.92546177	7.34535217	-1.28968906
1	-7.29983807	-0.89044744	-1.71288908	1	2.25426173	5.68355227	-1.81448913
1	-7.57953835	0.80425256	-1.26178908	1	0.86056173	6.56635237	-2.47458911
1	-6.79993820	0.40905258	-2.79518914				

DI-7

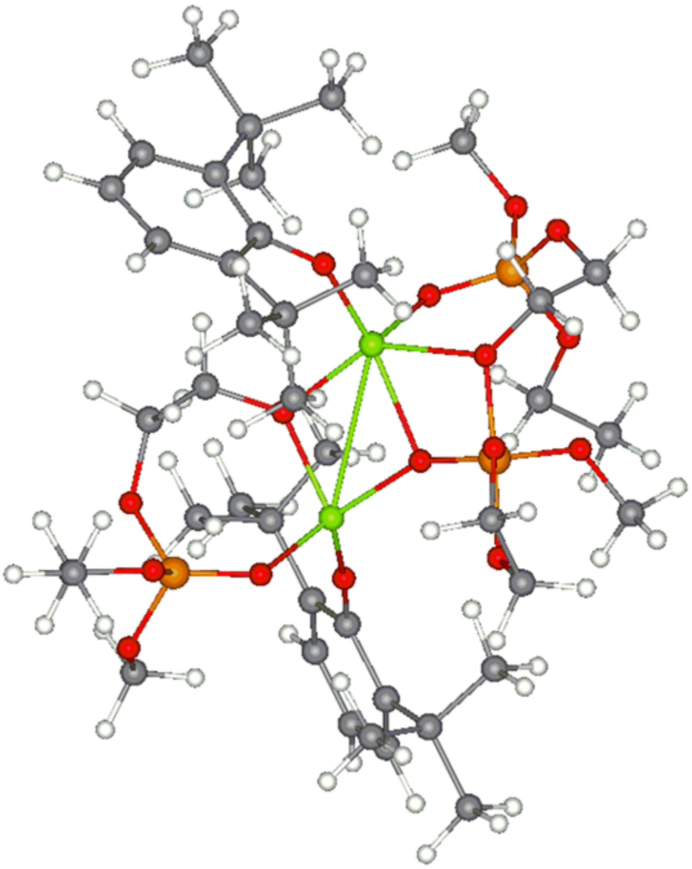

Zero-point vibrational energy	2984866.0 (Joules/Mol)
	713.40010 (Kcal/Mol)
Zero-point correction=	1.136875 (Hartree/Particle)
Thermal correction to Energy=	1.211505
Thermal correction to Enthalpy=	1.212449
Thermal correction to Gibbs Free Energy=	1.022206
Sum of electronic and zero-point Energies=	-4232.462257
Sum of electronic and thermal Energies=	-4232.387627
Sum of electronic and thermal Enthalpies=	-4232.386683
Sum of electronic and thermal Free Energies=	-4232.576926

cartesian

12	1.42370617	-0.85265636	-0.10319195	6	-3.78189373	-0.68795633	4.79710770
8	-0.21909384	-4.62775612	-0.28969195	1	-4.44879389	0.07544366	5.21160793
8	3.25430632	-0.28815633	0.07370805	1	-4.29029417	-1.65645635	4.85270786
6	-1.48999393	-4.18545628	0.28010803	1	-2.90519381	-0.74205637	5.45240784
1	-1.70309389	-3.19405627	-0.12319195	6	-2.32289386	-1.45725632	2.96610808
1	-1.38789392	-4.10765648	1.36510813	1	-1.92139387	-1.29815638	1.96590805
6	4.11670589	0.63154364	0.48500803	1	-1.48229384	-1.49295640	3.66960812
6	4.77520609	0.49944368	1.75560808	1	-2.82059383	-2.43385625	2.98610806
6	5.51210594	1.58334363	2.24400806	6	-2.61939383	1.01404369	3.44380808

1	5.97870588	1.52044368	3.22150803	1	-3.32199383	1.77894366	3.79390812
6	5.69220591	2.75024366	1.50870812	1	-1.78009391	0.98184359	4.14920807
6	5.19790602	2.80134368	0.20950803	1	-2.23939371	1.32334363	2.46930814
1	5.42510605	3.67804384	-0.38879195	6	-2.54909372	-5.19435644	-0.09619195
6	4.44690609	1.75914359	-0.34409195	1	1.82560611	1.00024366	2.22440815
6	4.74720621	-0.81585634	2.56130815	1	-7.90509415	-0.53045630	2.38780808
6	4.05730581	1.81644368	-1.83459187	1	6.26140594	3.58104372	1.91880798
6	5.14960623	-1.99195635	1.64800811	8	1.15200615	-2.68295622	0.77490807
1	4.46600580	-2.09245634	0.80400807	15	1.14820611	-4.10565615	0.30310804
1	5.15410614	-2.93015623	2.21760798	8	2.22820616	-4.48275614	-0.79229194
1	6.16000605	-1.83895636	1.25300813	8	1.46000612	-1.90315640	-1.98539197
6	3.36580634	-1.08025634	3.18630815	6	2.08890629	-4.24005604	-2.21739197
1	2.59520626	-1.24275637	2.43140817	6	2.37760615	-2.80085635	-2.59229183
1	3.06040621	-0.24265634	3.82320809	1	1.08710611	-4.53735638	-2.52599192
1	3.40340614	-1.97285640	3.82470798	1	2.82840633	-4.89785624	-2.68029189
6	5.75710583	-0.80065632	3.72430801	1	3.37850618	-2.51815629	-2.25419188
1	5.49550581	-0.07215634	4.49930763	1	2.34890628	-2.70815635	-3.68319201
1	6.77550602	-0.58935630	3.38220811	8	1.44660616	-5.12305641	1.48390806
1	5.76770592	-1.78645635	4.20330763	6	2.51030612	-4.88175631	2.42690802
6	4.75590611	2.97154379	-2.57589197	1	3.47670627	-5.04995632	1.94720817
1	5.84600592	2.91464376	-2.49059200	1	2.45020628	-3.86465621	2.81500816
1	4.43500614	3.95674372	-2.21999192	1	2.36420631	-5.60165644	3.23150802
1	4.50470591	2.91814375	-3.64149189	1	-3.51389384	-4.86475611	0.29940805
6	2.54660630	2.03224373	-2.02109194	1	-2.63729382	-5.28545618	-1.18149197
1	1.95260608	1.26964366	-1.51669192	1	-2.31809378	-6.17815638	0.32010806
1	2.28560615	2.00854373	-3.08529186	8	-0.25279382	3.52174377	1.41030812
1	2.24040627	3.00804377	-1.62929189	1	1.55600607	3.32004380	2.32260799
6	4.50380611	0.51824367	-2.53539181	1	1.58280611	2.87834382	0.60170805
1	5.59270620	0.41134366	-2.47649193	8	-0.31529382	-0.64395630	-1.35179198
1	4.21880579	0.53684366	-3.59449196	15	0.00400617	-1.26295638	-2.74669194
1	4.04800606	-0.35355633	-2.06799197	8	1.00190616	-0.60265636	-3.87689185
12	-1.34779394	0.58464360	-0.16479194	8	-1.34779394	-0.54285634	-3.49139190
8	0.32910618	0.52984369	0.88390809	6	0.37570617	0.26914367	-4.82059193
8	-3.05849385	0.14984366	0.49050805	6	-1.09759390	-0.11675633	-4.81369209
6	0.82750618	1.33794367	1.91580796	1	0.85970616	0.12104367	-5.78829193
6	1.01860607	2.79154372	1.53250813	1	0.52390617	1.30074358	-4.48859215
1	0.18260616	1.28974366	2.80410814	1	-1.75019383	0.72934365	-5.04209232
6	-4.28379393	-0.04445634	0.96710813	1	-1.30029392	-0.92145634	-5.53539228
6	-5.41919422	-0.01905634	0.08960805	8	-0.44859383	-2.80195618	-3.03609204
6	-6.69559383	-0.19055635	0.63460809	6	-1.67339385	-3.19285631	-3.66909194
1	-7.56529379	-0.16525635	-0.01289195	1	-2.53599381	-2.71665621	-3.20469189

6	-6.90019417	-0.40085635	1.99300814	1	-1.65659392	-2.96135616	-4.73759222
6	-5.79689407	-0.45355633	2.83610797	1	-1.73049390	-4.27605629	-3.54489183
1	-5.97129393	-0.63165629	3.89170814	15	-0.97419381	3.76484370	0.02110805
6	-4.49019384	-0.28595632	2.36690807	8	-1.31309390	2.51694369	-0.73069191
6	-5.26969385	0.16234367	-1.43209195	8	-2.21059370	4.67464352	0.40920803
6	-3.32069373	-0.35395634	3.36670804	8	-0.08589384	4.70844364	-0.88459194
6	-4.65029383	1.53164363	-1.76949191	6	-3.29239368	4.15964365	1.22420812
1	-3.67079377	1.67024362	-1.31299198	6	0.33180618	6.02644396	-0.42699197
1	-4.53469419	1.64444363	-2.85459185	1	-4.06289387	4.92904377	1.20990813
1	-5.30419397	2.33874369	-1.41909194	1	-3.68319368	3.23274374	0.80200809
6	-4.41399384	-0.98105633	-2.00919199	1	-2.93919373	3.99794388	2.24360800
1	-3.42879367	-1.01885629	-1.54379189	6	1.10660613	6.68064356	-1.54759192
1	-4.91169405	-1.94325638	-1.84039187	1	-0.56189382	6.59954357	-0.16659194
1	-4.27389383	-0.84985638	-3.08849192	1	0.94780618	5.90344381	0.46860805
6	-6.62049389	0.10794365	-2.16959190	1	1.43570614	7.67394352	-1.22849190
1	-7.13599396	-0.84775639	-2.02689195	1	1.98880613	6.09194374	-1.80929196
1	-7.29629421	0.91344368	-1.86249197	1	0.48260617	6.79194355	-2.43779182
1	-6.44559383	0.22554365	-3.24519181				

DTS-78

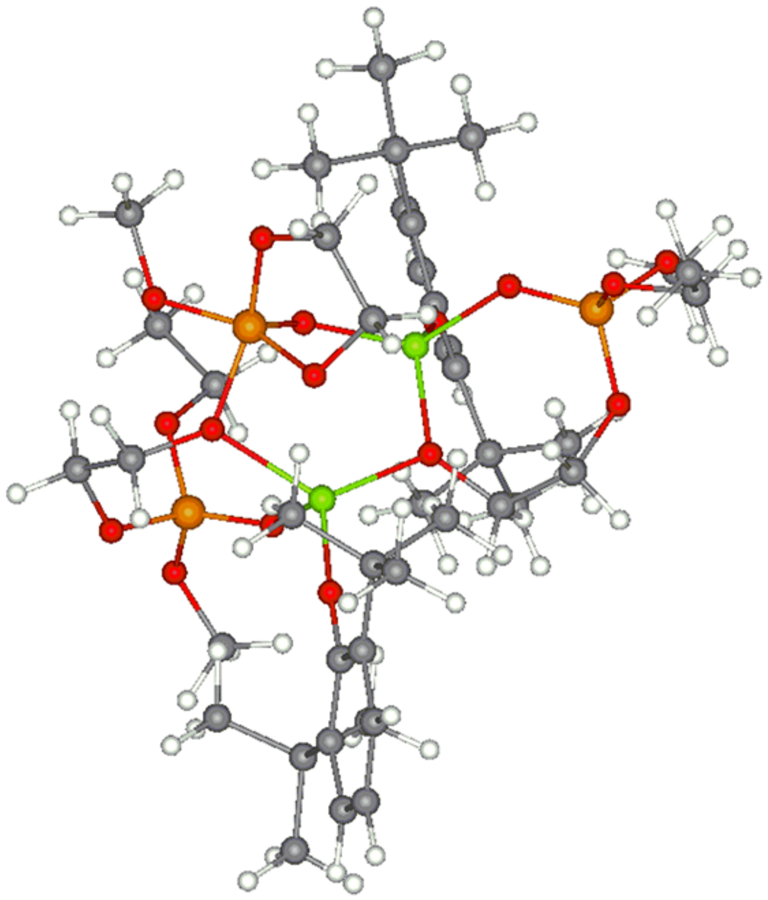

Zero-point vibrational energy 2985272.5 (Joules/Mol)

	713.49726 (Kcal/Mol)
Zero-point correction=	1.137030 (Hartree/Particle)
Thermal correction to Energy=	1.210878
Thermal correction to Enthalpy=	1.211822
Thermal correction to Gibbs Free Energy=	1.025295
Sum of electronic and zero-point Energies=	-4232.442930
Sum of electronic and thermal Energies=	-4232.369082
Sum of electronic and thermal Enthalpies=	-4232.368138
Sum of electronic and thermal Free Energies=	-4232.554665

cartesian

12	1.64996374	-0.42086893	-0.40770584	6	-3.51003647	-1.99146891	4.37699413
8	-0.86923635	-3.51226878	-1.57580578	1	-4.41643620	-1.58826888	4.84079409
8	3.45936370	-0.25196892	0.14969414	1	-3.65123630	-3.06676888	4.22439384
6	-2.20643640	-3.32756877	-1.01800585	1	-2.69273639	-1.87276888	5.09749413
1	-2.39263630	-2.25546885	-0.95570588	6	-1.84573627	-1.90036893	2.55799413
1	-2.23293638	-3.75526881	-0.01300585	1	-1.48483622	-1.43126893	1.64299417
6	4.69826365	-0.10866892	0.61729419	1	-1.05183625	-1.82626891	3.31179404
6	5.24686384	-1.05666888	1.54579413	1	-2.01033640	-2.96436882	2.35349417
6	6.54016352	-0.85616893	2.03839397	6	-2.88803649	0.21253109	3.46509409
1	6.96096373	-1.56116891	2.74669409	1	-3.79633641	0.65603107	3.88899422
6	7.32416344	0.22253110	1.65139413	1	-2.09743643	0.27813107	4.22329426
6	6.80376339	1.12693107	0.73519409	1	-2.59023643	0.80473107	2.59939408
1	7.42916346	1.95953107	0.43229416	6	-3.18913627	-4.02446890	-1.92850578
6	5.51776361	0.99563110	0.20219415	1	1.69996369	0.92613107	2.11409402
6	4.46756363	-2.30206871	2.00289416	1	-7.35533619	-2.75636888	1.64909422
6	5.02656364	2.04843116	-0.80640590	1	8.32676315	0.35223109	2.05159402
6	4.15136385	-3.18426871	0.78149414	8	0.74766368	-2.24816871	0.08419415
1	3.54876351	-2.65156889	0.04659414	15	0.43206367	-3.49746871	-0.68590581
1	3.61196351	-4.08996868	1.08199418	8	1.55246377	-3.95396876	-1.71460581
1	5.08076382	-3.49836874	0.29389414	8	1.39516377	-1.12156892	-2.37240601
6	3.18396354	-1.89266896	2.74909401	6	1.64266372	-3.43236876	-3.06270599
1	2.49176359	-1.35256898	2.10369396	6	2.22766352	-2.03806877	-3.08740592
1	3.42946362	-1.24896896	3.60119414	1	0.65396369	-3.44586873	-3.52190590
1	2.66606355	-2.77656889	3.14079404	1	2.30606365	-4.12356901	-3.58770585
6	5.27116346	-3.18026876	2.97959399	1	3.20726371	-2.02416873	-2.59950590
1	5.52156353	-2.65256882	3.90619421	1	2.34876370	-1.72326887	-4.12690592
1	6.19836378	-3.55676889	2.53529406	8	0.24776369	-4.75106859	0.26859415
1	4.66746378	-4.05216885	3.25679398	6	0.72766370	-4.81076860	1.62459421
6	6.08206367	3.12903118	-1.10420585	1	1.67836368	-5.34636879	1.63929415
1	6.99556351	2.70973110	-1.53820586	1	0.84746367	-3.80926871	2.03699398

1	6.35616350	3.70413113	-0.21310586
1	5.67256355	3.83683109	-1.83450580
6	3.80476356	2.78483129	-0.22780585
1	2.97046351	2.10033131	-0.07180586
1	3.48016357	3.58943129	-0.90000582
1	4.06246376	3.23903108	0.73599410
6	4.69296360	1.38143110	-2.15410590
1	5.59206343	0.91523105	-2.57230592
1	4.33476353	2.12503123	-2.87770605
1	3.92606354	0.61583108	-2.04790592
12	-1.46673632	0.68713105	-0.28350586
8	0.32806367	0.71313107	0.58139420
8	-3.07583642	0.01953109	0.43979415
6	0.67106366	1.21583104	1.85049427
6	0.65256369	2.73083115	1.94999421
1	0.01636369	0.80593109	2.63249397
6	-4.18443632	-0.63276893	0.76799417
6	-5.32103634	-0.63466895	-0.11020586
6	-6.43023634	-1.41356897	0.23769414
1	-7.29253626	-1.44516897	-0.41920587
6	-6.48083639	-2.15516877	1.41219413
6	-5.40873623	-2.08826876	2.29419398
1	-5.47923660	-2.64156890	3.22449398
6	-4.26443624	-1.33336890	2.01879406
6	-5.37303638	0.20543109	-1.40370584
6	-3.14343643	-1.25586891	3.07469416
6	-5.11603642	1.69283104	-1.09170592
1	-4.13493633	1.85123110	-0.64430583
1	-5.16933632	2.28963113	-2.01070595
1	-5.87803650	2.07383108	-0.40160584
6	-4.35503626	-0.30196893	-2.44170594
1	-3.32953644	-0.28656894	-2.07360578
1	-4.59193659	-1.33056891	-2.73500586
1	-4.39233637	0.31903109	-3.34590602
6	-6.75303650	0.14243110	-2.08490586
1	-7.00683641	-0.86726892	-2.42490602
1	-7.55603647	0.49783105	-1.43030584
1	-6.74373627	0.78643107	-2.97140598
1	-0.02063631	-5.36536884	2.19149399
1	-4.20093632	-3.86786890	-1.54440582
1	-3.13603640	-3.61736870	-2.94120598
1	-2.99073648	-5.09906864	-1.96940589
8	-0.69603634	3.29883122	2.03369403
1	1.12786376	3.04513121	2.88199401
1	1.18406379	3.18503118	1.10989416
8	-0.86923635	-0.06126891	-1.99710584
15	0.24586369	0.02513109	-3.04700589
8	1.37206376	1.11293113	-2.32240605
8	-0.13843630	1.22593105	-4.14660597
6	0.98106366	2.46853113	-2.48220587
6	0.31866369	2.53523111	-3.85920596
1	1.87416375	3.09083128	-2.42440605
1	0.28496367	2.75323129	-1.68870580
1	-0.53003633	3.22413111	-3.86680603
1	1.02856374	2.82813120	-4.64040613
8	-0.01353631	-1.11016893	-4.20630598
6	-0.97523630	-0.95416892	-5.25510597
1	-1.92523623	-0.57906890	-4.86780596
1	-0.60433632	-0.27916893	-6.02850580
1	-1.12173629	-1.95356894	-5.67170572
15	-1.50723624	3.77013111	0.75309420
8	-1.72873628	2.70253110	-0.26810586
8	-2.80853629	4.42193127	1.36879420
8	-0.75713634	4.99183130	0.08159414
6	-3.76113629	3.64013124	2.13319397
6	-0.51643634	6.22913122	0.81219411
1	-4.61623621	4.29533100	2.29189396
1	-4.06573629	2.75533128	1.57319415
1	-3.31693649	3.35753131	3.08849406
6	0.33546367	7.12393141	-0.05810586
1	-1.48523629	6.68293142	1.03489423
1	-0.01433631	5.98923111	1.75429416
1	0.51956367	8.06773090	0.46349415
1	1.29916370	6.65743113	-0.27670586
1	-0.17033631	7.34413099	-1.00130582

DI-8

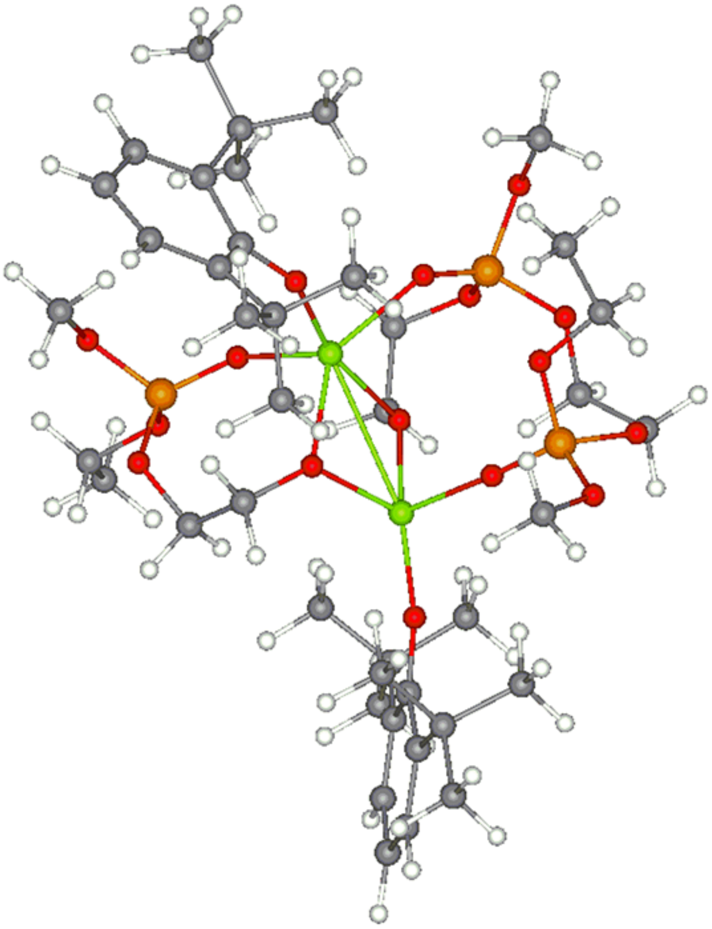

Zero-point vibrational energy	2981995.1 (Joules/Mol)
	712.71393 (Kcal/Mol)
Zero-point correction=	1.135782 (Hartree/Particle)
Thermal correction to Energy=	1.211045
Thermal correction to Enthalpy=	1.211990
Thermal correction to Gibbs Free Energy=	1.020143
Sum of electronic and zero-point Energies=	-4232.438967
Sum of electronic and thermal Energies=	-4232.363704
Sum of electronic and thermal Enthalpies=	-4232.362760
Sum of electronic and thermal Free Energies=	-4232.554607

cartesian

12	-1.74398005	-0.23436901	0.14263910	6	3.79741979	-1.71646893	-4.57186079
8	0.50751990	-3.93466902	0.25553912	1	3.64251995	-0.92576897	-5.31416082
8	-3.58428001	0.09163100	-0.06926090	1	4.86251974	-1.97066903	-4.55786085
6	1.07331991	-5.22046900	0.64103913	1	3.25051999	-2.59816909	-4.92606068
1	0.49471989	-6.02076864	0.16763911	6	3.42082000	-2.57206917	-2.28116083
1	0.97091991	-5.31436872	1.72533917	1	3.03792000	-2.37876916	-1.27776086
6	-4.80008030	0.50373101	-0.41906092	1	2.86301994	-3.41286898	-2.71476078
6	-5.45637989	-0.05426900	-1.56496084	1	4.47241974	-2.86956906	-2.20146084
6	-6.69748020	0.46343097	-1.94796085	6	1.78381991	-0.99446899	-3.33586097

1	-7.19867992	0.06883100	-2.82526088	1	1.65171993	-0.12846899	-3.99406099
6	-7.33028030	1.47463107	-1.23496091	1	1.25271988	-1.83916903	-3.79596090
6	-6.72478008	1.96173108	-0.08286089	1	1.31601989	-0.78286898	-2.37466097
1	-7.24738026	2.72483087	0.48393911	6	2.52061987	-5.25806904	0.21043912
6	-5.48218012	1.49653101	0.35843909	1	-1.10928011	0.96943104	-2.77426076
6	-4.85768032	-1.23396897	-2.35266089	1	6.71352005	1.86323106	-3.40106082
6	-4.89058018	2.04983091	1.66703916	1	-8.29297924	1.86153102	-1.55996084
6	-4.67377996	-2.43716908	-1.40736091	8	-1.36638010	-2.24946904	0.17703912
1	-4.00318003	-2.19986916	-0.58056092	15	-1.05538011	-3.69246912	0.36453912
1	-4.26957989	-3.29956913	-1.95126081	8	-1.54918015	-4.36286879	1.70863914
1	-5.63988018	-2.73546910	-0.98556089	8	0.22121991	-2.69386911	3.31063914
6	-3.52158022	-0.84386897	-3.01056075	6	-1.91578007	-3.73766899	2.95813918
1	-2.76808000	-0.57106900	-2.27096081	6	-1.19868016	-2.45346904	3.29623914
1	-3.65878010	0.01273100	-3.67946076	1	-1.70888007	-4.50356865	3.70763922
1	-3.12588000	-1.67346895	-3.61016083	1	-2.99158001	-3.54166913	2.93713903
6	-5.77688026	-1.70876896	-3.49266076	1	-1.42128015	-1.65106893	2.59203911
1	-5.93738031	-0.93496895	-4.25116110	1	-1.52308011	-2.14796901	4.29703903
1	-6.75377989	-2.04026914	-3.12596083	8	-1.74718010	-4.67196894	-0.67766094
1	-5.31158018	-2.56396914	-3.99626064	6	-1.46468008	-4.49666882	-2.08436084
6	-5.84248018	3.02623081	2.38173914	1	-2.08398008	-5.22586870	-2.60416079
1	-6.79938030	2.55853081	2.63603926	1	-1.73028016	-3.48756909	-2.40296078
1	-6.04478025	3.92343092	1.78733909	1	-0.40818012	-4.68946886	-2.28646088
1	-5.38108015	3.35693097	3.31943917	1	2.96771979	-6.20086908	0.54103911
6	-3.59758019	2.83373094	1.37873912	1	2.61401987	-5.19236898	-0.87506092
1	-2.84528017	2.20083094	0.90613914	1	3.08081985	-4.43096876	0.65043914
1	-3.17078018	3.23903084	2.30403924	8	0.44781989	3.57363081	-1.35486090
1	-3.80598021	3.67243099	0.70473909	1	-1.08428013	3.25953102	-2.62326097
6	-4.62567997	0.89833105	2.65783906	1	-1.46328008	2.81713080	-0.94676089
1	-5.56597996	0.39763099	2.91413903	8	1.82661986	-1.07126892	2.01263905
1	-4.18578005	1.28323102	3.58653903	15	1.27681994	-1.47826898	3.33813906
1	-3.95378017	0.15143099	2.23163915	8	-0.58138013	0.18803102	1.68413913
12	1.22661984	0.24933100	0.43493912	8	0.59781986	-0.35916901	4.22373915
8	-0.27248010	0.45603099	-0.96066082	6	-0.72768009	1.08033097	2.75203919
8	2.91731977	-0.17576900	-0.46106091	6	0.44321990	0.99873102	3.71363902
6	-0.34318012	1.31553102	-2.06296086	1	-1.65258014	0.87243104	3.31203914
6	-0.70328009	2.75743079	-1.73086083	1	-0.78018010	2.12883091	2.42093921
1	0.60431987	1.33443105	-2.61636090	1	1.37791991	1.31013107	3.24153924
6	3.97881985	0.17223099	-1.16256082	1	0.27401990	1.61273098	4.60073900
6	5.09902000	0.84593105	-0.55266094	8	2.39291978	-2.03996897	4.32153893
6	6.03942013	1.46123099	-1.38806081	6	3.30561996	-3.06096911	3.87913918
1	6.85491991	2.02953100	-0.95156085	1	2.77811980	-4.01266909	3.77063918

6	5.98562002	1.35313106	-2.77456093	1	3.77831984	-2.77086902	2.93943906
6	5.05381966	0.47983101	-3.33106089	1	4.05951977	-3.14836907	4.66073895
1	5.10551977	0.28913099	-4.39846087	15	1.13791990	3.60923100	0.07283911
6	4.09062004	-0.17356899	-2.55746078	8	1.35181987	2.28793097	0.72383916
6	5.35831976	0.79953104	0.96883917	8	2.42662001	4.48293114	-0.20686090
6	3.27371979	-1.32646894	-3.17726088	8	0.28091988	4.52733135	1.04403913
6	4.39391994	1.68163097	1.77693915	6	3.36781979	4.12383127	-1.25346088
1	3.36031985	1.36983097	1.64633918	6	-0.04598010	5.89973116	0.69373912
1	4.63461971	1.62783098	2.84693909	1	4.18892002	4.83283138	-1.16136086
1	4.46702003	2.73133087	1.47203910	1	3.73941994	3.10813093	-1.11046088
6	5.24071980	-0.66276896	1.44393909	1	2.88571978	4.22733116	-2.22696090
1	4.24631977	-1.05846894	1.23153913	6	-0.79588014	6.51033115	1.85573912
1	5.98521996	-1.28796899	0.93783915	1	0.88471991	6.43573093	0.48963910
1	5.42401981	-0.72356898	2.52503920	1	-0.65458012	5.89013100	-0.21556088
6	6.78251982	1.26733100	1.32313919	1	-1.05478013	7.54623127	1.61743915
1	7.54882002	0.70473105	0.77983916	1	-1.71908009	5.96173096	2.05753899
1	6.93571997	2.33433080	1.12743914	1	-0.18088010	6.50743103	2.75913906
1	6.95321989	1.11213100	2.39473915				

DI-5m

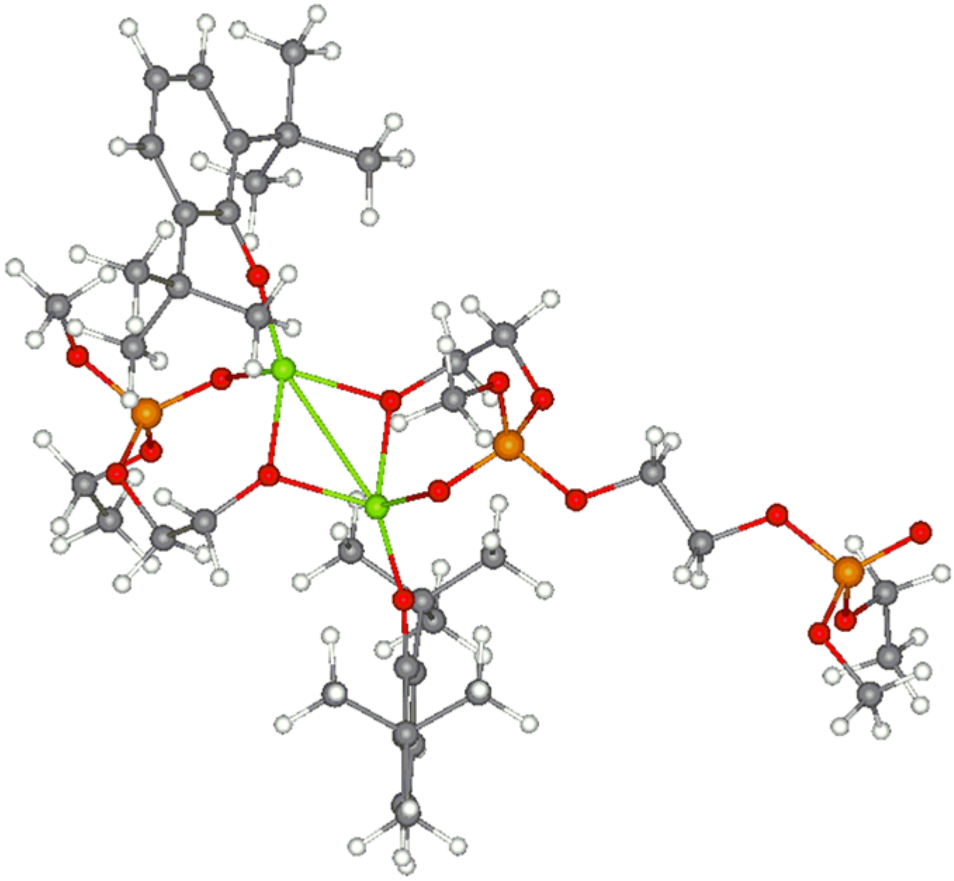

Zero-point vibrational energy	2979905.6 (Joules/Mol)
	712.21453 (Kcal/Mol)
Zero-point correction=	1.134986 (Hartree/Particle)
Thermal correction to Energy=	1.211475
Thermal correction to Enthalpy=	1.212419
Thermal correction to Gibbs Free Energy=	1.010057
Sum of electronic and zero-point Energies=	-4232.462628
Sum of electronic and thermal Energies=	-4232.386140
Sum of electronic and thermal Enthalpies=	-4232.385196
Sum of electronic and thermal Free Energies=	-4232.587557

cartesian

12	-0.22144972	0.61003995	-0.47804517	6	4.33225060	-3.94045997	-3.80264521
8	-8.55114937	-0.99805999	1.18885481	1	5.39775038	-3.82346010	-4.02634478
8	-1.21794975	2.18973994	-0.39924514	1	4.11545038	-5.00975990	-3.70924520
6	-8.63914967	-0.84736001	2.63055468	1	3.77755022	-3.56796002	-4.67144489
1	-7.67004967	-1.11386001	3.06555486	6	2.37795019	-3.33586001	-2.42644525
1	-9.39224911	-1.54226005	3.01095486	1	1.97695017	-2.80426002	-1.56284523
6	-1.68954980	3.43023992	-0.44734517	1	1.87545013	-2.96725988	-3.32974529

6	-2.41844988	3.88163996	-1.59604526	1	2.13715029	-4.39866018	-2.31194520
6	-2.83744979	5.21473980	-1.64104521	6	4.24825048	-1.67656004	-2.82944512
1	-3.37654972	5.58163977	-2.50814509	1	5.32375050	-1.56446004	-3.00614524
6	-2.59554982	6.10173988	-0.59764516	1	3.72135019	-1.31516004	-3.72114515
6	-1.94484973	5.64024019	0.54115486	1	3.97555041	-1.03666008	-1.98884523
1	-1.79394972	6.33633995	1.35945475	6	-8.99704933	0.59113991	2.93105483
6	-1.49344981	4.32193995	0.65715486	1	1.36685026	1.85944009	-2.83924532
6	-2.78454971	2.92903996	-2.75044513	1	6.99005032	-5.91525984	-0.34594515
6	-0.82274967	3.85743999	1.96375477	1	-2.92954969	7.13454008	-0.66244513
6	-3.63584971	1.76243997	-2.21054530	8	-9.32724953	-3.48476005	1.24715483
1	-3.11284971	1.20863998	-1.43144524	15	-8.51564980	-2.47406006	0.54985487
1	-3.88804984	1.06394005	-3.01844525	8	-6.96744967	-2.88195992	0.44925484
1	-4.57234955	2.14474010	-1.78914523	8	-3.69334984	-1.79535997	-0.58894515
6	-1.52254975	2.39274001	-3.45154524	6	-6.01814938	-2.04416013	-0.22534515
1	-0.88444972	1.84593999	-2.75644517	6	-4.65514946	-2.63005996	0.09225484
1	-0.93544972	3.21623993	-3.87384510	1	-6.08394957	-1.01416004	0.13645485
1	-1.79224980	1.71334004	-4.26924515	1	-6.20744944	-2.05726004	-1.30164516
6	-3.62354970	3.61953998	-3.84054518	1	-4.46664953	-2.61016011	1.16775477
1	-3.08604980	4.44233990	-4.32444477	1	-4.57814932	-3.65806007	-0.27164516
1	-4.56924963	4.00884008	-3.44944525	8	-8.84974957	-2.15646005	-0.98204517
1	-3.86934972	2.88894010	-4.61954498	6	-10.19794941	-1.79916000	-1.33994520
6	-0.79844970	4.95974016	3.03795481	1	-10.89174938	-2.58656001	-1.03694522
1	-1.80384982	5.30463982	3.30105472	1	-10.47334957	-0.84925997	-0.87454516
1	-0.20684972	5.82883978	2.72985482	1	-10.20524979	-1.69345999	-2.42424512
1	-0.33904970	4.56264019	3.95035481	1	-9.05714989	0.73464000	4.01415491
6	0.64465028	3.46194005	1.71595478	1	-8.24314976	1.27603996	2.53445482
1	0.73025030	2.64984012	0.99355483	1	-9.96594906	0.84943998	2.49565482
1	1.12565029	3.13463998	2.64505482	8	4.28005028	2.24354005	-1.20204520
1	1.20775020	4.31943989	1.33065474	1	3.13705015	3.32764006	-2.48104525
6	-1.60824978	2.67603993	2.56985474	1	2.39405012	3.11824012	-0.87844515
1	-2.62024975	2.99404001	2.84425473	8	-1.41714978	-0.89506006	-1.14194524
1	-1.11184978	2.31044006	3.47775483	15	-2.13094974	-1.97106004	-0.39154515
1	-1.70024979	1.85044003	1.86245477	8	0.61655033	-0.45626003	0.99015486
12	2.44315028	-0.49896002	0.22665484	8	-1.96314979	-2.01356006	1.17415476
8	1.61775029	0.59343994	-1.22774518	6	0.06905030	-1.02516007	2.14655471
8	3.41155028	-2.11145997	0.13485485	6	-0.70374972	-2.29895997	1.86505485
6	2.16265035	1.42724001	-2.21514511	1	-0.59864974	-0.31516004	2.65475488
6	2.94475031	2.61353993	-1.67664516	1	0.85415035	-1.29296005	2.86885476
1	2.81835032	0.86023998	-2.89054513	1	-0.11684972	-2.99916005	1.26585484
6	4.28315067	-3.10825992	0.01845484	1	-1.00914979	-2.78636003	2.79325485
6	4.89815044	-3.67765999	1.18335474	8	-1.72034979	-3.43635988	-0.85284513

6	5.86655045	-4.67165995	1.01155484	6	-1.46744978	-3.71975994	-2.24914527
1	6.35485029	-5.10275984	1.87895477	1	-2.40834975	-3.71846008	-2.80464530
6	6.23025036	-5.14415979	-0.24434516	1	-0.77814972	-2.98446012	-2.66504526
6	5.58535051	-4.63556004	-1.36554515	1	-1.01924968	-4.71116018	-2.27484512
1	5.85485029	-5.03885984	-2.33574510	15	4.59555054	1.93063998	0.32345486
6	4.60915041	-3.63845992	-1.27364516	8	3.68675017	0.91674006	0.93665487
6	4.49575043	-3.25286007	2.60825467	8	6.13055038	1.54903996	0.29875484
6	3.90335011	-3.15095997	-2.55264521	8	4.54955053	3.27083993	1.15585482
6	4.84765053	-1.77646005	2.86785483	6	6.57945061	0.31383997	-0.31464514
1	4.37455034	-1.10376000	2.15365481	6	5.41615057	4.39813995	0.84005487
1	4.53575039	-1.47586000	3.87605476	1	7.63615036	0.22963998	-0.06654516
1	5.93165064	-1.62795997	2.80005479	1	6.03175068	-0.53736007	0.09285484
6	2.98655033	-3.49695992	2.80835485	1	6.44995070	0.37393996	-1.39634526
1	2.40185022	-2.97995996	2.04635477	6	5.04735041	5.53694010	1.76235473
1	2.76125026	-4.56686020	2.73565483	1	6.45195055	4.07984018	0.98285484
1	2.66685033	-3.15165997	3.79985476	1	5.27105045	4.66784000	-0.21044515
6	5.21685028	-4.06845999	3.69635487	1	5.68925047	6.39703989	1.54995477
1	5.01425028	-5.14156008	3.61625481	1	4.00685024	5.83684015	1.61835480
1	6.30185032	-3.91966009	3.67815471	1	5.18705034	5.25053978	2.80745482
1	4.86365032	-3.74185991	4.68115520				

S2. Polymerization and polymer spectra

S2.1. Polymerization experiments

Homopolymerization at 5 °C (Table 1, runs 1-4).

Solution of [(BHT)Mg(μ-OBn)(THF)]$_2$ (**Mg1**, 75 mg, 177 μmol Mg) in CH_2Cl_2 (1 mL) was added to the cooled (5 °C) and stirred solution of the monomer (17.7 mmol) in CH_2Cl_2 (the total volume of the solution is 16.5 mL). The resulting mixture of 1M monomer concentration was stirred for 10 min. Then, 1 mmol of AcOH in CH_2Cl_2 (5 mL) was added, and the probe of the reaction mixture was analyzed by ^{1}H NMR spectroscopy. The product was separated by precipitation and filtration after the addition of Et_2O (90 mL) and was dried *in vacuo*.

NMR spectra of poly(εCL) and poly(MeOEP) are provided in Figures S2 and S3, respectively. NMR spectra of *rac*-LA and *L*-LA polymers are presented in the manuscript.

Homopolymerization of MeOEP at low temperatures (Table 1, runs 5 and 6).

The experiments were repeated using an external bath thermostatted at given temperature (–20 or –50 °C). The treatment of the mixture and separation of the polymer were pperformed as described above. NMR spectra are presented in Figures S4 and S5.

Prepolymerization of MeOEP with subsequent lactide polymerization (Table 1, runs 7 and 8).

MeOEP (0.239 g, 1.76 mmol) was dissolved in CH_2Cl_2 (0.6 mL). The mixture was cooled to 0 °C, and the solution of the **Mg1** catalyst (75 mg. 177 μmol Mg) in CH_2Cl_2 (1 mL) was injected. After 3 min of stirring, the lactide solution prepared from 2.309 g (16.02 mmol) of rac-LA or L-LA in CH_2Cl_2 (the total volume 14.2 mL) was injected into the reaction mixture. After 10 min of stirring, the polymerization was terminated by the addition of 1 mmol of AcOH in CH_2Cl_2 (5 mL). The reaction mixture was analyzed by ^{1}H NMR spectroscopy, and the product was separated by precipitation and filtration after the addition of Et_2O (90 mL) and was dried *in vacuo*.

^{1}H NMR spectra of copolymers are presented in the manuscript. ^{31}P NMR spectra are provided in Figures S6 and S7, respectively.

S2.2. NMR spectra of polymers

^{1}H NMR spectra of the reaction mixtures and polymer samples were recorded in $CDCl_3$ at 20 °C. M_n values of the polymers were determined by end-group analysis, by comparative integration of the CH_2OH end group for poly(εCL) or by initiator signals (benzyl group) for poly(MeOEP) and PLA.

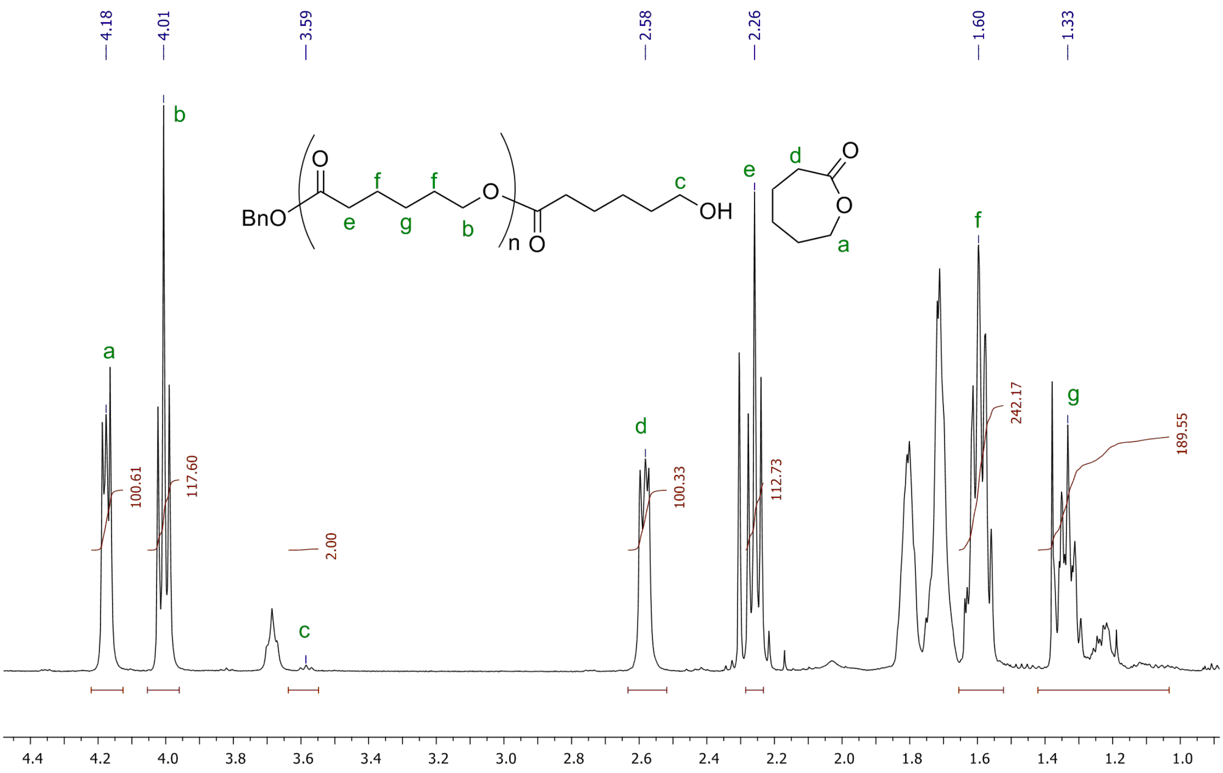

Figure S2. ^{1}H NMR spectrum ($CDCl_3$, 20 °C) of poly(εCL) (Table 3, run 1)

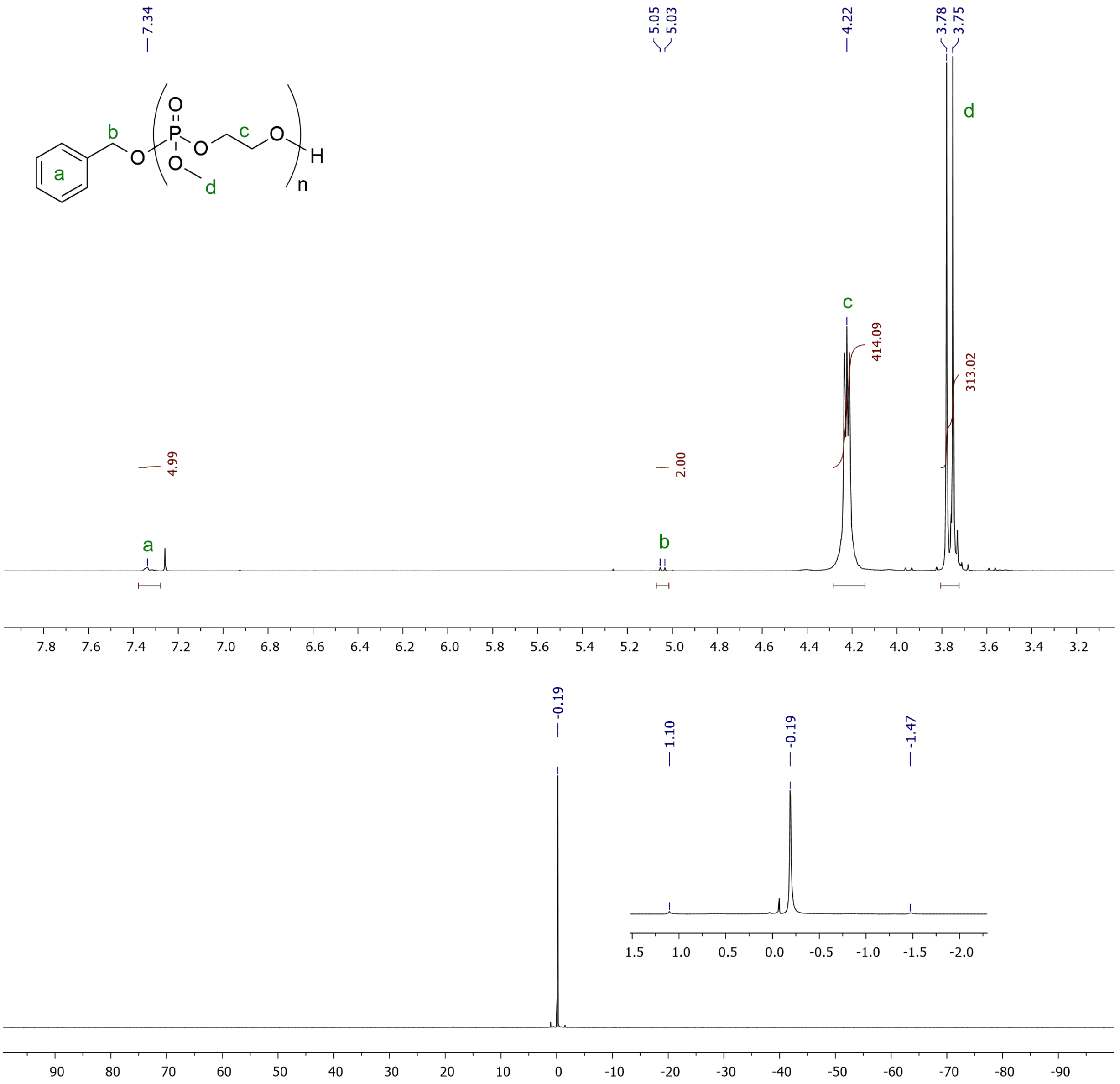

Figure S3. ^{1}H and ^{31}P NMR NMR spectra ($CDCl_3$, 20 °C) of poly(MeOEP) obtained at 5 °C (Table 3, run 4)

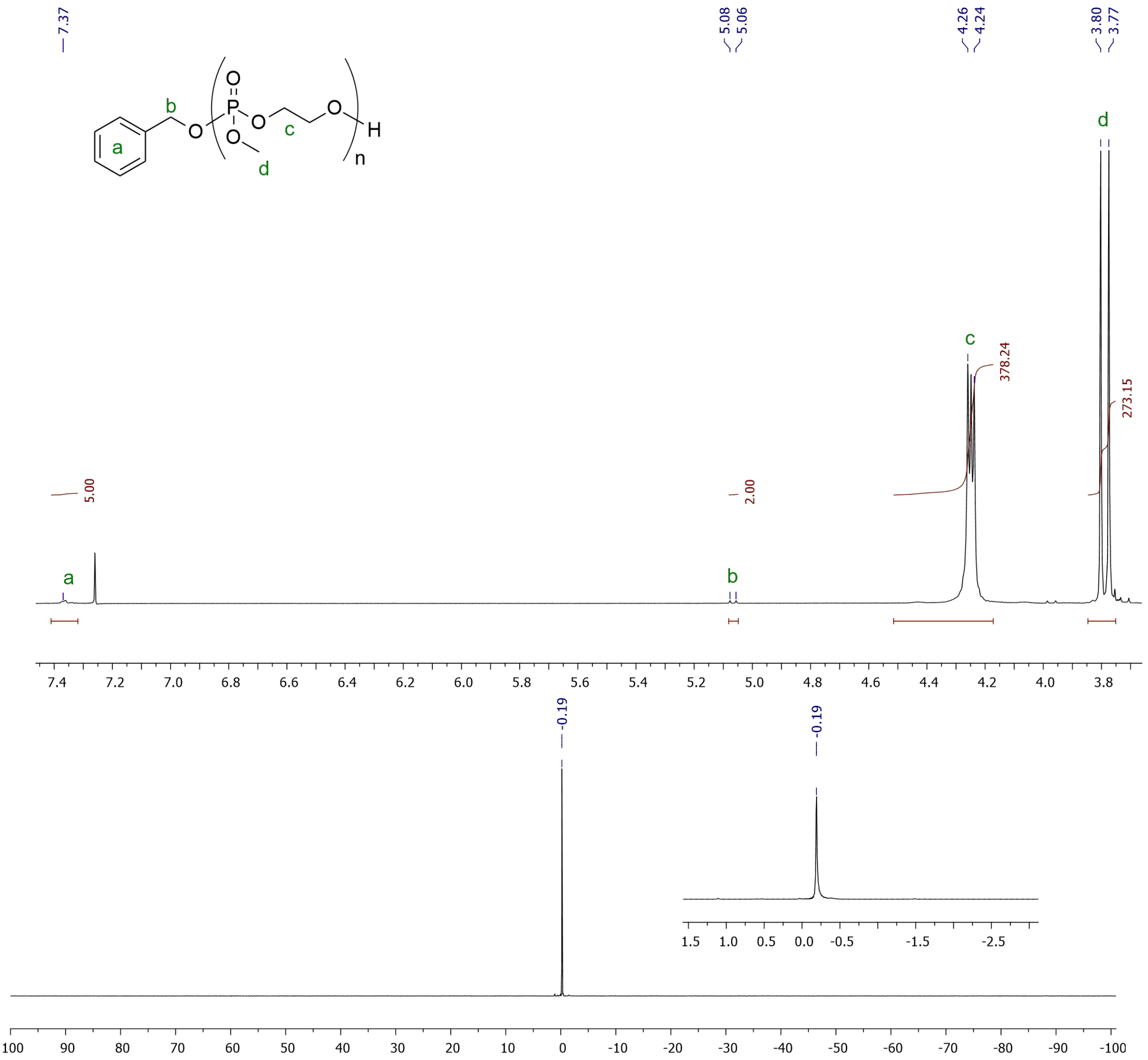

Figure S4. ^{1}H and ^{31}P NMR spectra ($CDCl_3$, 20 °C) of poly(MeOEP) obtained at –20 °C (Table 3, run 5)

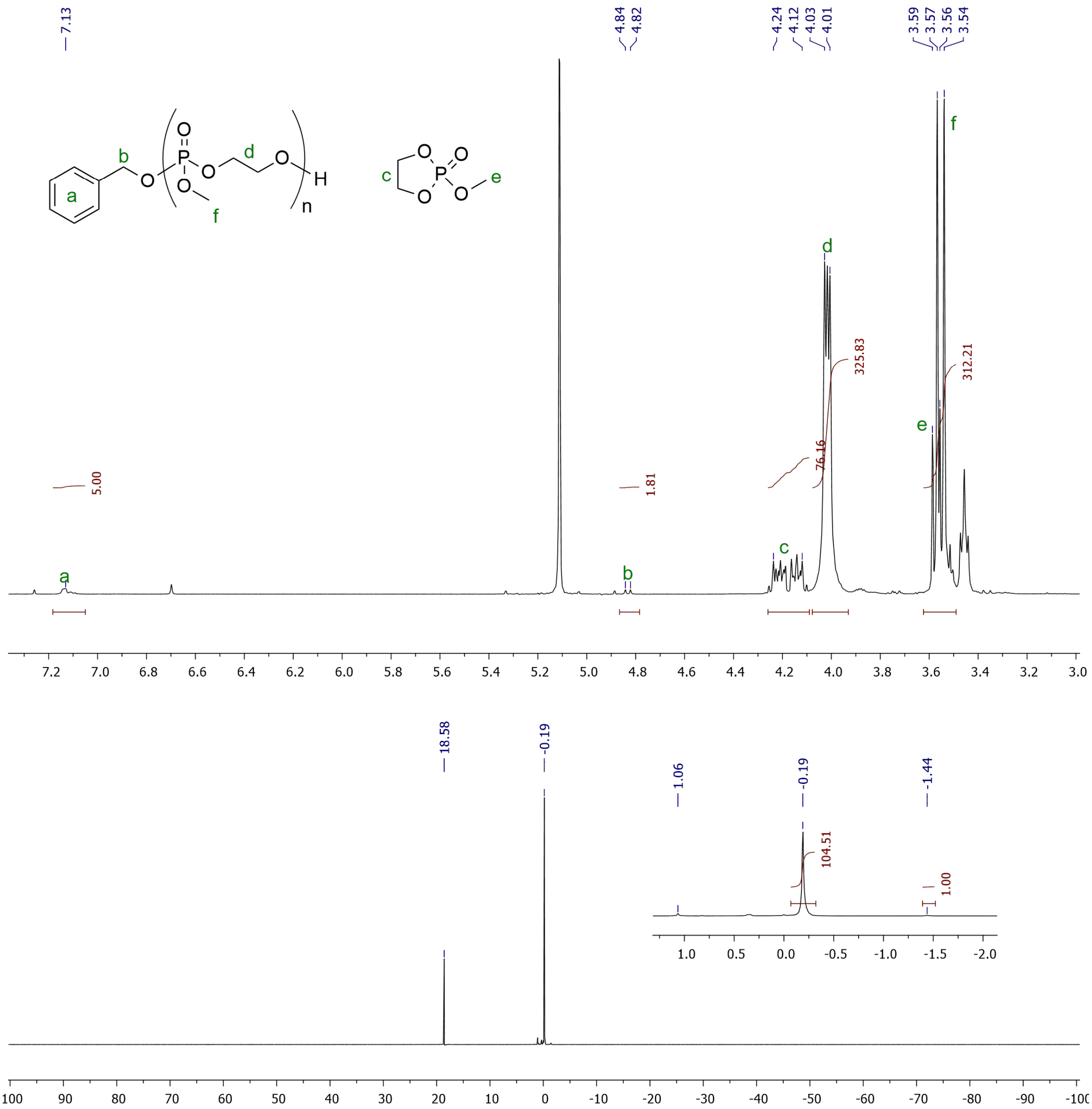

Figure S5. ^{1}H and ^{31}P NMR spectra ($CDCl_3$, 20 °C) of the reaction mixture of MeOEP polymerization at –50 °C (Table 3, run 6)

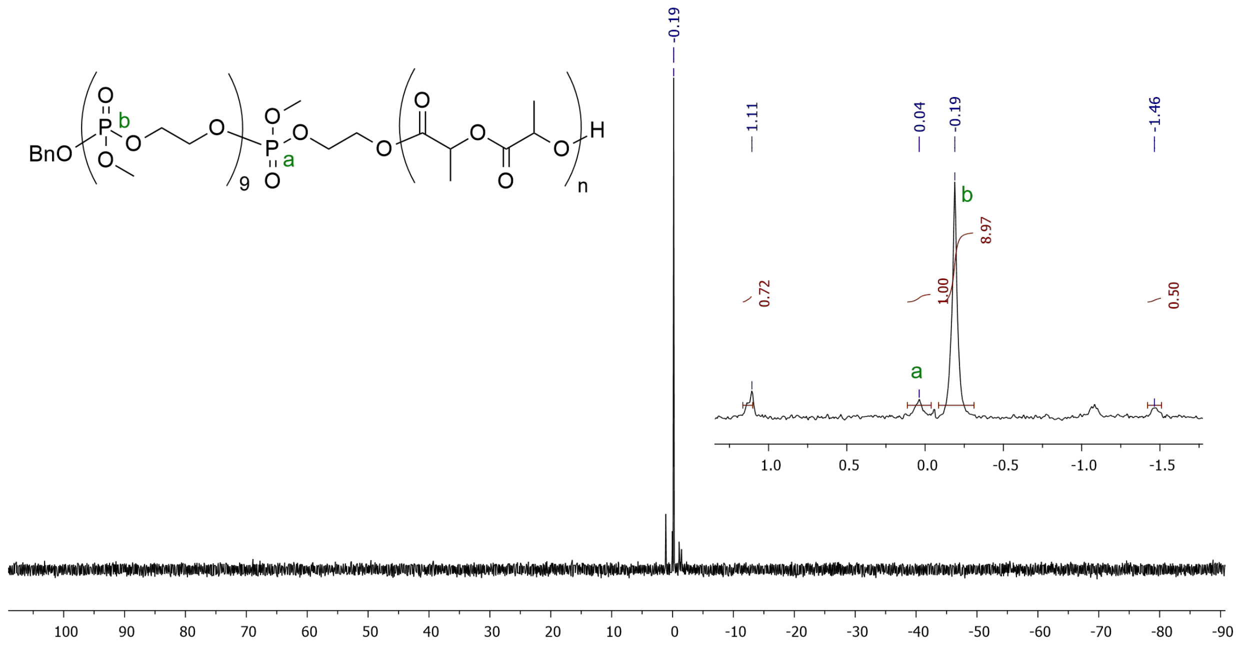

Figure S6. ^{31}P NMR spectrum ($CDCl_3$, 20 °C) of the reaction mixture obtained by *rac*-LA polymerization after MeOEP pre-polymerization (Table 3, run 7)

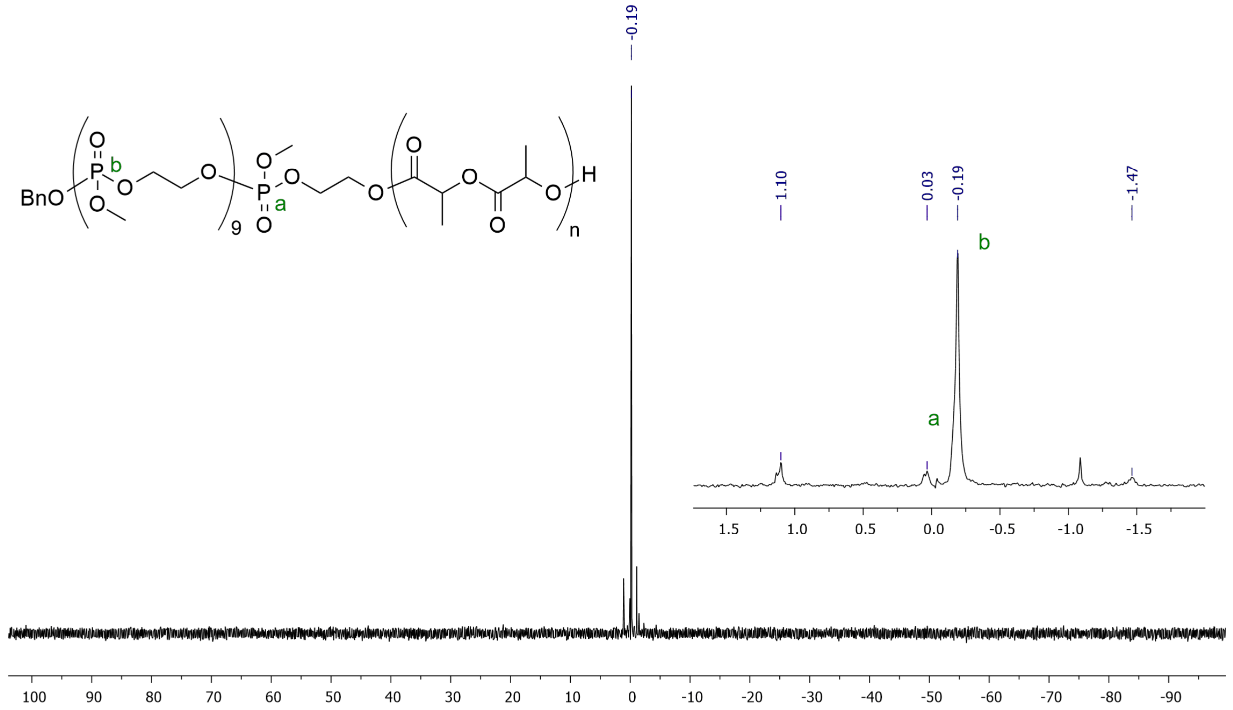

Figure S7. ^{31}P NMR spectrum ($CDCl_3$, 20 °C) of the reaction mixture obtained by *rac*-LA polymerization after MeOEP pre-polymerization (Table 3, run 8)

References

1. Frisch, M.J.; Trucks, G.W.; Schlegel, H.B.; Scuseria, G.E.; Robb, M.A.; Cheeseman, J.R.; Scalmani, G.; Barone, V.; Petersson, G.A.; Nakatsuji, H.; Li, X.; Caricato, M.; Marenich, A.; Bloino, J.; Janesko, B.G.; Gomperts, R.; Mennucci, B.; Hratchian, H.P.; Ortiz, J.V.; Izmaylov, A.F.; Sonnenberg, J.L.; Williams-Young, D.; Ding, F.; Lipparini, F.; Egidi, F.; Goings, J.; Peng, B.; Petrone, A.; Henderson, T. ; Ranasinghe, D.; Zakrzewski, V.G.; Gao, J.; Rega, N.; Zheng, G.; Liang, W.; Hada, M.; Ehara, M.; Toyota, K.; Fukuda, R.; Hasegawa, J.; Ishida, M.; Nakajima, T.; Honda, Y.; Kitao, O.; Nakai, H.; Vreven, T.; Throssell, K.; Montgomery, J.A., Jr.; Peralta, J.E.; Ogliaro, F.; Bearpark, M.; Heyd, J.J.; Brothers, E.; Kudin, K.N.; Staroverov, V.N.; Keith, T.; Kobayashi, R.; Normand, J.; Raghavachari, K.; Rendell, A.; Burant, J.C.; Iyengar, S.S.; Tomasi, J.; Cossi, M.; Millam, J.M.; Klene, M.; Adamo, C.; Cammi, R.; Ochterski, J.W.; Martin, R.L.; Morokuma, K.; Farkas, O.; Foresman J.B.; Fox, D.J. *Gaussian 09, Revision A.01*, Gaussian, Inc., Wallingford CT, **2016**.

2. Steitz, T.A.; Lipscomb, W.N. Molecular structure of methyl ethylene phosphate. *J. Am. Chem. Soc.* **1965**, *87*, 2488–2489, DOI: 10.1021/ja01089a031.

3. Nifant'ev, I.E.; Shlyakhtin, A.V.; Bagrov, V.V.; Minyaev, M.E.; Churakov, A.V.; Karchevsky, S.G.; Birin, K.P.; Ivchenko, P.V. Mono-BHT heteroleptic magnesium complexes: synthesis, molecular structure and catalytic behavior in the ring-opening polymerization of cyclic esters. *Dalton Trans.* **2017**, *46*, 12132–12146, DOI: 10.1039/c7dt02469j.
